# Genomic profiling of six human somatic histone H1 variants denotes that H1X accumulates at recently incorporated transposable elements

**DOI:** 10.1093/nar/gkae014

**Published:** 2024-01-23

**Authors:** Mónica Salinas-Pena, Núria Serna-Pujol, Albert Jordan

**Affiliations:** Molecular Biology Institute of Barcelona (IBMB-CSIC), Department of Structural and Molecular Biology, Barcelona 08028, Spain; Molecular Biology Institute of Barcelona (IBMB-CSIC), Department of Structural and Molecular Biology, Barcelona 08028, Spain; Molecular Biology Institute of Barcelona (IBMB-CSIC), Department of Structural and Molecular Biology, Barcelona 08028, Spain

## Abstract

Histone H1, a vital component in chromatin structure, binds to linker DNA and regulates nuclear processes. We have investigated the distribution of histone H1 variants in a breast cancer cell line using ChIP-Seq. Two major groups of variants are identified: H1.2, H1.3, H1.5 and H1.0 are abundant in low GC regions (B compartment), while H1.4 and H1X preferentially localize in high GC regions (A compartment). Examining their abundance within transposable elements (TEs) reveals that H1X and H1.4 are enriched in recently-incorporated TEs (SVA and SINE-Alu), while H1.0/H1.2/H1.3/H1.5 are more abundant in older elements. Notably, H1X is particularly enriched in SVA families, while H1.4 shows the highest abundance in young AluY elements. Although low GC variants are generally enriched in LINE, LTR and DNA repeats, H1X and H1.4 are also abundant in a subset of recent LINE-L1 and LTR repeats. H1X enrichment at SVA and Alu is consistent across multiple cell lines. Further, H1X depletion leads to TE derepression, suggesting its role in maintaining TE repression. Overall, this study provides novel insights into the differential distribution of histone H1 variants among repetitive elements, highlighting the potential involvement of H1X in repressing TEs recently incorporated within the human genome.

## Graphical Abstract

**Graphical Abstract:**
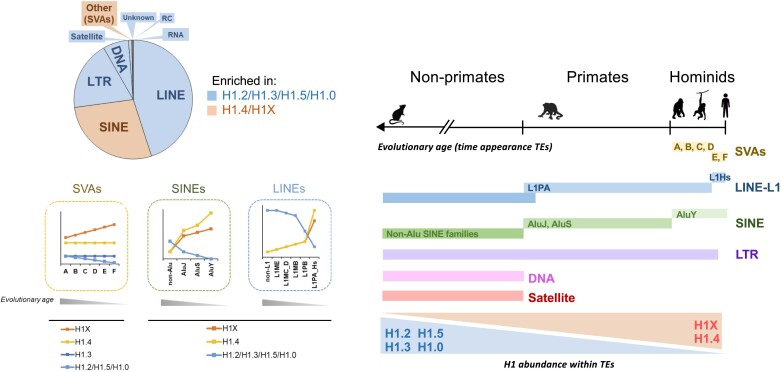

## Introduction

Repetitive elements can be found in almost all eukaryotic genomes. In humans, over half of the genome is composed of repetitive elements, which mainly include tandem repeats (for example satellites) and interspersed transposable elements (TEs). Different classes of TEs exist, exhibiting a variable transposition mechanism and structure. Retrotransposons (or Class I TEs) move throughout the genome via RNA intermediates while DNA transposons (or Class II TEs) move autonomously as DNA segments. Retrotransposon classes include long interspersed elements (LINEs) and short interspersed nuclear elements (SINEs, including Alu elements), which are the two major classes of repeats in the human genome. Other classes of retrotransposons are represented by SINE-VNTR-Alu elements (SVAs) and long-terminal-repeats (LTR) elements, such as endogenous retroviruses (ERVs) (1,2).

These TEs families have invaded the genome over millions of years of evolution (3). While recent insertions may be restricted to humans, older insertions were acquired ancestrally and are shared among different species. For instance, Alu elements have successfully expanded throughout primate genomes (4) while SVAs have been incorporated along the hominid lineage (5). However, the majority of TEs families have lost their transposition capacity (6,7). Multiple epigenetic mechanisms are involved in mediating TE silencing, including DNA methylation (8–13), repressive histone modifications (12,14) and the recruitment of transcriptional repressors such us HP1alpha, KAP1 or KRAB-KZNF proteins (15–19). On the other hand, TEs impact host gene-regulatory networks by acting as *cis-*regulatory elements, challenging the traditional an outdated notion of ‘junk DNA’ (20–24). Nonetheless, determination of chromatin landscape within repetitive elements, which can provide meaningful insights into their functional heterogeneity and regulation, remains limited. Additionally, other epigenetic mechanisms play a role in maintaining a homeostatic transcriptional state of repetitive elements, including linker histone H1.

Histone H1 family is evolutionary diverse and human somatic cells may contain up to seven H1 variants (H1.1 to H1.5, H1.0 and H1X). Although H1 has classically been implicated in the higher-order organization of the genome, promoting chromatin compaction (25,26), multiple studies support that H1 variants are not randomly distributed in the genome and display functional specificity (25,27–31). However, our understanding of this H1 diversity remains very incomplete.

A balanced histone H1 content is crucial to maintain chromatin structure. Simultaneous depletion of several H1 variants results in global chromatin decompaction in human cells (32) and various mice models (33–35). In T47D breast cancer cells, combined depletion of H1.2 and H1.4 (multiH1 KD), resulting in ≈ 30% reduction in the total H1 content, leads to genome-wide chromatin opening and the formation of more isolated but decompacted chromatin structures at the level of topologically associating domains (TADs) (31,32)In H1-triple knockout (TKO) mouse embryonic stem cells (mESCs), which lack H1c, H1d and H1e (H1.2, H1.3 and H1.5 orthologs; ≈50% reduction of total H1) local chromatin decompaction is observed and specific changes in the structural segmentation of chromosomes are accompanied by an increase of inter-domain interactions (33,36). Concretely, these compromised histone H1 content scenarios are also associated with aberrant expression of repetitive elements. In multiH1 KD cells, chromatin opening promotes the upregulation of repetitive elements, including satellites and ERVs (32). Their derived non-coding RNAs are sensed in the cytosol, ultimately triggering an interferon response. In H1 TKO mESCs, upregulation of major satellites is observed (33). Subsequent CRISPR-Cas inactivation of *Hist1h1a* (H1.1) and *Hist1h1b* (H1.5) in these H1 TKO mESCs leads to major satellites, LINE-L1 and ERVs des-repression (37). Notably, histone H1 and DNA methylation cooperatively repress TEs in plants (38).

Despite these inter-species functional association between histone H1 and repetitive elements regulation, differential H1 variants binding to the afore-mentioned repetitive elements classes has been scarcely explored. Indeed, H1-studies have been very limited by the lack of specific ChIP-grade antibodies. Consequently, different over-expression strategies have been employed to map H1 variants in both human and mice (28,29,39,40). However, only a few studies have investigated the genome-wide profiling of human endogenous H1 variants. In IMR-90 cells, H1.5 binding forms large chromatin blocks that correlate with transcriptionally inactive gene loci (41). In skin fibroblasts, H1.0 distribution correlates with GC content and is abundant at gene-rich chromosomes (42). In T47D, endogenous H1.2 and H1X show opposite genomic distributions (28–30). H1.2 is enriched within low GC regions while H1X is more prevalent within high GC regions. More recently, we performed the first genome-wide profiling of five endogenous H1 variants within a mammalian cell line (31). In T47D cells, H1 variants coexist in the genome in two large groups depending on the local GC content. H1.2 but also H1.0 and H1.5 are enriched towards low GC regions and inactive B compartment denoted by Hi-C experiments. H1.4 co-localizes with H1X at high GC regions and active A compartment, although H1X shows a stronger correlation with GC content compared to H1.4.

Here, we analyzed the genome-wide distribution of six endogenous H1 variants (H1.0, H1.2, H1.4, H1.5, H1X and also H1.3), with a particular focus on their differential binding patterns within repetitive elements classes and families. We found that H1 variants are selectively enriched within specific repetitive elements classes: H1.2, H1.3, H1.5, H1.0 are enriched within Satellite, LINE, LTR and DNA repeats, among others, while H1.4 and H1X are enriched within SINE and Other (i.e. SVAs) classes. Specifically, H1X is more strongly associated with SVA retrotransposons while H1.4 is preferentially linked to Alu elements. H1 variants association with other known TEs repressive mechanisms varies among different families, suggesting specific regulation of TEs. Moreover, H1 variants abundance within specific families shows a correlation with repeats evolutionary age. Thus, H1X and H1.4 are enriched within TEs recently incorporated in the genome along primates evolution, including youngest elements from SVAs, SINE-Alu, LINE-L1 and LTR classes/families. H1X depletion triggers a moderate transcriptional activation of these young TEs, supporting its role in TEs regulation. Notably, further examination of multiple cell lines showed that H1X is universally enriched within more recently evolved SVA and Alu repeats. This study represents the first comprehensive analysis of the complete H1 somatic repertoire within a mammalian cell type, providing a detailed H1 variants profiling within TEs families. Our findings support the non-random H1 variants enrichment within specific TEs in a variant-specific an evolutionary-related manner. Overall, we propose that H1 variants may specifically act as additional epigenetic determinants in repetitive elements silencing.

## Materials and methods

### Cell lines, culturing conditions and H1 variants knock-downs

Breast cancer T47D-MTVL derivative cell lines, which carry one stably integrated copy of luciferase reporter gene driven by the MMTV promoter, were grown in RPMI 1640 medium, supplemented with 10% FBS, 2 mM l-glutamine, 100 U/ml penicillin, and 100 μg/ml streptomycin, as described previously. SK-MEL-147, SK-N-SH, HeLa and HCT-116 cell lines were grown in DMEM GlutaMax medium, supplemented with 10% FBS and 1% penicillin/streptomycin. MCF-7 cell line was grown in MEM medium containing 10% FBS, 1% penicillin/streptomycin, 1% non-essential aminoacids, 1% sodium pyruvate and 1% l-glutamine. MDA-MB-231 cell line was grown in DMEM/F-12 medium containing 10% FBS, 1% penicillin/streptomycin and 1% l-glutamine. All cell lines were grown at 37°C with 5% CO_2_.

Doxycycline (Dox)-inducible shRNA H1 knock-downs (KD) were described in previous works (27,28,32). Concretely, T47D H1Xsh (28), T47D H1.4sh (32) cell lines were used to analyze single H1 depletion. A T47D derivative cell line containing a Randomsh RNA was used as a control (27). shRNA expression was induced with 6 days treatment of Dox, in which cells were passaged on day 3. Dox (Sigma) was added at 2.5 μg/ml. Untreated multiH1sh (also named multiH1 KD) (32) were used for the ChIP-seq experiments shown here, together with wild-type T47D as a replica.

### RNA extraction and reverse transcriptase (RT)-qPCR

Total RNA was extracted using the High Pure RNA Isolation Kit (Roche). Then, cDNA was generated from 100 ng of RNA using the Superscript First Strand Synthesis System (Invitrogen). Gene products were analyzed by qPCR, using SYBR Green Master Mix (Invitrogen) and specific oligonucleotides in a QuantStudio 5 machine (Applied Biosystems). Each value was corrected by human GAPDH and represented as relative units. Specific qPCR oligonucleotide sequences are listed in Supplementary Table S1. Chromatin enriched RNA (CheRNA) preparations were obtained as described in (43,44). Protocol procedure is detailed in Supplementary Methods.

### Chromatin immunoprecipitation (ChIP)

Chromatin immunoprecipitation was performed according to the Upstate (Millipore) standard protocol. Briefly, cells were fixed using 1% methanol-free formaldehyde for 10 min at 37°C, chromatin was extracted and sonicated to generate fragments between 200 and 500 bp. Next, 30 μg of sheared chromatin was immunoprecipitated overnight with the indicated antibody. Immunocomplexes were recovered using 20 μl of protein A magnetic beads, washed and eluted. Cross-linking was reversed at 65°C overnight and immunoprecipitated DNA was recovered using the IPure Kit (Diagenode). Genomic regions of interest were identified by real-time PCR (qPCR) under the same conditions described for RT-qPCR. Each value was corrected by the corresponding input chromatin sample.

### Antibodies

Specific antibodies recognizing human H1 variants used for ChIP/ChIP-Seq and immunoblot were: anti-H1.0/H5 clone 3H9 (Millipore, 05–629-I), anti-H1.2 (abcam, ab4086), anti-H1.3 (abcam, ab203948), anti-H1.4 (Invitrogen, 702876), anti-H1.5 (Invitrogen, 711912) and anti-H1X (abcam, ab31972). The variant specificity of anti-H1.3 has been extensively validated in this study (Supplementary Figure S1). Validation of the other anti-H1 antibodies has been reported elsewhere (31).

### RNA-Seq

*Library preparation:* Total RNA-Seq on T47D H1Xsh ± Dox cells and Che-RNA-Seq in T47D were performed. According to the manufacturer's instructions, the first step involves the removal of ribosomal RNA (rRNA) using target-specific oligos and RNase H reagents to deplete both cytoplasmic (5S rRNA, 5.8S rRNA, 18S rRNA and 28S rRNA) and mitochondrial ribosomal RNA (12S rRNA and 16S rRNA) from total RNA preparations. Following SPRI beads purification, the RNA is fragmented into small pieces using divalent cations under elevated temperature. The cleaved RNA fragments are copied into first strand cDNA using reverse transcriptase and random primers, followed by second strand cDNA synthesis using DNA Polymerase I and RNase H. This process removes the RNA template and synthesizes a replacement strand, incorporating dUTP in place of dTTP to generate ds cDNA. These cDNA fragments then have the addition of a single ‘A’ base and subsequent ligation of the adapter. After UDG treatment, the incorporation of dUTP quenches the second strand during amplification. The products are enriched with PCR to create the final cDNA library. The libraries were assessed quality and quantity in two methods: check the distribution of the fragments size using the Agilent 2100 bioanalyzer and quantify the library using real-time quantitative PCR (QPCR) (TaqMan Probe). The qualified libraries were sequenced pair end on the BGISEQ-500/ MGISEQ-2000 System (BGI-Shenzhen, China).

*RNA-Seq and CheRNA-Seq processing:* RNA-Seq reads were mapped to the human reference genome (GRCh37/hg19) using HISAT2 v2.2.1 (45) with default parameters and specifying strand-specific information (–rna-strandness RF). SAMtools v1.11 (46)was used to sort BAM files and filter for properly paired-end reads (-f 2). Aligned reads were mapped to Ensembl GRCh37.87 gene annotation with TEtranscripts v2.1.4 (–sortByPos –mode multi –stranded reverse) (47). DESeq2 v1.26.0 (48)was used to identify differentially expressed genes between WT and H1X KD cells. Moderated log2 fold change values were calculated by applying the shrinkage method, which is useful for ranking and visualization without the need for arbitrary filters on low count repeats. The Benjamini-Hochberg (BH) method was used to correct for multiple testing and control the proportion of false positives or FDR. Gene expression changes were considered significantly different if the absolute value of the log2(FC) was higher than 1.4 and the adjusted *P*-value was lower than 0.05. With respect to the quantification of individual repeats expression measured by RNA-Seq experiments, featureCounts v2.0.1 (49)was used to assign stranded paired-end reads to repetitive sequences included in the TEtranscripts (v2.1.4) annotation (47). More precisely, two different sets of parameters were used to work with only uniquely mapped reads (-f -p -s 2 -F GTF) or to include multi-mapping reads (-f -p -s 2 -F GTF -M – fraction) in the calculations, according to the needs of the analyses.

### ChIP-Seq

*Library construction and sequencing:* Qualified ChIP and Input samples were subjected to end-repair and then 3′ adenylated. Adaptors were ligated to the ends of these 3′ adenylated fragments. Fragments were PCR-amplified and PCR products were purified and selected with the Agencourt AMPure XP-Medium kit. The double stranded PCR products were heat denatured and circularized by the splint oligo sequence. The single strand circle DNA (ssCir DNA) were formatted as the final library and then quality-checked. The library was amplified to make DNA nanoball (DNB) which had >300 copies of one molecular. The DNBs were loaded into the patterned nanoarray and single end 50 bases reads were generated in the way of sequenced by combinatorial Probe-Anchor Synthesis (cPAS).

ChIP-seq data for the H1 variants was obtained in independent duplicates (different cells: wild-type T47D and untreated multiH1 KD T47D cells, different day of preparation and sequencing), respectively referred as replicates r1 and r2 in Supplementary Figure S2. Analysis were performed with the two replicates separately and after confirming that results were equivalent, data for replicate r2 is presented throughout the manuscript.

*ChIP-Seq data processing*: Single-end reads were quality-checked via FastQC (v0.11.9) and aligned to the human GRCh37/hg19 reference genome using Bowtie2 (v2.3.5.1) (50) with default options. SAMtools (v1.9) (46)utilities were used to filter out the low-quality reads with the flag 3844. Genome coverage was calculated and normalized by reads per million with BEDTools (v2.28.0) (51), and regions with zero coverage were also reported in the ChIP-Seq annotation (genomecov - ibam -bga -scale). MACS2 (v2.1.2) (52)was used to subtract input coverage from ChIP values to generate signal tracks (bdgcmp -m subtract). In addition, with the aim of only working with uniquely mapped reads when specified, SAMtools was used to filter ChIP-Seq reads by mapping quality (-q 4). The subsequent steps (genome coverage calculation and input subtraction) were equally computed with BEDTools and MACS2. The read count report of H1 variants ChIP-seq data is shown in Supplementary Tables S2 and Supplementary S3. When analysis was done at TE group level (meta-repeat profiles and heatmaps), multi-mapping reads were used because copies of recently incorporated TEs have not diverged enough to efficiently assign a unique position, but when analyses needed to recover copy-information (browser snapshots, changes in repeats expression, or correlation analysis), only uniquely-mapped reads were used.

### Methylated DNA immunoprecipitation sequencing (MeDIP-Seq) analysis

Input and MeDIP samples from GEO accession number GSE80175 were reprocessed for this study. Bowtie2 (v2.3.5.1) (50)was used to map single-end reads to the hg19 human reference genome. SAMtools (v1.9) (46)utilities were used to filter out reads with a mapping quality score lower than 10 (-q 10) and only keep uniquely mapped reads. The resulting BAM files were sorted and deepTools (v3.5.1) was used to generate input-subtracted and counts per million (CPM) normalized signal tracks (bamCompare –operation subtract – normalizeUsing CPM –scaleFactorsMethod None).

### ChIP-Seq downstream analysis

H1 variants and ZNF91 narrow peak calling was performed with MACS2 *(callpeak –no model –ext size 200)*, using multi-mapping reads. Usage of unique reads for peak calling lead to the underestimation of recent TEs and was discarded. H1X and H1.4 peak calling results in the different repetitive elements classes in shown in Supplementary Figure S4C. H1 peaks positions were ramdomized on the same chromosome using *bedtools shuffle* function, excluding BlackList regions and not allowing overlapping between the random segments (*-chrom -noOverlapping*). SICER (v1.1) (53)was used to identify larger H1X enriched regions or ‘islands’ with the following parameters: redundancy threshold = 1, window size = 200, fragment size = 150, effective genome fraction = 0.80, gap size = 200 and FDR = 0.01. H1 variants ChIP-Seq profiles at meta-repeats were constructed and visualized using deepTools (v3.5.1) (*computematrix, plotHeatmap, plotProfile*) (54). All regions were scaled to the same size (*scale-regions* mode). Heatmaps were performed by using the R package *pheatmap*. The euclidean distance measure and the complete cluster method were used in clustering rows and columns. Correlation matrices were computed by the R function cor() (method = ‘spearman’) and visualized using the R package corrplot. Only correlations with *P*-value <0.01 were considered, corresponding to the colored squares in the correlation matrices.

### Repetitive elements annotation and classification

TEtranscripts (v2.1.4) repetitive elements annotation was used (47). In TEtranscripts, GTF files of transposable element annotation were generated from the RepeatMasker tables obtained from UCSC genome database. Annotation includes nine different classes of repeats, including: LINE, SINE, LTR, DNA, Satellite, Other (SVA), Unknown, RC (Rolling-Circle) and RNA. Repeats with unsure classification named with a ‘?’ at the end of the family or class name (e.g. SINE?) were excluded from the analysis. Repeats overlapping problematic regions defined in ENCODE BlackList (55) were also excluded from the analysis. Number and genome occupancy of families and repeats belonging to each repetitive element class are included in Supplementary Figure S3.

Repetitive elements were classified from an evolutionary perspective, based on the taxonomic clades or species in which the repeat is known to be present, according to Dfam database (56). Clades classification is done according to NCBI taxonomy database. The following clades groups were considered in the analysis: Non-Primates, Primates, Simiiformes, Catarrhini, Hominoidea, Hominidae, Homo sapiens. This classification refers to the oldest clade in which copies of the family/repeat have been found at orthologous positions, meaning that the transposable element was active before the first speciation of extant species in this clade.

For all analysis done at class-family-repeat level, without considering concrete individual copies, reads that align to multiple positions (multi-reads) are also used for repeats quantification. When correlation between different datasets was evaluated at concrete copies/insertions, only uniquely mapped reads are considered.

### Public genomic data used

ChIP-Seq replicates of H1.0, H1.2, H1.4, H1.5 and H1X in T47D cells from our previous publication (31) are accessible through GEO Series accession numbers GSE156036 and GSE166645. T47D cells ATAC-Seq (GSE100762) and A/B compartment segmentations from Hi-C experiments (GSE172618) were also used. For ATAC-Seq and Hi-C analysis details see (30,31).

Other publicly available ChIP-Seq datasets used for the analysis were: H3K9me3 (T47D, GSE143653), H3K27me3 (T47D, GSE63109), ZNF91 (HEK293, GSE162571), ZNF93 (HEK293T, GSE78099), KAP1 (H1-hESC, GSE57989), RNA pol II (T47D, GSE120162), RNApol III (T47D, GSE120162), p300 (T47D, GSE68359), H3K4me2 (T47D, GSE63109), H3K36me3 (T47D, GSE63109), H3K9ac (T47D, GSE120162), H3K27ac (T47D, GSE120162), H4K20me3 (T47D, GSE143653). All ChIP-Seq datasets were processed as described. Genome-wide DNA methylation was assessed by MeDIP-Seq (MDA-MB-231, GSE80175).

## Results

### H1 variants are differentially enriched within repeat classes

In order to understand whether the different somatic histone H1 variants play different roles in the nuclei we have profiled its distribution within the genome of breast cancer cells by ChIP-Seq with variant-specific antibodies. As mentioned above, we previously described H1.0, H1.2 and H1.5 to be abundant at low GC regions and at the B compartment of the 3D genome, among others, whereas H1.4 and H1X were enriched at high GC regions and at the A compartment, compared to the other variants (31). We have now added H1.3 to this profiling after testing the specificity and ChIP validity of a new available antibody recognizing this variant (Supplementary Figure S1). H1.3 was found to be enriched within heterochromatin and low activity chromatin, low GC regions and the B compartment, similarly to H1.0, H1.2 and H1.5, and was depleted from the TSS of active genes as reported for the other variants (Supplementary Figure S2A-D) (31). ChIP-seq experiments were performed in two different breast cancer T47D cell line derivatives: wild-type and untreated multiH1 KD cells (replicas r1 and r2, respectively). A genome browser snapshot of the six H1 variants ChIP-seq data is shown in Supplementary Figure S2E. All together is the first reported profiling of six endogenous H1 variants within a mammalian cell type and without the expression of recombinant tagged H1 histones; also the first genome-wide mapping of endogenous H1.3 in any cell type. From now on data with replica r2 is shown, after confirming that results were equivalent with the two replicas.

**Figure 1.**
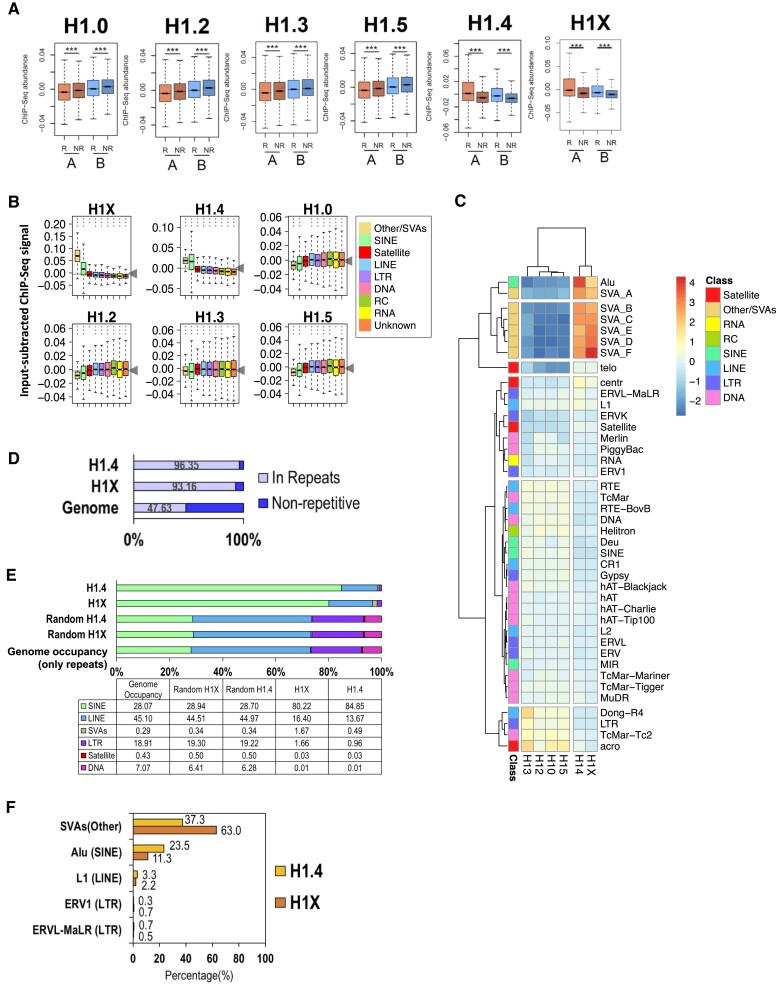
H1 variants are differentially enriched within repeat classes. (**A**) Boxplot of H1 variants input-subtracted ChIP-Seq abundance (T47D cells) within repetitive (R) or non-repetitive (NR) genome fractions. Intersection between A or B compartment segments and repetitive element annotation was calculated and each intersected region was classified as R or NR depending on their overlap or not with any type of repeat. Statistical differences between H1 abundance at R and NR fractions of each compartment are supported by One-sample Wilcoxon signed-rank test (***) *P*-value < 0.001. (**B**) Boxplot of H1 variants input-subtracted ChIP-Seq abundance within repetitive element classes (*N* = 9). In each boxplot, gray horizontal line (and arrow) corresponds to the median abundance of the corresponding H1 variant within all repeats. Kruskal–Wallis test determined that there were statistically significant differences between the classes (*P*-value < 0.001). One-sample Wilcoxon signed-rank test was used to statistically support H1 enrichment (blue asterisks) or depletion (red asterisks) compared to the median H1 abundance within all repeats. (***) *P*-value < 0.001; (**) *P*-value < 0.01; (*) *P*-value < 0.05. (**C**) Heatmap and cluster analysis of the average input-subtracted ChIP-Seq abundance (scaled) of H1 variants (T47D cells) within repeat elements families (excluding the Unknown class families). Each cell in the heatmap represents the scaled input-subtracted ChIP-seq average abundance for the respective family of repeats. The Y-axis annotation indicates the class to which each indicated family belongs. (**D**) Percentage of H1 narrow peaks that overlap or not with any type of annotated repeat. Minimum 50% of the length of the peak is required to overlap a repeat to be considered. The percentage of the human genome occupied by repeats is shown for comparison. (**E**) Number of peaks in repeats per repetitive element class, calculated as number of peaks in each class divided by number of peaks in repeats, and expressed as percentage. Genome occupancy of repeats, considering as 100% the 48.78% of the genome which is repetitive is shown as a reference. As a control, analysis was done with randomized H1 peak positions. (**F**) Percentage of repeats within each indicated class or family that contains at least one H1.4 or H1X peak.

Because nearly half of the human genome is occupied by repetitive DNA and transposable elements, we have focused here on investigating whether the different H1 variants occupy redundantly or specifically these elements. The GRCh37 GENCODE’s annotation contains >4.4 million repeats which can be hierarchically organized in classes (*N* = 9), families (*N* = 70) and repeat groups (*N* = 966) (Supplementary Figure S3A, B). These repeats occupy 47.6% of the genome base-pairs, with similar proportion within the A and B compartments (Supplementary Figure S3C). The differential abundance of histone H1 variants within these repetitive elements has not been explored before. First, we analyzed the ChIP-Seq abundance of H1 variants within repetitive and non-repetitive DNA, distinguishing whether they were present within the A or B compartments of the 3D genome (Figure 1A). H1.0/H1.2/H1.3/H1.5 variants were more abundant within the B compartments, and within non-repetitive DNA more than repetitive DNA in both compartments. Instead, H1.4/H1X were enriched at the A compartment and within repetitive DNA in both compartments. This is in agreement with our previous report that H1X was enriched within coding regions (which are enriched within the A compartment) (28), but shows that is more abundant within the repetitive elements present within coding regions than in the non-repetitive DNA (Supplementary Figure S2F).

Repetitive elements (REs) classes are differently distributed within A and B compartments (Supplementary Figure S3D). For example, Satellite, LINE and LTR classes are preferentially located in the B compartment while SINE or ‘Other’ classes are mostly found at A compartment. The Other class comprises SINE-VNTR-Alus (SVA) retrotransposons. The 9 repeat classes also occupy different fractions of our genome, being LINE and SINE elements the predominant ones (Supplementary Figure S3E). Due to the differential genome-wide distribution of repetitive elements classes, we aimed to explore the H1 variants abundance at different classes and families of repeats or transposable elements (TEs).

Analysis of ChIP-Seq H1 variants abundance within repeat classes revealed that H1.4 and H1X were highly enriched at SINE and SVA classes, which are those enriched within the A compartment (Figure 1B and Supplementary Table S3). Among both H1 variants, H1X was preferentially enriched at SVA elements, while H1.4 relative enrichment at SINE class was higher. On the other hand, H1.0, H1.2, H1.3 and H1.5 variants were more abundant within LINE, LTR, DNA, RNA, RC and ‘Unknown’ repeats. Among the repeat families, H1.4 and H1X were enriched within Alu (SINE) and SVA families, whereas H1.0, H1.2, H1.3 and H1.5 were enriched at the rest of families (Figure 1C).

To further analyze H1.4 and H1X abundance within repeats, we computed narrow peak calling using MACS2 software. Of note, this method can only be applied with these two variants, as the low GC H1 variants form large genomic domains and peaks cannot be efficiently called (Supplementary Figure S4A, B). Surprisingly, almost all H1.4 (96.35%) and H1X (93.16%) peaks overlapped with some repetitive element (Figure 1D). Of these peaks in repeats, the majority of them overlapped SINE elements but also included LINE and, to a lesser extent, SVA and LTR classes (Figure 1E). Compared to the occupancy of these repeats within the human genome (or to randomized H1 peak locations), it was evident that H1.4 and H1X are enriched at SINE and SVA above expected frequencies (Figure 1E). Notably, SINE repeats with H1.4 or H1X peaks mostly belonged to the Alu family while LINE were almost entirely represented by L1 family. For their part, peaks within LTR class accumulated in ERV1 and ERVL-MaLR families (Supplementary Figure S4C). We also used an alternative peak calling method (DROMPA (57)) obtaining equivalent results. In this case some peaks for the low GC H1 variants were obtained and found to be located at SINE and SVA below expected frequencies, contrary to H1X/H1.4, and enriched at LINE, LTR, Satellite and DNA TEs (Supplementary Figure S4D–G).

However, the calculation of the percentage of H1 peaks within repetitive classes is influenced by genome occupancy of each class (Figure 1E). Therefore, we also calculated the percentage of SVAs, Alu, LINE-L1, LTR-ERV1 and LTR-ERVL-MaLR elements marked with some H1 peak (Figure 1F). Interestingly, 63% of SVA repeats contain (at least) a peak of H1X, while 37% of them showed an H1.4 peak. These percentages are variable between SVA families, for example ≈80% of SVA_C or SVA_D contain some H1X peak and only 35.6% of SVA_F do (Supplementary Figure S5A). On the contrary, Alu elements were more associated to H1.4 compared to H1X, supporting the preferential binding of H1.4 to Alu elements, compared to H1X, as denoted by previous analyses (Figure 1B, C). While 23.5% of Alu elements showed an H1.4 peak, 11.3% of them exhibited an H1X peak (Figure 1F). These percentages are also variable among particular Alu elements, for example 50% of AluYa2 contain H1.4 and 20% contain H1X. Abundance of H1 peaks is higher in Alu elements belonging to the AluY subfamily (Supplementary Figure S5B-C). In relation to LINEs, only 2–3% of L1 elements showed some H1 peak (Figure 1F). These H1 peaks were accumulated (98%) within a limited subset of LINE-L1 elements among the 122 L1 repeats that comprise the family (58,59) (Supplementary Figure S5D). Lastly, <1% of ERV1 and ERVL-MaLR LTR contain some H1 peak, but interestingly, these peaks accumulated within very few LTR repeats (Figure 1F and Supplementary Figure S5E). The LTR repeats more extensively marked by both H1.4 and H1X were LTR12C, LTR12E and LTR12D. Concretely, ≈37% of LTR12C repeats contain some H1X peak whereas ≈16% of them exhibited H1.4 peaks. Comparing both variants, H1X was more associated to ERV1 family while H1.4 was more related to ERVL-MALR.

The genomic annotation of H1X or H1.4 peaks intersecting with different TE families where they are located showed considerable similarity between the two variants and differences between TEs. H1X/H1.4 peaks within Alu were 51% within introns, whereas peaks within LTR-ERV1 were 72–75% at intergenic regions (Supplementary Figure S5F). These percentages correspond to the annotation of total TEs of each group, indicating that H1 peaks are not enriched in any particular genomic category compared to their host element.

Further, using the ChIPAtlas database (60) of transcription factors (TF) binding across the entire genome in T47D cell line, we analyzed the TF binding profile of H1X/H1.4 narrow peaks located in different TE families. For comparison, we also checked the TF binding of the repeat families or specific repeat subsets from each family in which H1 peaks concentrate, serving as control regions. In all cases, the distribution of TF binding regions within H1 peaks resembled the TF distribution found in each of the control TE groups where H1 peaks were located (Supplementary Figure S5G). Despite these similarities, putative exceptions were also noticed. H1 peaks located within L1 repeats were enriched on PARP1 and CTF3C2 compared to control L1 repeats. H1 peaks within Alu were more bound by CHD8 in comparison with control Alu repeats. FOXA1 was under-represented in H1 peaks located at ERV1 compared to control ERV1 repeats. In general, TF binding profile of H1X and H1.4 peaks was very similar, except for those peaks located within L1 elements.

In general, analysis of H1 variants ChIP-Seq data denote their differential presence within concrete repeat classes. H1.4 and H1X are highly enriched within SINE and SVA classes. Exploration of these variants allow us to conclude that narrow enrichment peaks are concentrated within some particular groups of repeats. However, peaks cannot be computed efficiently for other H1 variants. For those reasons, we decided to apply alternative approaches to compare H1.4 and H1X with low GC variants (H1.0, H1.2, H1.3 and H1.5) differential abundance within repeat classes.

The abundance of H1 variants profiled in T47D cells was calculated in a meta-repeat for each class of repeats flanked by 3-kb regions. A heatmap for scores associated with the genomic regions of interest was also computed (Supplementary Figure S6). SINE and Other/SVA classes were clearly enriched in H1X and H1.4 and depleted of H1.0, H1.2, H1.3 and H1.5. On the other hand, H1.4/H1X were depleted from the other classes of repeats and also from their flanking regions when compared to H1.0, H1.2, H1.3 and H1.5 abundance. Interestingly, for the LINE, LTR and DNA classes, a local enrichment of H1.4/H1X immediately flanking the meta-repeat was observed. Overall, this analysis provides evidence that the selective enrichment of H1 variants within repeat classes is attributed to specific binding preferences to the repeat elements, rather than simply being determined by the overall abundance of H1 in the genomic region where the repeat is located. For that reason, we next explored into detail each class of TEs separately.

### H1X is highly enriched at all the SVA retrotransposon families

**Figure 2.**
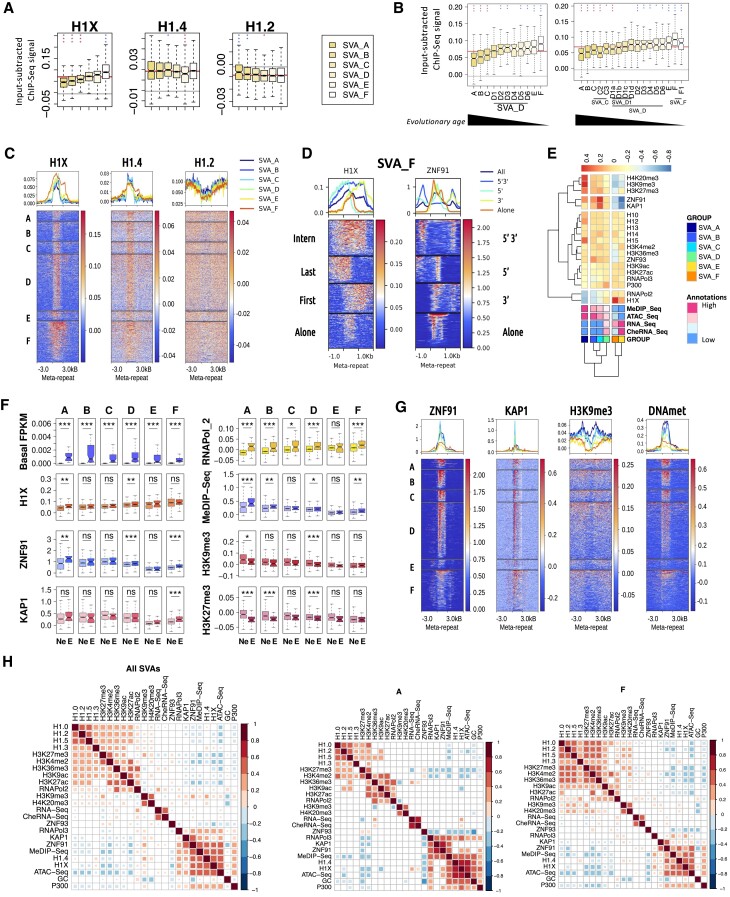
H1X is enriched at SVA transposable elements. (**A**) Boxplot of H1 variants input-subtracted ChIP-Seq abundance within the six families of the Other class (SVA_A to SVA_F, ordered by evolutionary age). In each boxplot, gray horizontal line corresponds to the median abundance of the corresponding H1 variant within all repetitive element classes (*N* = 9). Red horizontal line indicates median abundance of the corresponding H1 variant within all SVA repeats. Kruskal–Wallis test determined that there were statistically significant differences between the SVA families (*P*-value < 0.001). One-sample Wilcoxon signed-rank test was used to statistically support H1 enrichment (blue asterisks) or depletion (red asterisks) compared to the median H1 abundance within all SVA repeats. (***) *P*-value < 0.001; (**) *P*-value < 0.01; (*) *P*-value < 0.05. (**B**) Boxplot of H1X input-subtracted ChIP-Seq abundance within the SVA subfamilies established by Levy *et al.* (61). Classification is done applying two different threshold parameters as defined in the original paper, which results in *N* = 11 (left) or *N* = 17 (right) SVA sub-families. In general, original SVA_D, the majority SVA family, is first subdivided into six sub-families. SVA_D1, the majority sub-family of SVA_D can be further sub-divided into D1a-D1d sub-families. All sub-families are ordered by their evolutionary age. Statistics were performed as in (A). (**C**) Meta-repeat profile of H1 variants input-subtracted ChIP-Seq abundance at SVA repeats and their 3 kb flanking regions. All TE copies were scaled to the same length for visualization purposes. In the heatmaps, each row represents a SVA repeat of the indicated family and ordered by the corresponding H1 profile in each case (i.e. heatmaps show an independent order for each H1). Average profile of H1 variants per family is also shown in the upper line graphs. As this analysis is conducted from a group-level perspective, reads that mapped to multiple locations were taken into account. (**D**) Meta-repeat profile of H1X and ZNF91 input-subtracted ChIP-Seq abundance at SVA_F repeats and their 1 kb flanking regions. All TE copies were scaled to the same length for visualization purposes. SVA_F elements are divided in four groups considering whether are isolated (alone) within the genome, or flanked within 500 bp by other SVA elements in 5′ (last), 3′ (first), or 5′ and 3′ simultaneously (internal). (**E**) Heatmap and cluster analysis of the average input-subtracted ChIP-Seq abundance (scaled) of H1 variants (T47D cells) and other chromatin features (see Materials and methods) within SVA families. Average RNA-Seq, cheRNA-Seq, ATAC-Seq and MeDIP-Seq signal for each SVA family are included, annotated from high to low relative levels. (**F**) Boxplot of input-subtracted ChIP-Seq abundance of different features within the six SVA families, separated between non-expressed (*Ne*) and expressed (≥3 uniquely-mapped RNA-Seq reads; (E) SVAs. Basal RNA-Seq expression, as well as MeDIP-Seq abundances are also represented for the same groups. Statistically significant difference between not expressed (Ne) and expressed (E) SVA repeats was assessed using the Mann–Whitney *U* test (***) *P* < 0.001; (**) *P* < 0.01; (*) *P* < 0.05. (**G**) Meta-repeat profile of ZNF91, KAP1, H3K9me3 and DNA methylation input-subtracted ChIP-Seq abundance at SVA repeats and their 3kb flanking regions. All TE copies were scaled to the same length for visualization purposes. In the heatmaps, each row represents a SVA repeat of the indicated family and rows are ordered by the corresponding feature profile in each case. Average profile per family is also shown in the upper line graphs. Multi-mapping reads were also considered in this analysis. (**H**) Spearman's correlation coefficients between all the features analyzed in SVAs (All SVAs, SVA_A and SVA_F, respectively) using only uniquely mapped reads. Only correlations with *P*-value < 0.01 were considered (colored squares in the correlation matrices).

As shown in Figure 1C, H1X is highly enriched within the 6 SVA (SINE-VNTR-Alu) families (SVA_A to SVA_F), whereas H1.4 is relatively less abundant but still more than the other H1 variants. SVAs appeared along the evolution of great apes, being SVA_A the oldest one and SVA_E/F the youngest ones only present in humans (Supplementary Table S4) (5). SVAs have been further subcategorized with evolutionary criteria (61). In all cases, H1X was found progressively enriched from the oldest to the youngest SVA families, whereas H1.4 abundance was quite uniform across SVA groups (Figure 2A, B and Supplementary Figure S7A). On the other hand, low GC H1 variants are depleted from SVAs (Figure 1B, C and Supplementary Figure S6) but present different relative abundance patterns within SVA families.

Meta-repeat profiles for the different SVA families confirmed that H1X was more abundant at the recently-evolved SVA_F family, and even expanded to the 5′ and 3′ flanking regions (Figure 2C). Older SVA_A showed less H1X and more restricted to the repeat. Because SVA elements can be located in tandem or close one to each other in some cases, especially for SVA_F (data not shown), we explored whether the expansion of H1X to the flanks of SVA_F elements could be due to the presence of flanking SVAs. Whenever an additional SVA element was present upstream or downstream (or both) of an SVA_F element, H1X abundance was high (Figure 2D), confirming that the spread of H1X outside of the SVA_F elements in the meta-repeat analysis was due to the presence of flanking SVAs. Moreover, H1X tends to be enriched at the 5′ moiety of SVAs, especially when alone or at the end of a tandem (Figure 2D).

SVA elements are regulated by multiple mechanisms, including zinc finger proteins (KRAB-ZFPs). Concretely, ZNF91 is reported to bind SVAs and recruit KAP1 (TRIM28), which promotes a repressive state characterized by H3K9me3 (18,19). Thus, in order to explore a putative role of H1X in SVA elements, we next evaluated how H1X abundance correlates with histone marks and chromatin-associated factors (Figure 2E–H and Supplementary Figure S8A, B). Interestingly, H1X was the only factor gradually increasing towards the youngest SVAs, while the other factors were more enriched at the oldest SVAs (H3K9me3, H3K27me3, H4K20me3, DNA methylation, ZNF91, KAP1), or showed no significant difference among SVA families (Figure 2E-G and Supplementary Figure S8B). RNApolII and RNA-Seq signal also increased towards the youngest SVAs, suggesting that some of these elements might be transcribing (Figure 2E). Besides, the recently-evolved SVAs are more abundant within introns compared to the oldest SVAs, which are enriched at intergenic regions (Supplementary Figure S7B).

Compared to its genomic flanking regions, SVA elements showed a decrease on all histone marks, but enrichement on H1X/H1.4, ZNF91, KAP1 and DNA methylation (Figure 2G and Supplementary Figure S8B). ZNF91 is highly associated to SVA elements: out of 6732 regions of ZNF91 enrichment in the human genome, 42% are found within SVAs, and 78% of SVAs contain a ZNF91 peak (Supplementary Figure S8C). On the other hand, 83% of SVAs contain a H1X peak. The association between H1X, ZNF91 and KAP1 was further investigated, by computing the abundance of H1 variants around the meta representation of ZNF91 or KAP1 genomic enrichment regions. As expected H1X, and in some extent H1.4, were highly enriched within the majority of ZNF91 occupied regions. H1X and H1.4 were also highly enriched in ≈1/3 of KAP1 regions (Supplementary Figure S8D).

We also analyzed the location of ZNF91 and other factors within SVA_F tandems and compared to H1X. Interestingly, ZNF91 (and KAP1 to some extent) were enriched within SVA isolated or located at the ends of SVA tandems (external), but not at the internal SVA_F of a tandem (Figure 2D and Supplementary Figure S9). This distribution was more defined than the distribution of H1X. H1X was widely present along internal SVAs. Within external SVAs, H1X distribution started diffusedly at the middle of the first SVA of a tandem and ended suddenly at the middle of the last SVA, at regions occupied by ZNF91. To some extent, RNApolII followed H1X distribution. DNA methylation was more abundant at the 5′ end of isolated SVA_Fs or at the first SVA of a tandem, although few SVA_Fs showed methylation all along. Instead, at older SVAs (A, B and C), DNA methylation covered the whole element (Figure 2G). In conclusion, H1X presents a more extended distribution within SVA elements and tandems than other features such as ZNF91, KAP1 or DNA methylation, but similar to RNA polymerase. Both in isolated SVA_F or SVA tandems, ZNF91 is located at the 5′ and 3′ ends of these elements, flanking H1X enrichment (Supplementary Figure S9). These apparent inter-connected binding patterns could suggest that H1X, ZNF91 but also KAP1 and others, may cooperate in SVAs repression.

For the meta-repeat profiles, multi-mapping reads were used because copies of recently incorporated TEs (i.e. SVA_F) have not diverged enough to efficiently assign a unique position, affecting specially those H1 variants enriched within recent TEs (i.e. H1X/H1.4) (Supplementary Table S2 and Figure S9D, E). Despite of this, analogous results of H1X and ZNF91 profiles within tandem SVA_F were found when using uniquely-mapped reads only (Supplementary Figure S9C).

According to our RNA-Seq data, a similar percentage (≈10%) of SVA elements within each family were found expressed (≥3 uniquely mapped reads). We separated SVA elements on expressed and unexpressed and computed abundance of H1X and the other features (Figure 2F). Repressive marks (H3K9me3 and H3K27me3) were higher at non-expressed elements, whereas RNApolII, DNA methylation, ZNF91, KAP1 and H1X, to some extent, were higher at expressed elements (Figure 2F).

The analysis of correlations within individual SVA repeats showed that H1X abundance correlated positively with H1.4 and did not correlate with the other variants. H1.2 abundance correlated positively with H1.0, H1.3 and H1.5, but not with H1.4 or H1X. H1X (and H1.4) also correlated with ZNF91, DNA methylation and ATAC-Seq signal (Figure 2H and Supplementary Figure S10A). Instead, H1X showed a negative correlation with some histone marks such as H3K27me3, H3K27ac or H3K4me2, and ZNF93. H1.4 correlated with H3K9me3 and KAP1 better than H1X. H1.0/H1.2/H1.3/H1.5 positively correlated with H3K27me3 and some active marks, and negatively with ATAC-Seq signal. Some correlations among H1 variants and other features varied among SVA families (Figure 2H and Supplementary Figure S10B).

Altogether, results suggest that older SVAs have accumulated repressive factors and histone marks to a greater extent than recently incorporated SVA families. Those elements that are effectively repressed accumulate repressive histone marks (H3K9me3 and H3K27me3), whereas those that are still partially expressed tend to accumulate factors such as ZNF91 and KAP1 that might be participating in the attempts to repress them in a dynamic way. Within this picture, H1X is enriched at the younger SVAs which may still be in the process of being stably repressed.

### H1.4 and H1X are enriched at Alu SINE transposons recently incorporated

**Figure 3.**
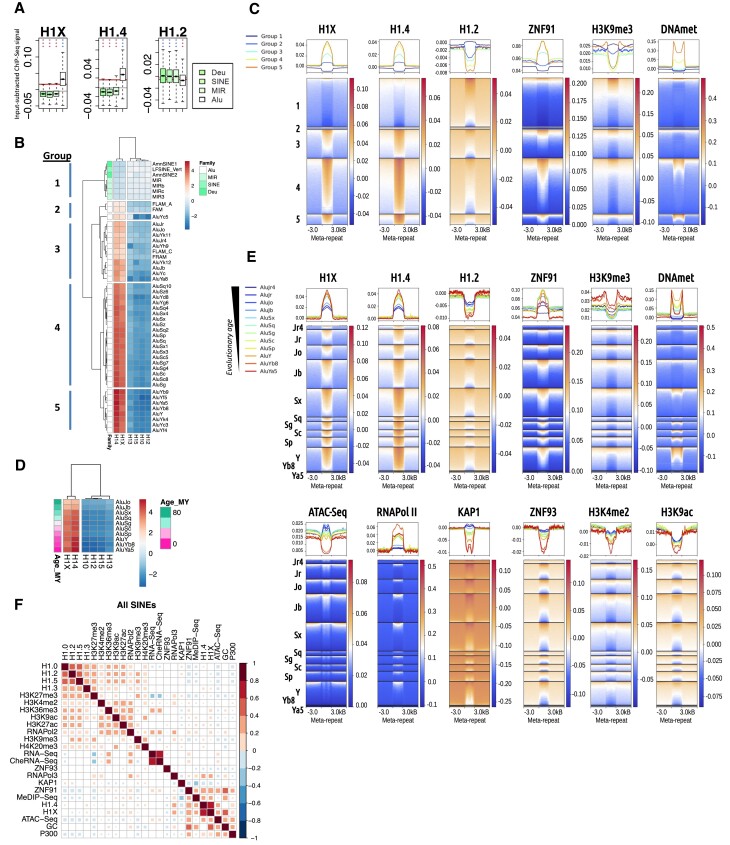
H1.4 and H1X are enriched at Alu SINE transposons recently incorporated. (**A**) Boxplot of H1 variants input-subtracted ChIP-Seq abundance within the SINE families (*N* = 4). In each boxplot, gray horizontal line corresponds to the median abundance of the corresponding H1 variant within all repetitive element classes (*N* = 9). Red horizontal line indicates median abundance of the corresponding H1 variant within all SINE repeats. Statistics were performed as in Figure 2A. (**B**) Heatmap and cluster analysis of the average input-subtracted ChIP-Seq abundance (scaled) of H1 variants (T47D cells) within the groups of SINE repeats (*N* = 48) belonging to the four families. Clustering generated five groups further used. (**C**) Meta-repeat profile of H1 variants (T47D cells) and other chromatin features input-subtracted ChIP-Seq abundance at SINE repeats divided in five groups according to (B) and their 3kb flanking regions. All TE copies were scaled to the same length for visualization purposes. In the heatmaps, each row represents a SINE repeat of the indicated group and ordered by the corresponding feature profile in each case. Average profile per group is also shown in the upper line graphs. Multi-mapping reads were considered in this analysis. (**D**) Heatmap and cluster analysis of the average input-subtracted ChIP-Seq abundance (scaled) of H1 variants (T47D cells) within selected groups of Alu elements (*N* = 10) ordered chronologically according to (58). All TE copies were scaled to the same length for visualization purposes. Multi-mapping reads were considered in this analysis. (**E**) Meta-repeat profile of H1 variants (T47D cells) and other chromatin features input-subtracted ChIP-Seq abundance at selected Alu repeats (*N* = 12) and their 3 kb flanking regions. (**F**) Spearman's correlation coefficients between all the features analyzed in SINEs using only uniquely mapped reads. Only correlations with *P*-value <0.01 were considered (colored squares in the correlation matrices).

SINE repeats are classified in four families: Alu, MIR, SINE and Deu. Alu is the most abundant family, occupying 10% of the genome, and contains 41 different repeat types (Supplementary Figure S3A), and was enriched in H1.4 and H1X (Figure 1C and 3A). A heatmap representation of H1 variants abundance within the 48 SINE repeat types generated 6 clusters of repeats, one of them including the MIR, SINE and Deu families, with the lowest amount of H1X and H1.4 and the highest of H1.2 (and the other variants, data not shown) (Figure 3B). The three clusters that were enriched in H1.4 and H1X, from high to low, were represented by Alu elements of the groups AluY, AluS and AluJ, respectively.

The 6 clusters of SINE repeats were pulled into five groups for further characterization. Group 1 contain non-Alu SINE (MIR, SINE and Deu families). Groups 3, 4 and 5 contain Alus J, AluS and AluY, respectively. This corresponds to Alu subfamilies incorporated along evolution from recent to older (4,58), meaning that H1.4/H1X are gradually enriched towards recently incorporated Alu elements (AluY subfamily). H1.4 was abundant throughout all repeats in groups 3–5 (Figure 3C). Other epigenetic features were more heterogeneous within each group, including H1.2, which was enriched only in a subset of repeats (Figure 3C and Supplementary Figure S11A). Within group 1, there are some repeats that are expressed and showed a similar chromatin accessibility than their flanking regions. Instead, in group 3–5 some repeats that are located within open and expressed regions, are clearly locally repressed (Supplementary Figure S11A). Interestingly, group 3 repeats are enriched in H1.4/H1X but present low DNA methylation, whereas groups 4 and 5 share both features (Figure 3C).

To further confirm the observed association between H1 variants abundance and Alu elements evolution, we used an alternative selection of *N* = 12 Alu subgroups, as analyzed by Su *et al.* (21), sorted by their evolutionary age (according to Giordano et al, (58)). This confirmed that H1.4 and H1X are enriched gradually at the recently incorporated Alu (Figure 3D), as previously shown using H1.4/H1X peaks (Supplementary Figure S5B, C). In addition, the heatmap showed that all repeat members of each type contain these H1 variants highly enriched compared to flanking regions, and the other variants are deprived (Figure 3E). In parallel, H3K9me3 was slightly increased at recent Alu, although heterogeneous within each repeat type. DNA methylation was also increased at recent Alu, although the two youngest elements, AluYb8 and AluYa5, presented less methylation than the next AluY (Supplementary Figure S12A).

We also explored the abundance of KAP1, ZNF93 and ZNF91 within these Alu elements to check whether these proteins could be involved in the repression mechanism. KAP1 and ZNF93 abundance was heterogeneous among repeats of each Alu type and were decreased at recent Alu. ZNF91 was also heterogeneous, although it increased at recent Alu (Figure 3E). Active epigenetic marks were also analyzed. H3K4me2 and H3K9ac were low at Alu compared to flanking regions, although heterogeneous among repeats of the same type, and decreased at recent Alu. Additionally, ATAC-Seq signal decreased at recent Alu, especially at the two youngest elements. The ATAC profile was compatible with nucleosome positioning.

Evolutionary recent Alu elements are reported to hold two well-positioned nucleosomes (59). Related to this, the central enrichment of H1.4 and H1X at these young Alus suggest that these positioned nucleosomes were loaded with these histone variants. In fact, profiling of MNase-seq data around H1X/H1.4 peaks, which accumulate within young Alu (Figure 1E, Supplementary Figure S5), confirmed the coincidence of these variants with positioned nucleosomes (Supplementary Figure S11B, C).

Then, we separated Alu elements depending on their basal expression (expressed or unexpressed) and computed abundance of H1X and the other features (Supplementary Figure S12A). KAP1 and repressive marks (H3K9me3 and H3K27me3) were higher at non-expressed elements, whereas RNApolII, DNA methylation, ZNF91 and H1X, to some extent, were higher at expressed elements. As shown above, H1X, DNA methylation and ZNF91 increased towards the most recent Alu elements until AluY.

The analysis of correlations between all features at SINEs (total or separated on groups 1–5) showed that H1.0/H1.2/H1.3/H1.5 correlate among them (although H1.3 was more divergent from the other variants), and also with H3K27me3 and in some extent with H3K9me3 (groups 1–3), but also with some active marks (Figure 3F and Supplementary Figure S12B, C). H1.4/H1X showed some slight correlation with ATAC-Seq (groups 3–5), and with ZNF91, DNA methylation and RNApolIII (only when all SINEs were considered together). Within group 1, H1.4 (and less H1X) also showed some correlation with H1.0/H1.2/H1.3/H1.5, H3K27me3 and RNApolIII.

In conclusion, our data suggests that H1.4 and H1X may be involved in the repression of Alu elements, in particular those that were incorporated more recently within the genome, in conjunction with DNA methylation and strong nucleosome positioning.

### H1.4 and H1X are enriched within a small subset of L1 LINE transposons

**Figure 4.**
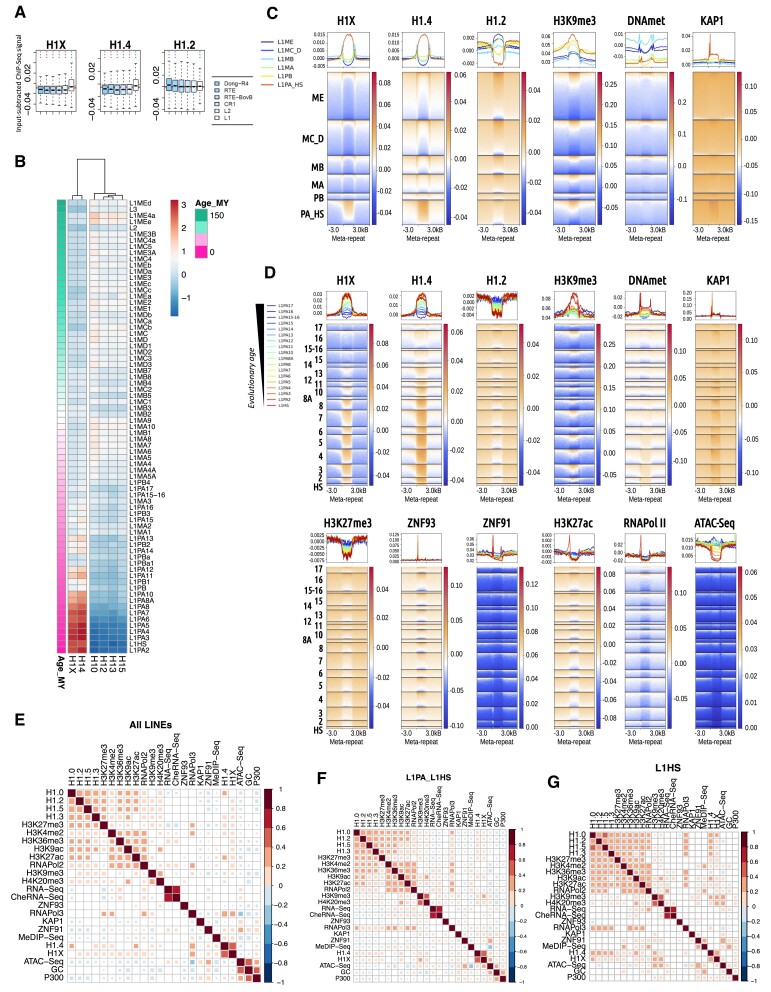
H1.4 and H1X are enriched within a subset of L1 LINE transposons. (**A**) Boxplot of H1 variants Input-subtracted ChIP-Seq abundance within the LINE families (*N* = 6). In each boxplot, gray horizontal line corresponds to the median abundance of the corresponding H1 variant within all repetitive element classes (*N* = 9). Red horizontal line indicates median abundance of the corresponding H1 variant within all LINE repeats. Statistics were performed as in Figure 2A. (**B**) Heatmap and cluster analysis of the average input-subtracted ChIP-Seq abundance (scaled) of H1 variants (T47D cells) within selected groups of LINE elements (*N* = 73) ordered chronologically according to (58). (**C**) Meta-repeat profile of H1 variants (T47D cells) and other chromatin features input-subtracted ChIP-Seq abundance at L1 LINE repeats divided in 6 subfamilies and their 3kb flanking regions. All TE copies were scaled to the same length for visualization purposes. Multi-mapping reads were also considered in this analysis. (**D**) Meta-repeat profile of H1 variants (T47D cells) and other chromatin features input-subtracted ChIP-Seq abundance at L1PA_HS elements (*N* = 18, ordered chronologically) and their 3kb flanking regions. In the heatmaps, each row represents a LINE repeat of the indicated group and ordered by the corresponding feature profile in each case. Average profile per group is also shown in the upper line graphs. Multi-mapping reads were also considered in this analysis. (E–G) Spearman's correlation coefficients between all the features analyzed in LINEs (**E**), L1PA_L1HS subfamily (**F**) or L1HS elements (**G**), using only uniquely mapped reads. Only correlations with *P*-value <0.01 were considered (colored squares in the correlation matrices).

LINE repeats are classified in 6 families: L1, L2, CR1, RTE, RTE-BovB and Dong-R4, being L1 and L2 the most abundant occupying 17 and 3.5% of the genome, respectively (Supplementary Figure S3A). L1 family contains 122 repeat types that can be further divided in six groups/subfamilies: L1ME, L1MC_D, L1MB, L1MA, L1PB and L1PA_HS. Although H1.0/H1.2/H1.3/H1.5 are enriched in all LINE families (Figures 1C, 4A), a deeper analysis showed variable H1 variants abundance among LINE repeats (Supplementary Figure S13). Concretely, L1 family showed H1.4/H1X enrichment, especially in L1PA elements. Moreover, low GC H1 variants exhibited specific relative enrichments within particular LINEs.

Human L1 elements can be traced back in the evolution of mammals and can be ordered chronologically as million years since they appeared, for example by genome defragmentation (58). L1 subfamilies, from older to recent, are ordered as L1ME, L1MC/D, L1MB, L1MA, L1PB and L1PA_HS, with overlap among L1 members. We have represented the abundance of each H1 variant within L1 elements ordered chronologically and found that H1X/H1.4 are gradually enriched towards the youngest L1 elements, parallel to the decrease of the other variants (Figure 4B). H1X/H1.4 are highly enriched at the most recent L1 elements of the L1PA subfamily. This was also seen using H1.4 and H1X peaks (Supplementary Figure S5D).

Meta-repeat analysis of L1 subfamilies and of the L1PA_HS subfamily repeat members was also performed (Figure 4C, D and Supplementary Figure S14A–C). Among the L1 subfamilies, L1PA_HS showed enrichment of H1.4 and H1X, H3K9me3 and KAP1, and depletion of H3K27me3, active histone marks and RNApolII. DNA methylation was present in some of the repeats of the oldest L1 subfamilies (L1ME, L1MC/D, L1MB). Interestingly, some of the genomic regions flanking the oldest L1 elements were characterized by open chromatin features (ATAC accessibility, RNA-Seq reads, H3K27ac, H3K4me2), whereas genomic regions flanking the youngest L1 subfamilies were slightly enriched in H3K9me3, K3K27me3, KAP1 and H1 variants such as H1.2, compared to the oldest L1s (Figure 4C and Supplementary Figure S14A). Altogether, data indicate that the oldest L1 elements are more repressed than their flanking genomic regions with putative participation of DNA methylation, repressive histone marks and H1 variants such as H1.2, whereas the youngest L1 elements are immersed in already repressive environments and may be further repressed by H3K9me3, KAP1 and H1.4/H1X. Indeed, when filtering L1 subfamilies based on their H3K9me3 abundance (top 10% or bottom 10% of H3K9me3 abundance), we confirmed the co-occurrence of this histone mark and H1 variants within L1 repeats. H1X/H1.4 were highly enriched in those L1PA_HS with higher H3K9me3 levels while low GC variants were enriched in top-marked H3K9me3 oldest L1 subfamilies (Supplementary Figure S14B).

Looking into detail within the L1PA_HS subfamily, H1.4 and H1X were progressively enriched towards the most recent elements, in parallel to H3K9me3 accumulation, DNA methylation and ATAC-Seq decreased accessibility (Figure 4D and Supplementary Figure S14C). H1.4 and H1X were present all along the L1 element. Instead, KAP1, ZNF93, ZNF91 and some active marks such as H3K27ac (and RNApolII) accumulated at the 5′ end of the most recent repeats. Interestingly, ZNF93 and KAP1 were enriched in L1PA3 and L1PA4, but not in L1PA2 (and L1HS) (18), which presented some derepression and RNApolII, H3K27ac, ZNF91 and DNA methylation enrichment at 5′, suggesting that the most recent L1 elements are not fully repressed yet.

Classifying L1PA_HS repeats based on their basal expression (expressed/unexpressed), we observed that only H3K27me3 was higher at non-expressed elements, whereas H1X, ZNF91, KAP1, DNA methylation and H3K9me3 displayed higher levels at some of the most recent, expressed elements (Supplementary Figure S15A). As shown above, H1X, ZNF91, KAP1, DNA methylation and H3K9me3 increased towards the most recent L1PA_HS elements, in parallel to decreased expression and RNApolII presence (Supplementary Figure S15A).

The analysis of correlations between all features at L1 LINEs (total or separated on the six L1 groups) showed that H1.0/H1.2/H1.3/H1.5 slightly correlate among them, and also with H3K27me3 and some active marks, and with RNApolIII in the oldest L1 groups (Figure 4E, F and Supplementary Figure S15B). H1.4/H1X strongly correlated among them and showed poor correlations with other features (RNApolIII, ZNF91, H3K36me3). Correlations involving H1.4/H1X were the ones that varied the most among L1 groups (Supplementary Figure S15C). In L1PA_HS, H1X (and H1.4) acquired a positive correlation with H3K9me3 (and H4K20me3), especially at the most recent elements (L1PA8A to L1HS). In parallel, correlation between H1X and H1.4 decreased at the most recent L1PA_HS elements (Figure 4F, G and Supplementary Figure S15D).

In conclusion, we have seen H1X and H1.4 accumulation at the most recent L1PA_HS subfamily elements, where it might participate in its repression together with KAP1, H3K9me3 or DNA methylation. Contrary to these features, H1X and H1.4 do not accumulate only at the 5′ end of these L1 elements, but occupy the whole element, without expanding to flanking genomic regions.

### H1X and H1.4 are enriched within transposable elements recently incorporated into the genome along primates evolution including LTR repeats

**Figure 5.**
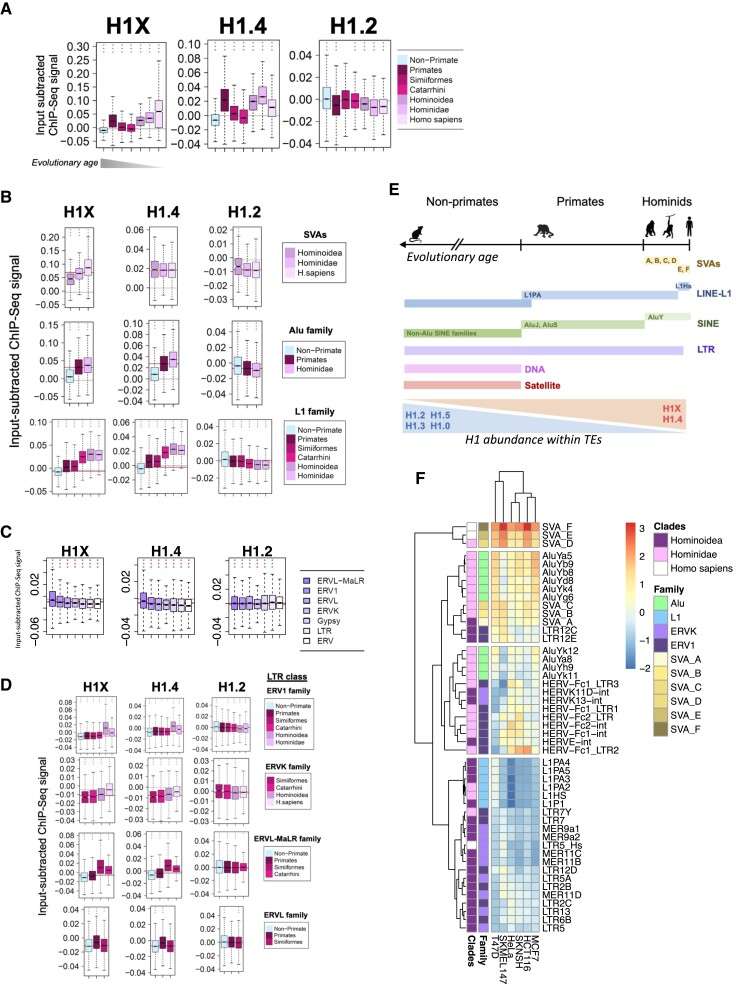
H1X and H1.4 are enriched within transposable elements recently incorporated in the genome along primates evolution including LTR repeats. (**A**) Boxplot analysis of H1 variants input-subtracted ChIP-Seq abundance (T47D cells) within repetitive elements classified into different taxonomic clades according to Dfam database, ordered by their evolutionary age. The classification of repeats corresponds to the taxonomic clade to which the oldest ancestor in which the repeat has been found, belongs (see Materials and methods). (**B**) Boxplots indicate the H1 variants input-subtracted ChIP-Seq abundance within repeats of Other Class (i.e. SVA families), SINE-Alu family and LINE-L1 family, classified according to taxonomic clades. (**C**) Boxplot of H1 variants input-subtracted ChIP-Seq abundance within the LTR families (*N* = 7). In each boxplot, gray horizontal line corresponds to the median abundance of the corresponding H1 variant within all repeats. (**D**) Boxplots indicate the H1 variants input-subtracted ChIP-Seq abundance within repeats of LTR families ERV1, ERVK, ERVL-MaLR and ERVL, classified according to taxonomic clades. LTR families ERV, Gypsy and LTR are not included because all repeats within these families are classified as Non-primate. (**A–D**) In each boxplot, gray horizontal line corresponds to the median abundance of the corresponding H1 variant within all repeats. Red horizontal line indicates median abundance of the corresponding H1 variant within all repeats from the family or class being evaluated. One-sample Wilcoxon signed-rank test was used to statistically support H1 enrichment (blue asterisks) or depletion (red asterisks) compared to the median H1 variant abundance within all repeats (A) or compared to the median H1 variant abundance within repeats from the evaluated family or class in each case (B, D). (***) *P*-value <0.001; (**) *P*-value <0.01; (*) *P*-value <0.05. (**E**) Timeline illustrating the human genome evolution and the time lapses in which repetitive elements classes or families were approximately incorporated into the host genome. In general, H1.4 and H1X abundance increase at recently-incorporated repeats along primates evolution, including SVAs, Alu but also LINE-L1 and LTR repeats, while H1.2/H1.3/H1.5/H1.0 are enriched in older elements. Illustrations refer to the H1 variants distribution in T47D cells. Of note, in other cancer cell lines H1X is also enriched within SVAs and SINE classes and gradually increases towards more recent SVA and Alu families/subfamilies. **F**) Heatmap and clustering of H1X input-subtracted ChIP-Seq median abundance (scaled) in six different cancer cell lines (T47D, SKMEL147, HeLa, SKNSH, HCT116, MCF7) at *N* = 48 repeats from *Hominoidea* and descendant clades. Y-axis annotation indicates repetitive element family and taxonomic clades.

Among all epigenetic features analyzed within SVA, Alu and L1 elements in the previous sections, it is striking that H1X and H1.4 are the ones that better correlate with the evolutionary age of the studied TEs. To delve deeper into this relationship, the DFam database (56) was utilized to classify TEs based on their evolutionary age, according to the oldest taxonomic clade or species in which copies have been found. We categorized TEs into distinct taxonomic clades (*N* = 8), ranging from Non-primates to *Homo sapiens* (see Supplementary Table S4). Boxplots were generated to depict the content of H1 variants within repeats for each clade, revealing that H1X and H1.4 were more enriched in primate-restricted repeats compared to those also found in non-primates (Figure 5A). Additionally, H1X gradually increased in abundance toward human-specific repeats, while H1.2 exhibited the opposite pattern.

To explore the relationship between H1 variants abundance and the evolutionary age of TEs, we first focused on previously studied SVA, Alu and LINE-L1 repeats (Figure 5B). In the case of SVA retrotransposons, H1X abundance exhibited a gradual increase along the evolutionary lineage, while H1.4 remained relatively constant. Regarding Alu family, both variants gradually increased towards more recent clades but H1.4 showed a higher relative enrichment in more evolutionarily recent Alu elements found in hominids compared to H1X. Similarly, for LINE-L1 repeats, H1X and H1.4 abundances increased along the evolutionary timeline, while H1.2 was more abundant in L1 repeats also found in non-primate species.

Furthermore, the analysis of LTR repeats revealed similar H1 variants binding patterns along evolution as seen for other TE families. Although H1X and H1.4 are in general depleted from LTR (Figures 1B, 5C), their abundance gradually increases towards more recent LTR repeats from ERV1, ERVK and ERVL-MaLR, while H1.2 abundance decreased in more recent LTR repeats (Figure 5D).

Thus, analysis of repeat families revealed that H1X and H1.4 exhibited the highest abundances in Hominoidea/Hominidae/H.sapiens restricted repeats compared to older members of the same family (Figure 5A ,B, D). Heatmap analysis of repeats restricted to Hominoidea and descendant clades (*N* = 48, see Supplementary Table S5) confirmed that the highest H1X abundance was found within SVA elements, followed by AluY repeats and young LINE-L1 elements (Supplementary Figure S16). In contrast, H1.4 showed the highest abundance in recently evolved AluY repeats and young LINE-L1 elements, surpassing its enrichment in SVA families. Notably, both H1X and H1.4 were more enriched in recent LINE-L1 compared to recent LTR repeats. Interestingly, H1X was relatively enriched within LTR12C/E elements from ERV1 family (Supplementary Figures S16 and S5E).

Overall, evolutionary classification of TEs allowed us to confirm the accumulation of H1X and H1.4 at more recent elements, especially along the SVA and Alu lineage. In addition, although both LINE and LTR classes are enriched in low GC H1 variants abundance compared to H1X/H1.4, clades classification analysis of LINE and LTR families showed that H1X and H1.4 are enriched within a small subset of LINE-L1 and LTR repeats. These subsets coincided with the elements most recently incorporated in our genome. On the other hand, H1.2, H1.3, H1.5 are more abundant at more ancient TEs of the afore-mentioned classes/families (summarized in Figure 5E).

To investigate whether our findings can be extrapolated to other cell types, we performed H1X ChIP-Seq in multiple cancer cell lines. We found that H1X was consistently enriched in SVA and SINE classes (Supplementary Figure S17A). This H1X enrichment was also evident when focusing on SVA families and young Alu repeats (Figure 5F), similar to T47D cells. H1X profiling within SVA families and Alu subgroups confirmed the universal H1X enrichment towards more recent elements (Supplementary Figure S17B). Indeed, H1X abundance gradually increased towards evolutionary recent TEs when clades classification was considered (Supplementary Figure 17C). This taxonomic classification showed that H1X abundance increased along evolution of SVA and Alu lineage in all cell lines evaluated (Supplementary Figure S17D). However, relative enrichment of H1X in LINEs and LTRs varied among cell lines. Only in SK-MEL-147 cells H1X was enriched at recent LTR and L1, similar to T47D (data not shown). Despite this cell-line-specific binding patterns in LTR repeats, heatmap analysis of Hominoidea repeats showed that H1X is universally enriched in LTR12C/E elements (Figure 5F). This was further confirmed by profiling H1X abundance within LTR12C/E (Supplementary Figure S17E).

Finally, we confirmed by ChIP-qPCR that H1X was enriched in recent TEs such as SVA_F and AluY compared to older elements (satellites and non-primate LTRs), and the comparative ratio between H1X and H1.2 abundance was always higher for these recent elements (Supplementary Figure S18).

In summary, this analysis highlights the correlation between H1X abundance and TEs evolutionary age. Our results suggest that H1X enrichment within more recently-incorporated SVA and Alu elements is universal among different cancer cell lines.

### TE expression changes upon H1X depletion

Overall we have shown that H1X is highly enriched within SVA and Alu transposable elements along different cell lines, and also at the most recently incorporated L1 and LTR elements in some cell lines. In such situations, H1X and H1.4 coincide with repressive epigenetic marks such as H3K9me3, DNA methylation or members of the repressor complex KRAB-ZNF/KAP1. To elucidate whether H1X exerts a repressive role in silencing young TEs, we used a previously developed H1X inducible shRNA system in T47D (Supplementary Figure S19A) (28). Gene expression changes were originally studied with a microarray platform (28), but here, we performed RNA-Seq experiments to analyze changes in TEs expression upon H1X KD.

**Figure 6.**
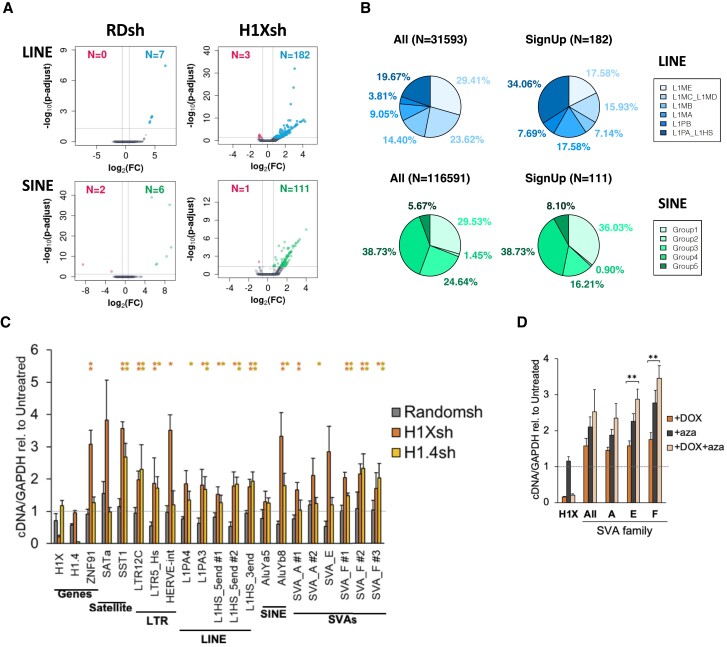
TE expression changes upon H1X depletion. (**A**) Volcano plots of TEs expression changes upon H1X KD or Random shRNA control in T47D cells using uniquely mapped RNA-Seq reads. Three biological replicates were performed for each condition. Up and down-regulated LINE-L1 or SINE elements are colored in blue/green or red, respectively. Repeats with a |FC|>1.5 and a *P*-adjust < 0.05 were considered as significantly deregulated. (**B**) Pie-chart showing the proportion of up-regulated LINE or SINE belonging to each group indicated (previously described in Figures 3 and 4). For comparison, the proportion of total LINE and SINE elements that met the criteria for this analysis within each group is shown. (**C**) RT-qPCR performed in T47D H1X or H1.4 KD denoted upregulation of multiple repetitive elements. T47D Randomsh were also analyzed as control. Apart from SATa and SST1 satellites, the rest of repeats analyzed are restricted to Hominoidea species (see Supplementary Table S5) and enriched in H1X and H1.4, as denoted by ChIP-Seq analyses. Hash (#) indicates different primer pairs. H1X and H1.4 expression was measured to show H1 KD efficacy. ZNF91 gene was up-regulated upon H1X KD. Expression was corrected by GAPDH and expressed relative to Untreated condition in each case. For each KD, statistical significance of upregulation upon + Dox treatment compared to untreated is supported by *t*-test. (*) *P*-value < 0.1; (**) *P*-value < 0.05. (**D**) H1X depletion and Aza treatment have an additive effect in activating young TEs. Combined Dox (H1X KD induction) and Aza (DNMT inhibitor) treatment was performed and expression of SVA was analyzed by RT-qPCR with different oligonucleotide pairs. Expression was corrected by GAPDH and expressed relative to Untreated condition. H1X expression was measured to show H1X KD efficacy. Statistical differences between +Dox and +Dox + aza are supported by *t*-test. (**) *P*-value < 0.05.

Changes on individual repeats were analyzed using uniquely mapped RNA-Seq reads upon H1X KD, compared to Random shRNA expression. Volcano plots were generated for the different TE classes were H1X enrichment was detected (Figure 6A and Supplementary Figure S19B). SINE and LINE repeats showed a larger number of up-regulated elements than down-regulated, supportive of a repressive role for this H1 variant. This was not as important for H1.4 or H1.2 KD, for which RNA-Seq data was also available (31,32) (Supplementary Figure S19B). The identity of up-regulated elements upon H1X KD was investigated. For L1 LINEs, the most recent groups were enriched within the up-regulated elements compared to the expected frequency (34.1% of up-regulated elements belong to L1PA_HS group, expected was 19.7%) (Figure 6B). For SINEs, Group 5 containing AluY elements was slightly enriched above the expected frequency (8.1% versus 5.7%), but no big differences existed for the other groups compared to their total abundance (Figure 6B).

Because SVA elements did not show much of up-regulation using this analysis method that is quite restrictive (Supplementary Figure S19B), we looked in detail at which elements showed changes in RNA-Seq reads abundance upon H1X KD. Some of the elements were not expressed before and after H1X KD (23.4%) (Supplementary Figure S19C). A significant number of elements became expressed or increased the number of reads upon H1X KD, 7.9 and 28.2%, respectively, whereas 8.4 + 29.8% decreased the number of reads. Up-regulated elements were enriched in the most recent SVA_D to F elements compared to the frequency of each family in the total count of SVA elements (Supplementary Figure S19D).

Expression changes of recently incorporated TEs upon H1 depletion were further examined using specific primers designed for these repeats in RT-qPCR analysis. In particular, a mild upregulation of SVAs, AluY elements, L1 from the PA_HS subfamily and LTR12C repeats was found upon H1X KD (Figure 6C). Some Satellite repeats were also up-regulated. H1.4 KD caused a similar or lower upregulation of these TEs.

Because H1X could interact with DNA methylation for repression of TEs such as SVA, we tested the effect of H1X KD and azacytidine (Aza)-mediated inhibition of DNA methylation on SVA expression by RT-qPCR. Aza treatment induced SVA elements similar or better than H1X KD, and the two treatments were additive (Figure 6D). A similar effect was observed on L1, Alu and LTR elements tested (Supplementary Figure S19E). SATa satellite was more responsive to Aza than to H1X KD.

Among the genes up-regulated in H1X KD, we found ZNF91 (FC = 1.47 in RNA-Seq; FC ≈ 3 in RT-qPCR, Figure 6C), the KRAB-ZNF protein highly specific for SVA elements responsible for recruiting KAP1/SETDB1 for repression of these elements (18). Additionally, 15 more ZNF genes were found up-regulated with a FC ≥ 1.2 (data not shown). It has been described that SVA elements can regulate neighbor ZNF genes, which in turn participate in the regulation of SVAs establishing a feedback loop (19). Upon H1X KD, SVA elements could be deregulated, which in turn could cause the up-regulation of ZNF91. This could explain why the up-regulation of SVA elements detected upon H1X KD in our experiments was limited.

In conclusion, we have shown evidence that histone H1X (and H1.4) accumulate in transposable elements that have incorporated recently in the human genome in evolutionary terms, where they may contribute to its silencing to avoid deleterious effects including transposition and altering host gene expression. Future investigation of the cross-talk between these histone variants and other epigenetic features and repressor complexes involved in the silencing will help to clarify the molecular function of these variants, as well as how the specificity between H1 variants is established for its recruitment.

## Discussion

Our understanding of H1 variants heterogeneity has been very limited due to the lack of specific ChIP-grade antibodies. Recently, we overcame this limitation and performed the first genome-wide mapping of five endogenous H1 variants in T47D breast cancer cells (31). We determined that H1.0, H1.2 and H1.5 are more enriched in low GC genomic regions while H1.4 and H1X are more abundant within high GC regions. On the other hand, although a compromised H1 content has been associated with heterochromatin dysregulation and aberrant expression of repetitive elements (32,37), differential binding of H1 variants to repetitive elements classes has not been extensively explored. In this study, we have characterized the differential profiling of six H1 variants in T47D cells, which importantly represents the whole somatic H1 repertoire in these cells, with a focus in repetitive and transposable elements. Of note, H1.3 was added in the study, being the first genome-wide mapping of endogenous H1.3 within a mammalian cell. H1.3 has been found enriched at low GC and B compartment regions of the genome, similar to H1.0/H1.2/H1.5.

Evaluation of H1 variants abundance denotes an antagonistic distribution within repetitive elements classes. H1.4 and H1X are enriched within SVAs and SINE classes, while H1.2, H1.3, H1.5 and H1.0 are more abundant within LINE, LTR, Satellite or DNA classes. Although both H1X and H1.4 are enriched within SVAs and Alu, H1X is more associated to SVAs while H1.4 is preferentially bound to Alu elements. H1 variants levels are variable between families and repeats from the same class. Indeed, we have shown an unprecedented relationship between H1 variants abundance and evolutionary age of TEs (Figure 5A, B). Thus, in T47D, H1X and H1.4 are enriched towards most recent TEs of the human genome, including SVAs, Alu, but also at youngest repeats of LINE-L1 and LTR classes/families. On the other hand, H1.2/H1.3/H1.5/H1.0 are more enriched at oldest repeats. These results support H1 variants differential genome-wide distribution not only within large genomic regions or A/B compartments (30,31), but also within concrete subsets of repeats.

Study of tandem repeats suppose an additional challenge for short-read sequencing. In the hg19 genome assembly, almost 50% of Satellite repeats are located within problematic regions where read mapping is inaccurate. Considering this, all H1 variants are somehow abundant within satellite class but, compared to other repeat classes, enrichment is higher for low GC H1 variants. In agreement, simultaneous depletion of H1.2 and H1.4 strongly derepresses satellite repeats, including SATa and SST1 (32) pericentromeric satellite, which are also upregulated upon H1X depletion (Figure 6C). This highlights that different H1 variants may be involved in satellite repeats regulation. Importantly, mapping limitations could be overcome by long-read sequencing experiments, which have been recently found useful to map satellite repeats and to study the epigenetic state of repetitive elements (62).

Hominid-specific SVA retrotransposons are in general enriched with H1X and H1.4 and depleted for H1.2/H1.3/H1.5/H1.0 binding. However, different H1 variants show different abundance patterns when evaluating SVA families. We have found a gradual increase of H1X from older SVA_A to human-specific SVA_F families, while H1.4 abundance remains constant across SVA families. On the other hand, H1.2/H1.5/H1.0 abundances gradually decrease while decreasing evolutionary age of the families, whereas H1.3 abundance is homogeneous. Because H1X correlated with ZNF91, DNA methylation and ATAC-Seq signal in all SVA families (Figure 2H), it suggests that it may participate in the early repression of recently incorporated TEs. Recently incorporated repeats tend to be located within more active and accessible regions. Thus, our observations could reflect that those SVAs incorporated into regions that allow their expression are also enriched in repressive factors such as ZNF91 and DNA methylation that cooperate to silence them, probably in conjunction with H1X. In fact, H1X depletion triggers a moderate transcriptional activation of SVA families but also other young TEs from different families/classes. In addition, we have detected an additive effect between H1X and DNA methylation in TEs deregulation. These results are in concordance with a previous report showing that histone H1 and DNA methylation cooperatively silence transposons in plants (38). They also suggest an existent interplay between DNA methylation and H1 variants in TE silencing. Interestingly, H1X depletion (but not H1.4 KD) leads to ZNF91 upregulation (Figure 6C), which is a master repressor of SVAs (18,19). Moreover, derepression of SVAs is reported to cause upregulation of neighboring ZNF genes (19). It is plausible to propose that H1X can be involved in this feedback loop regulation. Hypothetically, SVA derepression mediated by H1X depletion could incite upregulation of ZNF91 to control SVA activation. This scenario could explain why just mild upregulation of SVAs is shown in H1X KD. Cooperation between H1X and ZNF91 in regulating SVA elements can be also supported by the correlation of their presence in SVAs (Figure 2H). We also have performed a deeper analysis of the SVA_F family and found that H1X and ZNF91 present characteristic profiles. SVA_F repeats tend to be located in tandem. Either isolated or clustered, while H1X presents a more extended distribution within SVA repeats, ZNF91 is located at the 5′ and 3′ ends of these elements, precisely flanking H1X enrichment (Figure 2D). KAP1 also follows ZNF91 profile. These apparently interconnected binding profiles may suggest that H1X and ZNF91/KAP1 form a repressive environment to control SVA expression, delimited by ZNF91 presence.

LINE and SINE are the predominant TE classes in the human genome. Alu family (SINE class) have successfully expanded through the primate genomes, representing up to ≈11% of the human genome. Along Alu evolution, elements show a gradual increase of both H1.4 and H1X accompanied by a gradual decreased of the rest of the variants. Indeed, clustering of SINE repeats according to H1 variants abundance results in five different groups with decreasing evolutionary ages (Figure 3D, E). While Group 1 includes SINE non-Alu families, Groups 2–5 are mostly represented by subsequent Alu subfamilies from older to more recent, being Group 5 mostly formed by the youngest AluY subfamily. It is worth mentioning that H1.4 is the feature that more consistently marks Groups 3–5 of Alu repeats, among all epigenetic marks evaluated, followed by H1X. Regarding LINEs, H1.2/H1.3/H1.5/H1.0 are abundant within all LINE families but, interestingly, H1X and H1.4 are enriched within a small subset of LINE-L1 repeats, which coincide with the most recent L1 elements from the L1PA_Hs lineage (Figure 4D). Moreover, data suggest that H1X participates in repression of these young L1 together with H3K9me3, as the same repeats that accumulated H1X, were enriched in H3K9me3. We also found that these recent L1 (L1PA_Hs) and Alu (Group 5) subfamilies were more upregulated upon H1X depletion, with respect to older elements of their lineages and considering their relative genomic occupancies (Figure 6B), pointing to a role of H1X in their transcriptional regulation.

In general, our analyses show that within TEs, H1 variants specifically coincide with other repressive factors and histone marks in a class and evolutionary age dependent manner. In fact, it has been described that repeats can be regulated by different epigenetic mechanisms depending on their evolutionary age. For instance, while young human LTRs tend to be repressed by DNA methylation, intermediate age LTRs are preferentially silenced by repressive histone modifications, including H3K9me3. The evolutionarily old LTRs are kept silenced by the accumulation of loss-of-function genetic mutations (12). This ‘epigenetic switch’ between DNA methylation and repressive histone marks is also reported during development (63) or cancer (64). Our results suggest that an analogous ‘H1 variants switch’ occurred within TEs during host genome evolution. On the other hand, although H1 variants levels are known to vary along differentiation and disease (65,66), their comparative genome-wide mapping in different developmental stages or along cancer progression, remains unexplored.

Strong nucleosome positioning has been postulated as another layer of transcriptional repression, masking the access to the transcription machinery. Due to the fact of mapping linker histones and considering the short length of H1X/H1.4 narrow peaks (Supplementary Figure S4B), H1 peak calling is intrinsically biased by nucleosome positioning. It has been shown that ≈ 80% of strong nucleosomes within the genome overlap with repeats, especially SINE/Alu and LINE/L1, with strong enrichment for evolutionary young Alu and L1 elements (59). Accordingly, >90% of H1.4 or H1X narrow peaks rely on repetitive elements (Figure 1D) and they are biased towards more recent repeats of the Alu and L1 families but also SVA retrotransposons (Supplementary Figure S5A–E). Therefore, our results show that these strong nucleosomes are in addition loaded with H1.4 and/or H1X and this linker histone presence could contribute to the cooperative silencing of TEs. Accordingly, MNAse-seq profiles around H1 peaks are compatible with strong-positioned nucleosomes (Supplementary Figure S11B). On the contrary, strong nucleosomes are underrepresented in older LINE/SINE elements, which implies nucleosome positioning changes during evolution, probably originated from the accumulation of mutations (59). Accordingly, H1.2/H1.3/H1.5/H1.0 are enriched within older TEs and peaks cannot be computed efficiently for these variants, suggesting their depletion from strong nucleosomes.

It is remarkable that H1 variants, specially H1X, are the epigenetic features that better correlate with the evolutionary age of TEs in different classes. Throughout evolution, waves of retrotransposon insertions have invaded mammalian genomes. For each invasion, the host genome finds a repressive mechanism to prevent the retrotransposon transcription. A clear example relies on the evolutionary coincidence between expansion of KRAB-KZNF gene family and TE insertions (67–69). However, this so-called ‘arms race model’ (18) does not fully explain the co-option between inserted TEs and the host genome and their integration in the regulatory networks (70). In the case of H1 proteins, H1 subtypes divergence occur prior mammalian radiation. That is, the seven somatic H1 variants present in humans are also present in other mammal species (71). For that reason, H1.4 or H1X proteins did not represent an example of ‘arm race mechanism’, as the proteins did not emerge themselves to repress primate-invading Alu elements or hominid-specific SVAs. Although these H1 variants were already functional in the ancestral genomes, TEs invasion could modulate their functionality or genomic distribution in a lineage or species-specific manner. Indeed, this scenario may also occur with KZNF proteins, as there are many examples of TEs being silenced by KZNF proteins emerged before their invasion (70). Hence, H1 variants profiling in other genomic backgrounds, including non-human primates and mammals non-primates, is potentially interesting to shed light into the interplay between functional adaptation of H1 variants and TE regulation through genome evolution. Related to this, in mouse embryonic stem cells (mESCs), ChIP-Seq of tagged H1.0 and knock-in of H1c and H1d (mouse orthologous of H1.0, H1.2 and H1.3) have been performed (39). Regarding repetitive elements, the three H1 variants were found enriched within satellites and LINEs. Those results somehow could be in concordance with those observed in T47D cells. In T47D, H1.0/H1.2/H1.3/H1.5 are also more abundant in satellite and LINE classes than in SINE or SVA. However, genome-wide maps of H1X have not been generated in mouse nor in other cell models apart from those reported here.

To extend our results in T47D cells, we have performed ChIP-Seq of H1X in five additional cancer cell lines (i.e. SK-MEL-147, MCF-7, SK-N-SH, HeLa and HCT-116). H1X is universally enriched within SVAs and SINEs (Supplementary Figure S17). Importantly, in all cell lines analyzed, H1X abundance gradually increases along the evolution of SVAs and Alu lineage, as occurred in T47D cells. Although H1 variants distribution is thought to be cell-line specific (25), there is a lack of comparative studies addressing the question. In fact, our results suggest a universal association of H1X to SVA and Alu retrotransposons in human cancer cells. This evidence highlights the necessity of systematically evaluate endogenous H1 variants distribution among different cell models. Besides, the fact that the highest human H1X enrichment within TEs is found at SVAs and more concretely within human-restricted SVA families, begs the question of whether other lineage or species-specific TEs present a high H1X abundance in non-primate species which lack SVA retrotransposons. Actually, the balance between conserved TE regulatory mechanisms across species and species-specific TE regulation is poorly understood. On the one hand, a comparative study of human and chimpanzees iPSCs revealed that H3K9me3 profiles in orthologous TEs (including SVA, LTR and LINE-L1 elements), were overall conserved between both species (72). This study suggests that limited inter-species differences in TE silencing mechanisms exists in primates. On the other hand, in human differentiated cells, TEs are reported to act as enhancer elements in a cell-type specific manner (21,22,73,74). Thus, the comparative study of H1 variants genomic distribution within TE classes in different species emerges as another epigenetic layer to consider in the question. Similarly, the differential interplay of H1 variants abundance with other described repressive mechanisms such as histone modifications or DNA methylation is also an interesting matter of study.

Overall, we report that somatic H1 variants present heterogeneous binding profiles within the genome and more specifically within TE classes. Our results support histone H1 functional diversity in a variant-specific manner. H1 variants may be considered an additional repressive epigenetic mechanism to avoid deleterious retrotransposition, contributing to shape the genome during evolution.

## Supplementary Material

gkae014_Supplemental_File

## Data Availability

ChIP-Seq replicates of H1.3 in T47D cells have been deposited in NCBI’s Gene Expression Omnibus (GEO) and is accessible through GEO Series accession number GSE236878. ChIP-Seq data of H1X in five different cancer cell lines is available through the accession number GSE236678. Total RNA-Seq data from T47D H1Xsh cells treated or not with Dox and Che-RNA-Seq data performed in T47D cells are accessible through the accession number GSE236538 and GSE236881, respectively.
