# Supplementary material for: Genomic profiling of six human somatic histone H1 variants denotes that H1X accumulates at recently incorporated transposable elements
Source: Nucleic Acids Res. 2024 Jan 23;52(4):1793–813. doi: 10.1093/nar/gkae014 (PMC10899769; doi:10.1093/nar/gkae014)

Genomic profiling of six human somatic histone H1 variants denotes that H1X accumulates at recently incorporated transposable elements

Mónica Salinas-Pena[1,#], Núria Serna-Pujol[1,#], Albert Jordan[1,*]

SUPPLEMENTARY MATERIAL

SUPPLEMENTARY METHODS

Genome segmentations

To evaluate H1 variants abundance, different chromatin segmentations were used. Eight groups of Giemsa bands (G-bands) were defined according to (1) . Briefly, G-bands were classified as G positive (Gpos25 to Gpos100, according to its intensity upon Giemsa staining), and G-negative (unstained), which were further divided into four groups according to their GC content (Gneg1 to Gneg4, from high to low GC content). HeLa-S3 genome segmentation by ChromHMM (ENCODE) was obtained from UCSC human genome database (2). Genomic A and B compartments were obtained from T47D Hi-C experiments (3).

Chromatin-enriched (Che)-RNA extraction

Briefly, 40x10^6 T47D cells were lysed in 800 µL ice-cold Lysis Buffer A (10 mM Tris pH 7.5, 0.1% NP-40, 150 mM NaCl) for 5 min on ice. A sucrose cushion (24% sucrose in lysis buffer A) was used to collect nuclei upon centrifugation (200g, 4ºC,10min). Pellet was washed with pre-chilled PBS + 1mM EDTA, and resuspended in 500µL ice-cold Glycerol Buffer (20 mM Tris pH 7.9, 75 mM NaCl, 0.5 mM EDTA, 0.85 mM DTT, 0.125 mM PMSF, 50% glycerol). Nuclei were lysed by adding ice-cold Lysis Buffer B (10 mM HEPES pH 7.6, 1mM DTT, 7.5 mM MgCl2, 0.2 mM EDTA, 0.3M NaCl, 1M urea, 1% NP-40) and kept on ice for 10 min, with periodic vortex shaking. Pellet containing chromatin was obtained by centrifugation (14000g, 4ºC,2 min), washed twice with pre-chilled PBS+1mM EDTA, and finally resuspended in 100 µL PBS. Che-RNA were purified using TRIZOL reagent (Invitrogen), following the manufacturer's instructions.

Histones extraction

For isolation of total histones, cell pellets were resuspended in 1 ml of hypotonic solution [10 mM Tris–HCl (pH 8.0), 1 mM KCl, 1.5 mM MgCl2, 1 mM PMSF, 1 mM DTT] and incubated on ice for 30 min. The nuclei were pelleted at 10000 × g for 10 min at 4ºC. Sulfuric acid (0.2 M) was added to the pellet to extract the histones on ice for 30 min. The solution was centrifuged at 16000 × g for 10 min at 4ºC. TCA was added to the supernatant in order to precipitate histones. After >1h ice-incubation precipitate was centrifuged (16000xg 10 min at 4ºC). Precipitate was washed with acetone and finally resuspended in water. Protein concentration was determined by Micro BCA protein assay (Thermo Scientific) and immunoblot was performed.

Immunoblot

Histones samples were exposed to SDS-PAGE (14%), transferred to a PVDF membrane, blocked with 5% non-fat milk for 1 h, and incubated with primary antibodies overnight at 4◦C as well as with secondary antibodies conjugated to fluorescence (IRDye 680 goat anti-rabbit IgG or IRDye 800 goat anti-mouse IgG, Li-Cor) for 1 h at room temperature.

Bands were visualized in an Odyssey Infrared Imaging System (Li-Cor). Coomassie staining was used as loading controls.

H1.3 Immunoprecipitation (IP) from total histones

Total histones from T47D H1.3sh Untreated or 6-dayDox-treated cells were extracted as previously described. For IP reaction, 60 mg of histones per condition were incubated overnight at 4ºC in RIPA buffer (150mM NaCl, 50mM Tris pH 7.5, 1%NP-40, 0.5% Sodium Deoxycholate, 0.1%SDS, 1mM EDTA) with protease inhibitors, 5mL of anti-H1.3 ChIP-grade antibody (ref. ab203948) and 20mL of Protein A magnetic beads. Unbound histones were washed with RIPA buffer. After 3 washing steps, immunocomplexes were directly eluted from magnetic beads using Loading Buffer (5min 95ºC). Immunoblot analysis (see methods) were performed using 5 mg of input samples (histones) and 1/6 of total eluted IPed material. After anti-H1.3 IP, only histone H1.3 was detected. Importantly, after H1.3 IP in H1.3 KD conditions (i.e. +Dox), no pull-down of other H1 variants was observed by immunoblot, meaning that absence of H1.3 does not result in new putative antibody cross-reactions.

Immunofluorescence

Cells were fixed with 4% paraformaldehyde (20 min; room temperature), permeabilized with Methanol (10 min room temperature) and blocked with 5% bovine serum albumin. Primary antibody of interest was incubated overnight at 4ºC. Secondary antibodies conjugated to Alexa fluorophores (Invitrogen) were incubated 1h at room temperature. Nuclei were stained with Hoechst and coverslips mounted using Pro-long glass (Invitrogen). Samples were visualized in *Dragonfly 505* multimodal confocal microscope (Andor Technologies, Inc). Images were taken using 100x Objective and they were analysed using ImageJ software.

H1 variants peak calling

Peak calling of H1 variants ChIP-Seq in T47D cells was performed with MACS2 (v2.1.2) or DROMPA (v3.7.2) algorithms. For MACS2 peak calling, narrow peaks were computed *(callpeak –no model –ext size 200)*. DROMPA peak calling was performed with default parameters using *–pthre1 0.03 –qthre 0.03* as thresholding quality values. For H1 peak analyses within repeats, peaks overlapping the ENCODE BlackList regions were excluded from the analyses. Minimum 50% of the length of the peak was required to overlap a repeat to be considered as 'repetitive'.

Transcription factor binding profile of H1 peaks

Transcription factor (TF) binding data in T47D cells was obtained from ChIPAtlas database. H1 narrow peaks overlapping the different repetitive element classes/families were intersected with TF binding data to calculate the TF binding repertoire of each peak, requiring that at least 50% of the TF binding region overlaps the H1 peak. For comparison, TF binding in different subset of repeats was also calculated following the same procedure.

MNase-Seq analysis

MNase-Seq data was obtained from the GEO Series GSE74308 (Sample: GSM1916676). The processed supplementary data, available as a bigWig file, was used for computing the MNase-Seq signal profile around both real and random H1 enrichment peaks via deepTools (v3.5.1) (*computeMatrix* and *plotProfile*). Additionally, the bigWig file was

converted to BDG format with the *bigWigToBedGraph* Kent utility for computing the MNase-Seq average signal within H1 enrichment peaks using BEDTools (v2.28.0). The permutation test was carried out using in-house R scripts.

SUPPLEMENTARY TABLES

Supplementary Table S1. Oligonucleotides for semiquantitative PCR.

Supplementary Table S2. Read count report of H1 variants ChIP-seq data included in the manuscript.

Supplementary Table S3. Read count report of H1 variants ChIP-seq data mapping within each TE class, shown as percentage.

Supplementary Table S4. Repetitive elements classified according to taxonomic clades. Table includes clade classification of repeats within each family. Number and percentage of repeats belonging to each clade (N=7) is indicated. Clade classification information is extracted from Dfam database (4). Unknown class (N=29 families) is not included in the table but all Unknown repeats are classified as "Non-Primate". Notably, TE-transcripts repeat annotation includes N=41 Alu families, but AluYf4, AluYc5 and AluYf5 families are not listed in Dfam database, so N=38 Alu families are included from evolutionary analysis.

Supplementary Table S5. Hominoidea repeats. Table shows the repeat name of N=48 repeats found in *Hominoidea* and descendant clades (from *Hominoidea* to *H.sapiens*). Repetitive element class and family are indicated.

SUPPLEMENTARY FIGURE LEGENDS

Supplementary Figure S1. Analysis of H1.3 antibody specificity and ChIP performance. A) Immunoblot analysis of H1 variants in histone extracts from inducible single-H1 KDs or Randomsh in T47D (untreated or Dox-treated). Coomassie staining is added as immunoblot loading control. **B)** Immunoblot analysis of inducible single H1.3 KD T47D cells chromatin extracts. **C)** ChIP-qPCR of H1.3 in inducible H1.3 KD T47D cells. H1.2 was added as a positive control and non-specific IgG as a negative control. Upon ChIP, resulting immunoprecipitated DNA was amplified by qPCR with oligos for distal promoter (3kb upstream TSS) and TSS regions of genes CDK2 and NANOG. ChIP amplification is shown relative to input DNA amplification. In CDK2 (active gene) H1 depletion in TSS compared to Upstream regions is observed but not in case of NANOG (inactive gene) as previously defined for other variants (5). Statistical differences between untreated and +Dox immunoprecipitated DNA by H1.3 antibody are supported by *t*-test. (**) p-value<0.05. **D)** Recombinant human histone H1 variants (125 ng) were immunoblotted with the indicated H1.3 antibody. All recombinant H1 variants are native except for 6xHis-HA-H1.2 and 6xHis-H1.4. Coomassie staining is shown as a loading control. **E)** Immunoblot analysis of the indicated H1 variants in total histone extracts of T47D and cell lines naturally lacking H1.3 expression (i.e. HeLa and MDA-MB-231). **F)** Immunofluorescence analysis of H1.3 and H1.0 in T47D, HeLa and MDA-MB-231. Immunofluorescence quantification of H1.3 signal in the three different cell lines is shown. **G)** Immunoblot analysis of H1 variants expression within cell lines. Chromatin extracts (10 µg of protein) from T47D, HeLa and HCT-116 cells were run in SDS/PAGE and immunoblotted with the indicated antibodies against histone H1 variants or histone H3 as a loading control. HeLa and HCT-116 cell lines naturally lack H1.3 expression. **H)** ChIP-qPCR of H1 variants in T47D, HeLa and HCT-116 cells. Chromatin was used for ChIP with antibodies against H1 variants and unrelated IgG as a control. Resulting DNA was amplified by qPCR with oligos for distal promoter (3kb upstream TSS) and TSS regions of genes CDK2 and NANOG. ChIP amplification is shown relative to input DNA amplification. A representative experiment is shown. Statistical differences between H1-IPed DNA and IgG are supported by *t*-test. (**) p-value<0.01; (***) p-value<0.001. **I)** Only H1.3 was detected after H1.3 immunoprecipitation from histones extract. Total histones from T47D H1.3shRNA cells untreated or 6-days Dox-treated were immunoprecipitated with the H1.3 antibody (ab203948). Immunoblot analysis with the indicated histone H1 antibodies was performed on input and immunoprecipitated material. Coomassie staining of samples is shown as a loading control.

Supplementary Figure S2. Genome profiling of six somatic histone H1 variants in breast cancer cells. A) Heatmap and cluster analysis of the input-subtracted ChIP-Seq abundance (scaled) of H1 variants (T47D cells) within Giemsa bands (G-bands). Y-axis annotation shows to which group the G-band belongs according to the legend. The relative GC content of the different G-bands is indicated. Two ChIP-Seq replicates are shown (r1, r2). Briefly, G-bands can be used as epigenetic units to compute H1 variants abundance, as previously reported in (1, 3). For details regarding G-bands classification and characterization, see (1). **B)** Heatmap and cluster analysis of the input-subtracted ChIP-Seq abundance (scaled) of H1 variants within A/B compartments. Y-axis annotation shows compartment identity according to the legend. **C)** Heatmap and cluster analysis of the median input-subtracted ChIP-Seq abundance (scaled) of H1 variants within 10 Chromatin states. Each row corresponds to a different chromatin state, according to the

Y-axis annotation and the legend. **D)** H1 variants Input-subtracted ChIP-Seq average profile around gene transcriptional start site (TSS). Expressed genes are divided in 10 equal groups (Groups 1-10, each containing 10% of total expressed genes) according to their basal gene expression on T47D RNA-Seq experiments. Group 0 includes non-expressed genes. Average H1 profile for all genes is shown in black. H1.3 is present at TSS according to the transcriptional status of the gene, as previously defined for the other H1 variants (3, 5). **E)** IGV genome browser capture of H1 variants and H1 narrow peaks. Two replicates (r1,r2) of H1 variants ChIP-Seq are shown (Input-subtracted ChIP-Seq signal). In the upper panel (50Mb region), genome A and B compartments in T47D cells are included. In the bottom panel, a 50 kb region (A compartment) is zoomed-in. For H1.4 and H1X, H1 Input-subtracted abundance is shown parallel to H1 narrow peaks calculated with MACS2. For the other variants, H1 narrow peaks cannot be computed. Alu elements (green) and SVA repeats (brown) are included to show that H1.4/H1X narrow peaks highly coincide with these repeats. **F)** Boxplot of H1X input-subtracted ChIP-Seq abundance within repetitive (*R*) or non-repetitive (*NR*) genome fractions intersecting A or B compartment segments or gene coding regions (*C*). Gene coding regions were defined as any region overlapping a protein-coding transcript from Emsenbl annotation. Statistical differences between H1X abundance at the indicated genome fractions are supported by One-sample Wilcoxon signed-rank test (***) p-value<0.001.

Supplementary Figure S3. Classification and profiling of repetitive elements. A) Repeat elements are classified in classes (N=9), which are further categorized into families (N=70), formed by different repeats (N=966) which are present in multiple copies along the genome. The number of repeat types present within each family is indicated, as well as the total number of copies of repeats and its occupancy within the genome (expressed in base-pairs and percentage) for each family of repeats. **B)** Genome occupancy (total length in bp) of the repeat families within each class is represented in a pie chart and expressed as percentage. Families are ordered based on their decreasing genome occupancy within their respective class. **C)** Occupancy of repetitive and non-repetitive genome fractions within the A and B compartments. Overlap (in bp) between repetitive elements annotation and each compartment was calculated and expressed as percentage. **D)** Distribution of each repetitive element class within A or B compartments. Total length (bp) occupied for each class and their overlap with A or B compartment was calculated and expressed as percentage. **E)** Genome occupancy (total bp contribution to the genome) of repetitive elements classes, expressed as percentage.

Supplementary Figure S4. Detection of H1X and H1.4 narrow peaks within repetitive element classes. A) Table contains the number of narrow peaks for H1 variants ChIP-Seq computed with MACS2. In T47D, narrow peaks can only be efficiently called for H1X and H1.4. **B)** Boxplots show length distribution of H1X and H1.4 MACS2 narrow peaks, comparing all peaks and peaks overlapping repeats. **C)** Table including the number of H1 MACS2 narrow peaks per family, considering total number of peaks within the family or restricting one peak per repeat copy. Data is also expressed as percentage of peaks per family or percentage of repeats exhibiting at least one peak. In longer repeats, one single copy could harbor more than one H1 peak, so in order to properly calculate the percentage of repeats exhibiting narrow peaks, a maximum of one peak per repeat is considered (so, repeats could have one or more H1 narrow peaks). Number of repeat copies include those located in chr1-22 and chrX and excluded from ENCODE BlackList (6). **D)** Table

contains the number of peaks for H1 variants ChIP-Seq computed with DROMPA. **E)** Percentage of H1 DROMPA peaks that overlap or not with any type of annotated repeat. Minimum 50% of the length of the peak is required to overlap a repeat to be considered. **F**) Venn Diagram showing the overlap between H1X or H1.4 peak calling performed with MACS2 or DROMPA. **G)** Number of H1 DROMPA peaks in repeats per repetitive element class, calculated as number of peaks in each class divided by number of peaks in repeats, and expressed as percentage. Genome occupancy of repeats, considering as 100% the 48.78% of the genome which is repetitive is shown as a reference.

Supplementary Figure S5. Enrichment of H1X and H1.4 narrow peaks within repetitive elements A) Percentage of SVA repeats in each family that contain at least one H1 narrow peak. **B)** Percentage of Alu repeats that contain at least one H1 narrow peak. Alu repeats are ordered by its decreasing percentage of repeats marked by H1.4. **C)** Alu repeats classified into sub-families that contain at least one H1 narrow peak, represented as number of H1 narrow peaks relative to total number of repeat copies per sub-family. Alu sub-families are ordered by their evolutionary age (7). **D)** H1 narrow peaks within the top-20 most evolutionary recent repeats of the LINE-L1 family (7). Data is expressed as total H1 narrow peaks corrected by total base-pairs occupied per each LINE repeat. **E)** Percentage of LTR repeats of the ERV1 and ERVL-MaLR families that contain at least one H1 narrow peak. LTR repeats more extensively marked by H1 are shown. Repeats are ordered by decreasing percentage of elements containing H1X peaks. **(A-E)** Number of repeat elements from each class which are more marked by H1X/H1.4 according to MACS2 analyses are: SVAs: SVA_A (247), SVA_B (461), SVA_C (277), SVA_D (1341), SVA_E (233), SVA_F (967); Alu elements: AluYa5 (3694), AluYf5 (177), AluYb9 (312), AluYb8 (2712); LINE-L1: L1HS (1388), L1PA2 (4402), L1PA3 (9747), L1PA4 (11090), L1PA5 (10708); LTR-ERV1: LTR12C (2576), LTR12E (99), LTR12D (434). **F)** Genomic annotation of H1X or H1.4 MACS2 narrow peaks within the indicated repetitive element classes/families. Genomic annotation of the repeat classes/families evaluated is shown as a reference. Percentage values of annotated peaks are included in the bottom table, where 'All' column refers to the annotation of the indicated repeat class/family. **G)** Transcription factor binding profile of H1X or H1.4 MACS2 narrow peaks within repetitive element classes/families. Data is expressed in percentage (%) of H1 peaks or repeats marked by the different transcription factors as indicated in the legends. For each repeat class/family the transcription binding profile of the whole class/family is shown as a reference. Besides, concrete subset of repeats in which H1X/H1.4 peaks tend to be concentrated are also shown as a reference. Alu groups are defined according to Figure 3B. Transcription binding profile in T47D cells was extracted from ChIPAtlas database. The 'Other' category includes multiple transcription factors with minimal binding to H1 peaks.

Supplementary Figure S6. Abundance of H1 variants within individual repeats. Meta-repeat profile of H1 variants input-subtracted ChIP-Seq abundance at repeats and their 3kb flanking regions. In the heatmaps, each row represents a repeat of the indicated class, ordered by the corresponding H1 profile in each case (i.e., heatmaps show an independent order for each H1 variant).

Supplementary Figure S7. H1X abundance and genomic annotation of SVA TEs. A) Boxplot of H1 variants input-subtracted ChIP-Seq abundance within the SVA subfamilies (N=11) stablished by Levy et al. (8) and ordered by their evolutionary age. In boxplots,

gray horizontal line indicates H1 variant median abundance within all repeat from different classes while red horizontal line corresponds to H1 variant abundance within SVA repeats. Kruskal-Wallis test determined that differences in H1 variant abundance between SVA subfamilies exist (p-value<0.001) except for H1.3 and H1.4 (non-significant). One-sample Wilcoxon signed-rank test was used to statistically support H1 enrichment (blue asterisks) or depletion (red asterisks) compared to the median H1 abundance within all SVA repeats. (***) p-value < 0.001; (**) p-value < 0.01; (*) p-value < 0.05. **B)** Genomic annotation of the different families of SVA repeats.

Supplementary Figure S8. H1 variants and chromatin features within SVA elements. A) Heatmap and cluster analysis of the median input-subtracted ChIP-Seq abundance (scaled) of H1 variants (T47D cells) and other chromatin features within 10 Chromatin states. Each row corresponds to a different chromatin state, according to the Y-axis annotation and the legend. Average RNA-seq, cheRNA-seq, ATAC-seq and MeDIP-seq signal for each Chromatin state are included, annotated from high to low relative levels. **B)** Meta-repeat profile of H1 variants (T47D cells) and other chromatin features input-subtracted ChIP-Seq abundance at SVA repeats and their 3kb flanking regions. In the heatmaps, each row represents a SVA repeat of the indicated family and rows are ordered by the corresponding feature profile in each case. Average profile per family is also shown in the upper line graphs. **C)** Up pie charts: Count and percentage of SVA elements that overlap with H1X (left) and ZNF91 (right) enrichment regions. Down pie charts: Count and percentage of H1X (left, orange) and ZNF91 (right, blue) enrichment regions that overlap with SVA repeats. **D)** H1 variants (T47D cells) input-subtracted ChIP-Seq profiling at regions of ZNF91 or KAP1 enrichment (MACS2 peaks) and their 3kb flanking regions.

Supplementary Figure S9. H1X and ZNF91 abundance at SVA_F elements and their flanking regions. A) Meta-repeat profile of H1X, ZNF91, KAP1, DNA methylation and RNApolII input-subtracted ChIP-Seq abundance at SVA_F repeats and their 1kb flanking regions. SVA_F elements are divided in four groups considering whether they are isolated (alone) within the genome, or flanked by other SVA elements in 5’ (last), 3’ (first), or 5’ and 3’ simultaneously (internal). **B)** IGV genome browser of SVA elements (in tandem or alone) showing the input-subtracted ChIP-Seq abundance of H1X and ZNF91. **C)** Meta-repeat profile of H1X and ZNF91 input-subtracted ChIP-Seq signal using only uniquely mapped reads at SVA_F repeats and their 1kb flanking regions. SVA_F elements were classified into four groups depending on whether they are isolated (Alone) or preceded (Last), followed (First), or flanked on both sides (Intern) by other SVA elements. **D)** Meta-repeat profile of H1X input-subtracted ChIP-Seq signal including multi-mapping reads (Multi) or using only uniquely mapped reads (Unique) at SVA, SINE (N=5 SINE groups and N=12 selected Alu; as considered in Figure 3), and LINE (N=6 L1 groups and N=18 L1PA-L1HS groups; as considered in Figure 4) repeats and their 3kb flanking regions. **E)** IGV genome browser capture showing the input-subtracted ChIP-Seq signal of histone H1X in SVA repeats (pink squares) including multi-mapping reads or using only uniquely mapped reads.

Supplementary Figure S10. Relationship between H1 variants and different chromatin factors within SVA repeats. A) Spearman’s correlations between H1X input-subtracted ChIP-Seq signal and H1.4, ZNF91, MeDIP-seq and ATAC-Seq signals within SVA families.

B) Differential (log2) Spearman's correlation between all the features analyzed in SVA_F compared to SVA_A, using uniquely mapped reads.

Supplementary Figure S11. H1 variants and chromatin features within SINE elements and MNase profile at H1X/H1.4 peaks. A) Meta-repeat profile of H1 variants (T47D cells) and other chromatin features input-subtracted ChIP-Seq abundance at SINE repeats divided in 5 groups according to **Figure 3B** and their 3kb flanking regions. **B)** Profile of MNase-Seq signal around H1X (top) and H1.4 (bottom) observed enrichment peaks compared to a set of H1X and H1.4 random peaks, respectively. **C)** Histogram of the median differences from the permutation test between MNase-Seq quantification in H1X (top) and H1.4 (bottom) actual and random peaks. A total of N=10,000 permutations were performed. The colored vertical bars correspond to the observed difference in medians between the MNase-Seq signal in H1X and H1.4 real peaks compared to random peaks.

Supplementary Figure S12. Relationship between H1 variants and different chromatin factors within SINE repeats grouped by their evolutionary age. A) Boxplot of basal RNA-Seq expression, MeDIP-Seq quantification, and input-subtracted ChIP-Seq abundance of different features within 12 SINE groups, separated between non-expressed (*Ne*) and expressed (≥3 uniquely-mapped RNA-Seq reads; *E*) SINEs. Statistically significant difference between not expressed (Ne) and expressed (E) SINE repeats was assessed using the Mann–Whitney U test (***) $P < 0.001$; (**) $P < 0.01$; (*) $P < 0.05$. **B)** Spearman's correlation between all the features analyzed in SINEs groups 1 to 5, using only uniquely mapped reads. Only correlations with p-value < 0.01 were considered (colored squares in the correlation matrices). **C)** Differential (log2) Spearman's correlation between all the features analyzed in SINE group 5 compared to group 1, using uniquely mapped reads.

Supplementary Figure S13. H1 variants within LINE families. A) Heatmap and cluster analysis of the average input-subtracted ChIP-Seq abundance (scaled) of H1 variants (T47D cells) within the groups of LINE repeats (N=146) belonging to the 6 families.

Supplementary Figure S14. H1 variants and chromatin features within LINE elements. A) Meta-repeat profile of H1 variants (T47D cells) and other chromatin features input-subtracted ChIP-Seq abundance at L1 LINE repeats divided in 6 subfamilies and their 3kb flanking regions. In the heatmaps, each row represents a LINE repeat of the indicated group and ordered by the corresponding feature profile in each case. Average profile per group is also shown in the upper line graphs. **B)** Meta-repeat profile of H1 variants (T47D) input-subtracted ChIP-Seq signal at L1 LINE repeats from six subfamilies and their 3kb flanking regions. Each subfamily was divided into two groups showing the 10% highest (top panel) or lowest (bottom panel) H3K9me3 signal. **C)** Detailed examination of the meta-repeat profiles of MeDIP-Seq signal and input-subtracted ChIP-Seq abundance of H1 variants and different chromatin features at six selected L1PA-L1HS groups and their 3kb flanking regions.

Supplementary Figure S15. Relationship between H1 variants and other chromatin factors within LINE repeats grouped by their evolutionary age. A) Boxplot of basal RNA-Seq expression, MeDIP-Seq quantification, and input-subtracted ChIP-Seq abundance of different features within 18 L1PA-L1HS groups, separated between non-expressed (*Ne*) and expressed (≥3 uniquely-mapped RNA-Seq reads; *E*) LINEs. Statistically significant difference between not expressed (Ne) and expressed (E) LINE repeats was assessed

using the Mann–Whitney U test (***) P < 0.001; (**) P < 0.01; (*) P < 0.05. **B)** Spearman's correlation between all the features analyzed in LINEs subfamilies, using only uniquely mapped reads. Only correlations with p-value < 0.01 were considered (colored squares in the correlation matrices). **C)** Differential (log2) Spearman's correlation between all the features analyzed in LINE subfamily L1PA_L1Hs compared to L1ME, using uniquely mapped reads. **D)** Spearman's correlation between all the features analyzed in L1PA_L1HS subgroup elements (L1PA17 to L1HS), using only uniquely mapped reads.

Supplementary Figure S16. H1X and H1.4 are enriched within transposable elements recently incorporated in the genome along primates evolution. Heatmap and clustering of H1 variants input-subtracted ChIP-Seq median abundance (scaled) at N=48 groups of repeats from *Hominoidea* and descendant clades (i.e. repeats listed in Supplementary Table S4). Y-axis annotation indicates repetitive element family and taxonomic clades.

Supplementary Figure S17. H1X from different cell lines is enriched within transposable elements recently incorporated in the genome along primates evolution. A) Boxplot of H1X input-subtracted ChIP-Seq signal within repetitive element classes in five cancer cell lines. **B)** Meta-repeat profile of H1X Input-subtracted ChIP-Seq abundance in five cancer cell lines at SVA (N=6 families) and SINE (N=5 SINE groups and N=12 selected Alu; as considered in Figure 3) repeats and their 3kb flanking regions. In the heatmaps, each row represents a SVA repeat of the indicated family and rows are ordered by the corresponding H1 profile in each case. Average profile of H1X per family is also shown in the upper line graphs. **C)** Boxplot analysis of H1X input-subtracted ChIP-Seq abundance in five cancer cell lines within repetitive elements classified into different taxonomic clades according to Dfam database, ordered by their evolutionary age. The classification of repeats corresponds to the taxonomic clade to which the oldest ancestor in which the repeat has been found, belongs. **D)** Boxplots indicate the H1X input-subtracted ChIP-Seq abundance in five cancer cell lines within repeats of Other Class (i.e. SVA families) and SINE-Alu family, classified according taxonomic clades. **A,C,D)** In boxplots, gray horizontal line indicates median H1X abundance within all repeats. Red horizontal line in (D) indicates median H1X abundance within all SVA repeats (upper graphs) or within all Alu family (bottom graphs). One-sample Wilcoxon signed-rank test was used to statistically support H1X enrichment (blue asterisks) or depletion (red asterisks) compared to the median H1X abundance within all repeats (A,C), to the median H1X abundance within all SVAs (D, upper graphs) or to the median H1X abundance within all Alu family (D, bottom graphs). (***) p-value <0.001; (**) p-value<0.01; (*) p-value < 0.05. **E)** Meta-repeat profile of H1X Input-subtracted ChIP-Seq abundance in six cancer cell lines at LTR12C/E repeats and their 3kb flanking regions. In the heatmaps, each row represents a LTR repeat of the indicated group and rows are ordered by the corresponding H1 profile. Average profile of H1X is shown in the upper line graphs.

Supplementary Figure S18. ChIP-qPCR confirms enrichment of H1X within recently incorporated TEs in cancer cells. Resulting DNA after ChIP of H1 variants in T47D (A-C) or other cell lines (D) was amplified by qPCR with oligos for specific repetitive elements. ChIP amplification is shown relative to input DNA amplification in graphs showing H1X abundance. Additionally, relative H1 variants content (ratio) within repeats was calculated. **A**) H1X abundance or H1X/H1.2 ratio within repeats. H1X is present within

repeats including satellites compared to TSS regions where H1 variants are in generally depleted. However, the highest H1X enrichment is found within SVAs. Besides, relative H1X/H1.2 ratio is variable among satellite repeats. Statistical significance of H1X enrichment at repeats compared to TUBGCP5 gene TSS is indicated. **B**) H1X abundance or normalized H1X/H1.2 ratio within young repeats from SVA or Alu families (SVA_F, AluYa5), SATa or evolutionary-old LTRs. SVA_F and AluYa5 present a higher H1X abundance and H1X/H1.2 ratio. H1X/H1.2 ratio values are normalized (range values 0-1). r1-r3 refers to different biological replicates. Statistical differences in H1 abundance or ratio at SVA_F and AluYa5 compared to the rest of the repeats evaluated are indicated. **C**) Heatmap represents normalized H1X/"low-GC"-H1 ratios within repeats. SVA_F and AluYa5 present a higher relative content of H1X and are more depleted from low-GC variants (i.e. higher ratio) compared to SATa or old LTRs, which are relatively more enriched in low-GC variants compared to H1X (i.e. lower ratio). Statistics refer to the difference between the indicated H1 ratio at SVA_F and AluYa5 compared to the ratio of the remaining repeats. **D**) H1X abundance within repeats in different cell lines. The highest H1X abundance within repeats in SK-MEL-147, SK-N-SH and HCT-116 cell lines is found at SVA repeats. Notably, relative abundance at satellite repeats (SATa) is variable among cell types, as also denoted in Figure S17A. Statistical significance of H1X enrichment at SVA_F compared to the rest of the repeats in the three cell lines is indicated. Classification and evolutionary age of some repeats evaluated: HERVK ("Hominoidea" LTR); AluYa5 ("Hominidae" SINE-Alu; SINE Group 5); MLT1C49, MLT1J2 ("Non-primate" LTRs); SATa, D4Z4 and ACRO1 (Satellites). For all panels, statistical differences are supported by *t*-test. (*) p-value<0.1; (**) p-value<0.05.

Supplementary Figure S19. Transposable elements expression changes upon H1X depletion. A) Immunoblot analysis of H1 variants in histone extracts from inducible H1X shRNA T47D-derived cells. Coomassie staining is added as immunoblot loading control. **B)** Volcano plots of TEs expression changes upon H1X, H1.4 or H1.2 KD or Random shRNA control using uniquely mapped RNA-Seq reads. Up and down-regulated LINE, SINE or SVA elements are colored in blue/green/yellow or red, respectively. Repeats with a |FC|>1.5 and a p-adjust<0.05 were considered as significantly deregulated. **C)** Percentage of SVA elements that showed an increase or decrease of RNA-seq reads upon H1X KD dividing by the indicated groups of elements. **D)** Pie-chart showing the proportion of up-regulated SVAs belonging to each family upon H1X KD. Up-regulated SVAs are defined as SVAs that presented RNA-Seq reads in basal conditions (-Dox) and reads were increased in H1X KD (+Dox) (X to +X). For comparison, the proportion of total SVAs belonging to each family is shown. **E)** H1X depletion and Aza treatment have an additive effect in activating young transposable elements. Combined Dox (H1X KD induction) and Aza (DNMT inhibitor) treatment was performed and expression was analyzed by RT-qPCR. Figure shows RT-qPCR expression of different genes or repetitive elements. KRT37 gene is among the top upregulated genes upon H1X depletion. MAL is an Aza-responding gene. H1X expression was measured to show H1X KD efficacy. Expression was corrected by GAPDH and expressed relative to Untreated condition. Statistical differences between +Dox and +Dox+aza are supported by *t*-test. (*) p-value<0.1; (**) p-value<0.05.

REFERENCES

1.Serna-Pujol,N., Salinas-Pena,M., Mugianesi,F., Lopez-Anguita,N., Torrent-Llagostera,F., Izquierdo-Bouldstridge,A., Marti-Renom,MA., Jordan,A. (2021) TADs enriched in histone H1.2 strongly overlap with the B compartment, inaccessible chromatin and AT-rich Giemsa bands. FEBS J, 288, 1989–2013.

2. Navarro Gonzalez,J., Zweig,A.S., Speir,M.L., Schmelter,D., Rosenbloom,K.R., Raney,B.J., Powell,C.C., Nassar,L.R., Maulding,N.D., Lee,C.M., *et al.* (2021) The UCSC Genome Browser database: 2021 update. *Nucleic Acids Res*, **49**, D1046–D1057.

3. Serna-Pujol,N., Salinas-Pena,M., Mugianesi,F., Le Dily,F., Marti-Renom,M.A. and Jordan,A. (2022) Coordinated changes in gene expression, H1 variant distribution and genome 3D conformation in response to H1 depletion. *Nucleic Acids Res*, **50**, 3892–3910.

4. Storer,J., Hubley,R., Rosen,J., Wheeler,T.J. and Smit,A.F. (2021) The Dfam community resource of transposable element families, sequence models, and genome annotations. *Mob DNA*, **12**, 2.

5. Millán-Ariño,L., Islam,A.B.M.M.K., Izquierdo-Bouldstridge,A., Mayor,R., Terme,J.M., Luque,N., Sancho,M., López-Bigas,N. and Jordan,A. (2014) Mapping of six somatic linker histone H1 variants in human breast cancer cells uncovers specific features of H1.2. *Nucleic Acids Res*, **42**, 4474–4493.

6. Amemiya,H.M., Kundaje,A. and Boyle,A.P. (2019) The ENCODE Blacklist: Identification of Problematic Regions of the Genome. *Sci Rep*, **9**, 9354.

7. Giordano,J., Ge,Y., Gelfand,Y., Abrusán,G., Benson,G. and Warburton,P.E. (2007) Evolutionary history of mammalian transposons determined by genome-wide defragmentation. *PLoS Comput Biol*, **3**, e137.

8. Levy,O., Knisbacher,B.A., Levanon,E.Y. and Havlin,S. (2017) Integrating networks and comparative genomics reveals retroelement proliferation dynamics in hominid genomes. *Sci Adv*, **3**, e1701256.

Suppl. Table S1.

	SENSE	SEQUENCE (from 5' to 3')
H1.4	F	GTCGGGTTCCTTCAAACTCA
	R	CTTCTTCGCCTTCTTTGGG
H1X	F	CCCAACGATGTAGCGTTTTT
	R	AAGGCCGAGAGCCAATAGA
GAPDH	F	GAGTCAACGGATTTGGTCGT
	R	TTGATTTTGGAGGGATCTCG
ZNF91	F	CCAGACCTGATTACTTATCTGG
	R	ACATTTTTCATATTTCTCAGTAATAC
KRT37	F	TGGGGAGATGATTCTGAAGG
	R	TGCTACCGGTTGATTTAGGG
MAL	F	TTTTACCTCAGCGCCTCAGT
	R	ACACCATCTGGGTTTTCAGC
CDK2 Distal	F	CAGCGAGGAAAGTCACATCA
	R	TGGGGTGAGGGTAGTTTCTG
CDK2 TSS	F	GCGGCAACATTGTTTCAAGT
	R	GTCGGGATGGAACGCAGTAT
NANOG Distal	F	GACAGGGTTTCACCATGTTGGT
	R	CCGAGCCAGGTGCATCAT
NANOG TSS	F	CGGTTTTCTAGTTCCCCACCTA
	R	CCAAGGCCATTGTAATGCAA
HERVK	F	AGAGGAAGGAATGCCTCTTGCAG
	R	TTACAAAGCAGTATTGCTGCCCGC
HERVE-int	F	ACTGGCCTTTTCCTAGGTGATAC
	R	TACTATTAATGGCTGCACAAGCA
LTR12C	F	GTCTCGCTGGCTCAGGAGTG
	R	TGAGCTGTAACACTCACCGC
LTR5_Hs	F	AAAGGGTCTGTGCTGAGGAG
	R	AGACATTCCATTGCCCAGGG
L1HS_5end #1	F	TCCATCTGAGGTACCGGGTT
	R	CGATTTTCCAGGTGCGTCC
L1HS_5end #2	F	GGCACACTGACACCTCACA
	R	AGATGGGTTTTCGGTGTGGA
L1HS_3end	F	GGGCGAAGGACATGAACAGA
	R	CCTCTCCAGCACCTGTTGTT
L1PA3	F	GGGCGAAGGACATGAACAGA
	R	GCCAGTGATGGTGAGCATTTT
L1PA4	F	CATTTGCGGTTCACCAATATC
	R	GCTAGAGGTCCACTCCAGAC
AluYa5	F	AGGAGATCGAGACCATCCCG
	R	CCACTACGCCCGGCTAATTT
AluYb8	F	CGAGGCGGGTGGATCATGAGGT
	R	TCTGTCGCCCAGGCCGGACT
SVA_A #1	F	TCTCGCTCACTCAATGCTCA
	R	CTGGGAGGTGGAGGTTGTAG
SVA_A #2	F	CTCGCTCACTCAATGCTCAA
	R	GAGGTGGAGGTTGTAGCGA
SVA_E	F	AATAGAAAGGCGGGAAGGGTG
	R	CTTCTATCCACACAGACCCGG
SVA_F #1	F	GACTGGTTTTGGTGGAGACG
	R	GGAGGTGTAGGTTGTAGCGA
SVA_F #2	F	CCTGACTGGTTTTGGTGGAG
	R	GCTGGGAGGTGTAGGTTGTA
SVA_F #3	F	TGTGGAATAGAAAGGCGGGA
	R	AGGATCCCAAGGCAGAGGA
SVAs #1	F	GTGTACCCAACAGCTCATTG
	R	CACGGCAACCATCCGATTTC
SVAs #2	F	GCCTTGGGATCCTGTTGATC
	R	CTTAACGAGCATGCTGCCTTC
SST1/NBL2	F	AACCACTGTGACGGGAGAAA
	R	CTGGGACAGGACGAGACAC
SATa	F	AAGGTCAATGGCAGAAAAGAA
	R	CAACGAAGGCCACAAGATGTC
MLTC49	F	TATTGCCGTACTGTGGGCTG
	R	TGGAACAGAGCCCTTCCTTG
MLT1J2	F	CCTGGGTCCCTGAGTCACTA
	R	TGCCAGCTGCTGTAACAAAC

Suppl. Table S1. Oligonucleotides for semiquantitative PCR.

Suppl. Table S2.

Sample	Total reads	Overall alignment rate	Uniquely mapped reads		Multi-mapping reads	
			Number	Percentage	Number	Percentage
**Input**	78323763	97.86%	53341118	68.10%	23310328	29.76%
**H1.0**	46971404	97.64%	34112795	72.62%	11751484	25.02%
**H1.2**	39491876	98.53%	29006867	73.45%	9906339	25.08%
**H1.3**	22298829	97.49%	16084959	72.13%	5653718	25.35%
**H1.4**	65443912	94.84%	29743457	45.45%	32322092	49.39%
**H1.5**	41749958	98.46%	30478718	73.00%	10628648	25.46%
**H1X**	24202761	94.74%	9806287	40.52%	13123578	54.22%

Suppl. Table S2. Read count report of H1 variants ChIP-seq data included in the manuscript.

Suppl. Table S3

	Input (78323763)	H1.0 (46971404)	H1.2 (39491876)	H1.3 (22298829)	H1.4 (65443912)	H1.5 (41749958)	H1X (24202761)
**SVA**	0,16	0,11	0,1	0,13	0,27	0,11	0,56
**SINE**	15,51	12,26	12,2	13,06	29,53	12,41	32,02
**Satellite**	0,21	0,23	0,23	0,23	0,21	0,23	0,2
**LINE**	18,59	19,74	19,82	18,98	17,97	19,98	17,68
**LTR**	8,89	9,05	9,34	9,11	6,68	9,4	6,18
**DNA**	2,99	3,33	3,35	3,22	1,87	3,31	1,49
**RC**	0,01	0,02	0,02	0,01	0,01	0,02	0
**RNA**	0	0	0	0	0	0	0
**Unknown**	0,04	0,04	0,05	0,04	0,02	0,05	0,01
**Total**	**46,42**	**44,78**	**45,12**	**44,79**	**56,56**	**45,51**	**58,16**

Suppl. Table S3. Read count report of H1 variants ChIP-seq data mapping within each TE class, shown as percentage.

Suppl. Table S4

REPEAT CLASSIFICATION			CLADES (n= 7)													
Class (n=9)	Family (n=72)	Repeat (n=963)	Non-Primates		Primates		Simiiformes		Catarrhini		Hominoidea		Hominidae		H.sapiens	
			n	%	n	%	n	%	n	%	n	%	n	%	n	%
DNA	DNA	12	11	91.7			1	8.33								
	hAT	9	9	100												
	hAT-Blackjack	8	8	100												
	hAT-Charlie	68	62	91.2	1	1.47	5	7.35								
	hAT-Tip100	24	24	100												
	PiggyBac	6	2	33.3			4	66.67								
	TcMar	6	6	100												
	TcMar-Mariner	5	2	40	1	20	2	40								
	TcMar-Tc2	8	8	100												
	TcMar-Tigger	52	24	46.2	28	53.85										
	Merlin	1		0	1	100										
	MuDR	5		0	5	100										
	**12**	**204**														
LINE	CR1	15	15	100												
	Dong-R4	1	1	100												
	L1	122	82	67.2	31	25.41	1	0.82	2	1.64	4	3.28	2	1.64		
	L2	4	4	100												
	RTE	3	3	100												
	RTE-BovB	1	1	100												
	**6**	**146**														
LTR	ERV	3	3	100												
	ERV1	260	48	18.5	94	36.15	67	25.77	36	13.85	8	3.08	7	2.69		
	ERVK	37	0	0			2	5.41	24	64.86	10	27.03			1	2.70
	ERVL	102	85	83.3	10	9.80	7	6.86								
	ERVL-MaLR	80	58	72.5	9	11.25	12	15	1	1.25						
	Gypsy	19	19	100												
	LTR	3	3	100												
	**7**	**504**														
Satellite	Satellite	15	3	20	12	80										
	acro	1		0	1	100										
	centr	6		0	6	100										
	telo	2		0	2	100										
	**4**	**24**														
SINE	Alu	38	1	2.6	27	71.05							10	26.32		
	Deu	2	2	100												
	MIR	4	4	100												
	SINE	1	1	100												
	**4**	**45**														
Other	SVA_A	1		0							1	100				
	SVA_B	1		0									1	100		
	SVA_C	1		0									1	100		
	SVA_D	1		0									1	100		
	SVA_E	1		0											1	100
	SVA_F	1		0											1	100
	**6**	**6**														
**RC**	**Helitron**	**4**	**4**	100												
**RNA**	**RNA**	**1**	**1**	100												

Suppl. Table S4. Repetitive elements classified according to taxonomic clades. Table includes clade classification of repeats within each family. Number and percentage of repeats belonging to each clade (n=7) is indicated. Clade classification information is extracted from Dfam database (4). Unknown class (n=29 families) is not included in the table but all Unknown repeats are classified as “Non-Primate”. Notably, TE-transcripts repeat annotation includes n=41 Alu families, but AluYf4, AluYc5 and AluYf5 families are not listed in Dfam database, so n=38 Alu families are included from evolutionary analysis.

Suppl. Table S5

Class	Family	TAXA CLASSIFICATION			
		Hominoidea	Hominidae	Homininae	H. sapiens
LINE	L1	L1P1, L1PA3, L1PA4, L1PA5	L1PA2	L1HS	
LTR	ERV1	LTR12C, LTR12D, LTR12E, LTR2B, LTR2C,LTR6B, LTR7, HERVE-int	HERV-Fc1_LTR1, HERV-Fc1_LTR2, HERV-Fc1_LTR3, HERV-Fc2_LTR, HERV-Fc2-int	LTR7Y, HERV-Fc1-int	
	ERVK	LTR13, LTR5, LTR5A, MER11B, MER11C, MER11D, MER9a1, MER9a2, HERVK11D-int, HERVK13-int			LTR5_Hs
SINE	Alu		AluYa5,AluYa8,AluYb8,AluYb9, AluYd8, AluYg6, AluYh9, AluYk11, AluYk12, AluYk4		
Other	SVA_A	SVA_A			
	SVA_B		SVA_B		
	SVA_C		SVA_C		
	SVA_D		SVA_D		
	SVA_E				
	SVA_F				SVA_E,SVA_F

Suppl. Table S5. Hominoidea repeats. Table shows the repeat name of n=48 repeats found in *Hominoidea* and descendant clades (from *Hominoidea* to *H.sapiens*). Repetitive element class and family are indicated.

Suppl. Figure S1

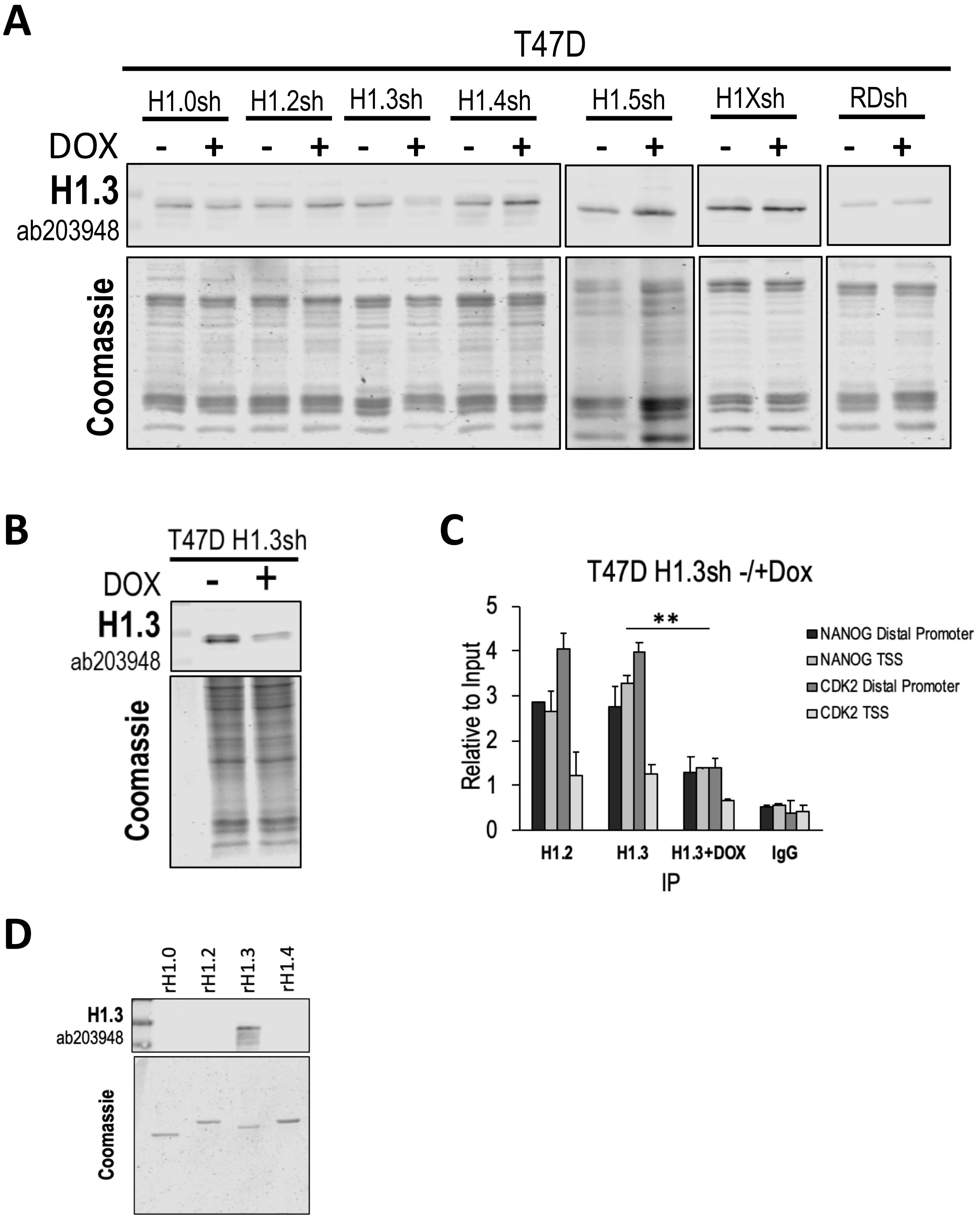

Suppl. Figure S1

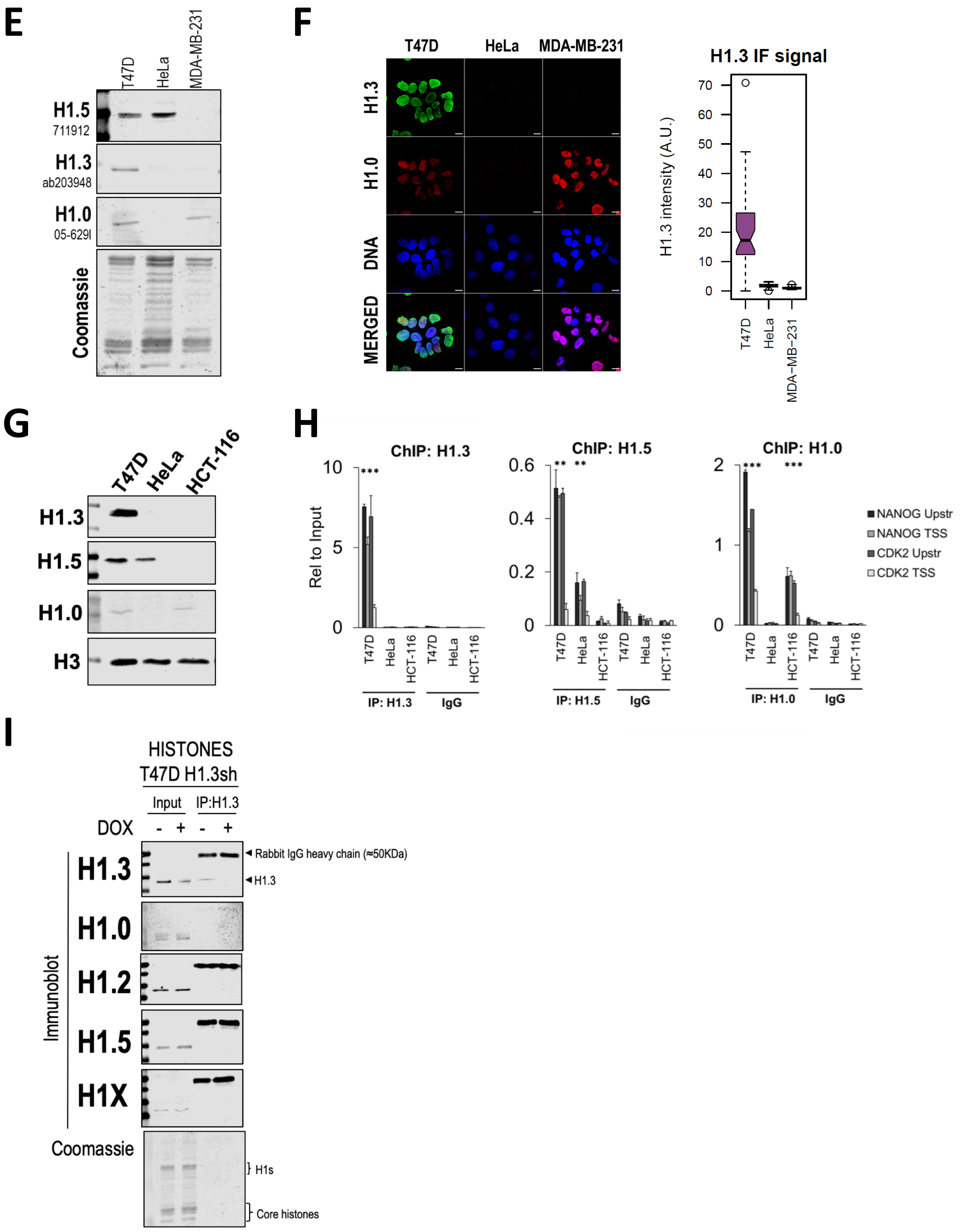

Suppl. Figure S2

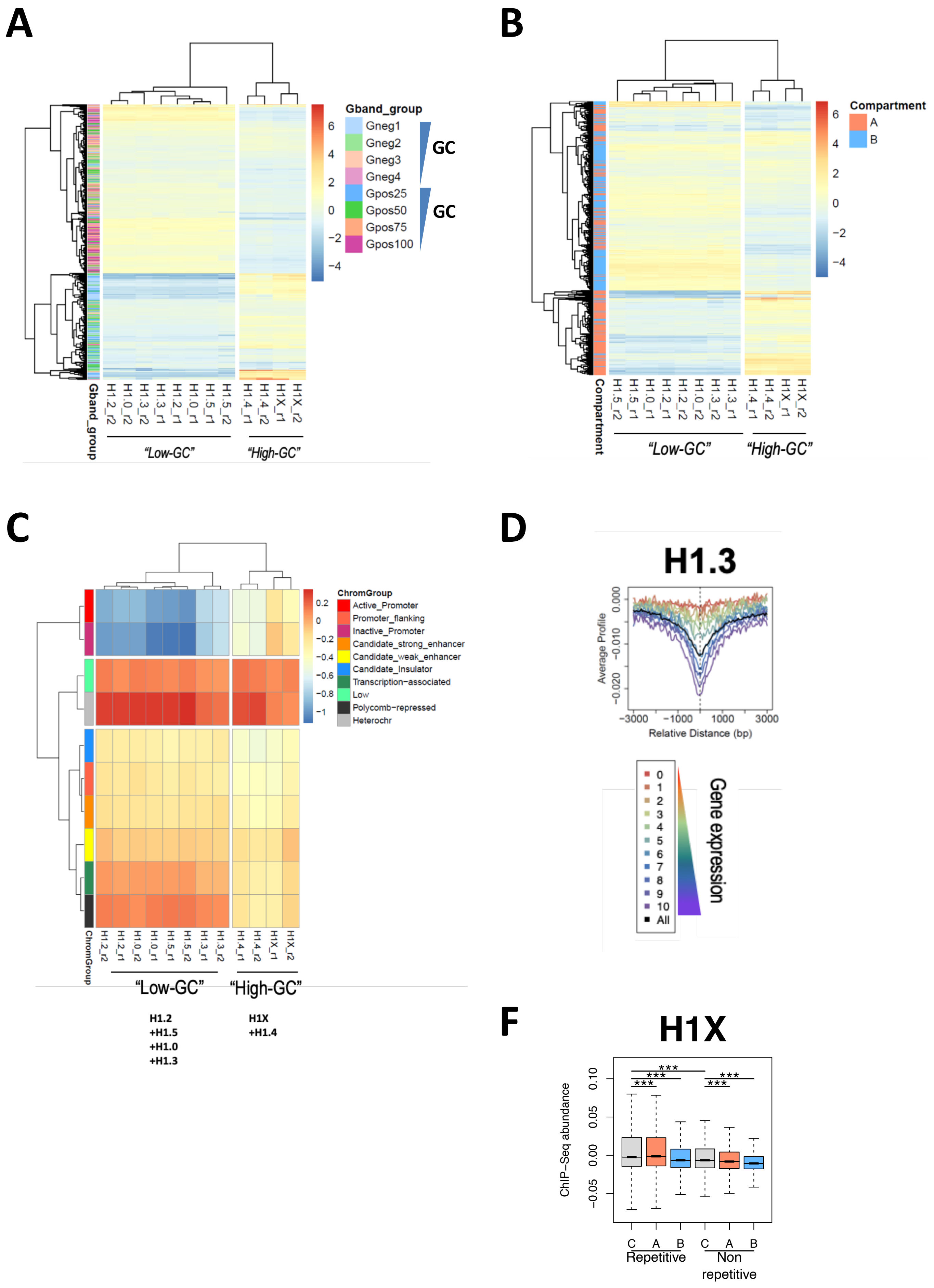

Suppl. Figure S2

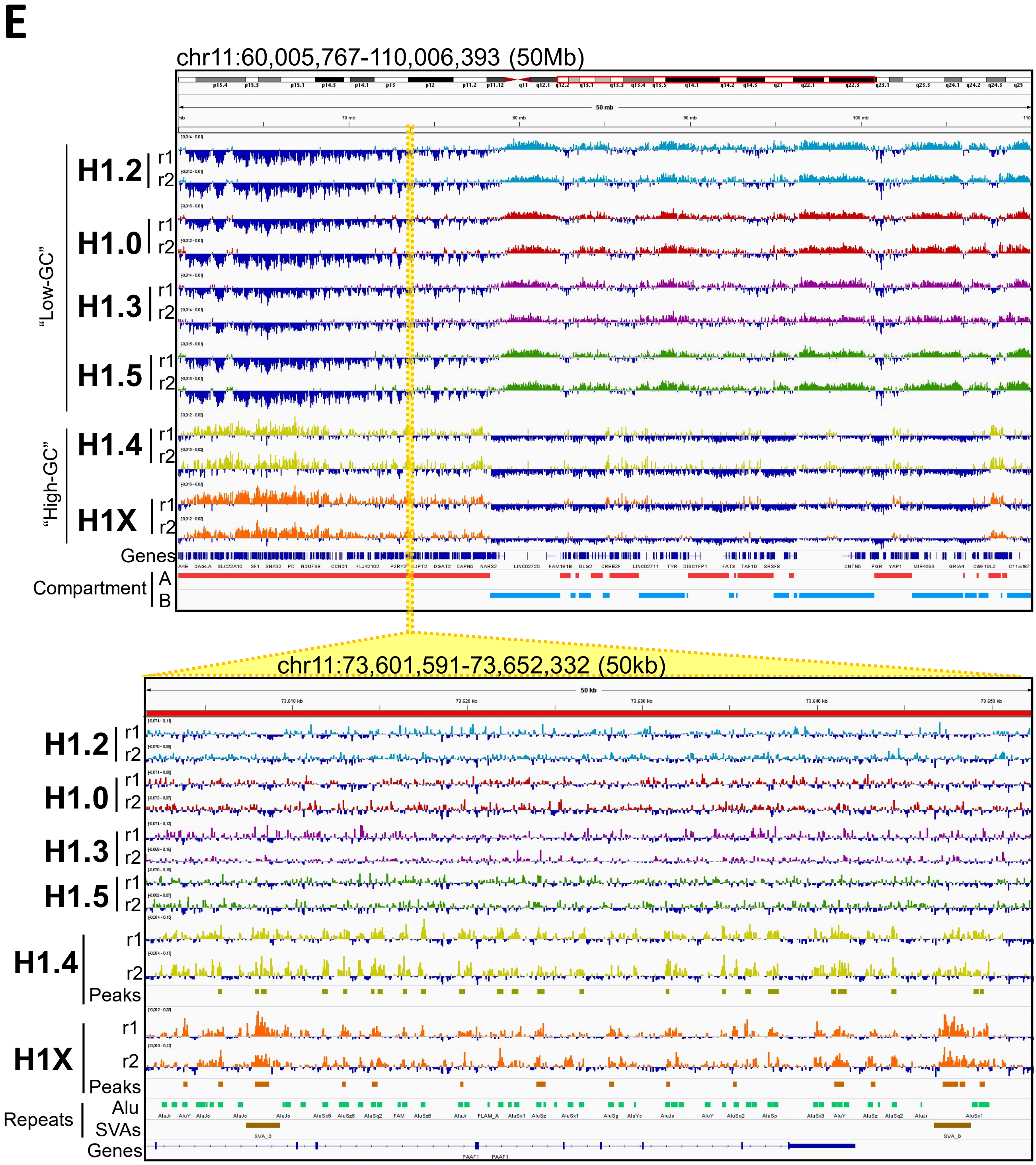

Suppl. Figure S3

A

Classification			Number of repeats		Occupancy		Occupancy (NO BlackList)	
CLASS	FAMILY	REPEAT	N repeats	% repeats	Length (bp)	% Genome	Length (bp)	% Genome
Satellite	telo	2	393		253754	0,00885	138325	0,00482
	Satellite	15	6284		4018893	0,14016	2279792	0,07951
	centr	6	2280		8241150	0,28740	3476426	0,12124
	acro	1	44		30994	0,00108	2418	0,00008
	**4**	**24**	**9001**	**0,203**	**12544791**	**0,43749**	**5896961**	**0,20565**
LINE	L1	122	938568		511513677	17,83870	498926251	17,39972
	L2	4	462077		103527994	3,61047	102432655	3,57227
	CR1	15	60830		10799172	0,37661	10695358	0,37299
	RTE	3	17684		3626086	0,12646	3586861	0,12509
	RTE-BovB	1	655		73851	0,00258	73368	0,00256
	Dong-R4	1	555		119900	0,00418	118783	0,00414
	**6**	**146**	**1480369**	**33,370**	**629660680**	**21,95900**	**615833276**	**21,47678**
DNA	hAT-Charlie	68	251950		44762514	1,56106	44219359	1,54212
	hAT	9	15431		2174159	0,07582	2143765	0,07476
	hAT-Tip100	24	30241		6584658	0,22964	6525062	0,22756
	TcMar-Tigger	52	102878		33883625	1,18167	33341399	1,16276
	MuDR	5	1979		696734	0,02430	680962	0,02375
	hAT-Blackjack	8	19591		3399907	0,11857	3372472	0,11761
	TcMar	6	5360		938657	0,03274	930989	0,03247
	TcMar-Tc2	8	8103		1661836	0,05796	1635037	0,05702
	PiggyBac	6	2355		540852	0,01886	532513	0,01857
	DNA	12	2737		344189	0,01200	341483	0,01191
	TcMar-Mariner	5	16266		2813921	0,09813	2772550	0,09669
	Merlin	1	57		17783	0,00062	17783	0,00062
	**12**	**204**	**456948**	**10,300**	**97818835**	**3,41137**	**96513374**	**3,36584**
SINE	MIR	4	590625		83759060	2,92104	82933991	2,89227
	Alu	41	1175329		305569843	10,65655	300059507	10,46438
	SINE	1	959		160316	0,00559	159406	0,00556
	Deu	2	1265		177915	0,00620	177432	0,00619
	**4**	**48**	**1768178**	**39,857**	**389667134**	**13,58938**	**383330336**	**13,36839**
LTR	ERVL-MaLR	80	343675		110506219	3,85383	108651828	3,78916
	ERVL	102	159792		56393139	1,96667	55496857	1,93542
	LTR	3	2201		472590	0,01648	466173	0,01626
	ERV1	260	172893		83321108	2,90577	81393769	2,83855
	ERVK	37	10490		8832106	0,30801	8392195	0,29267
	Gypsy	19	18580		3742640	0,13052	3708924	0,12935
	ERV	3	579		191030	0,00666	185454	0,00647
	**7**	**504**	**708210**	**15,964**	**263458832**	**9,18795**	**258295200**	**9,00787**
Unknown	MamRep605	1	4412		865506	0,03018	851354	0,02969
	UCON16	1	37		5092	0,00018	5092	0,00018
	MamRep564	1	793		112386	0,00392	111216	0,00388
	UCON22	1	35		4369	0,00015	4369	0,00015
	UCON20	1	44		9450	0,00033	9450	0,00033
	UCON11	1	45		8979	0,00031	8979	0,00031
	UCON4	1	123		16928	0,00059	16853	0,00059
	UCON19	1	28		4860	0,00017	4860	0,00017
	UCON26	1	206		29477	0,00103	29477	0,00103
	UCON12A	1	26		3850	0,00013	3850	0,00013
	UCON5	1	102		13839	0,00048	13839	0,00048
	UCON31	1	110		17655	0,00062	17655	0,00062
	UCON6	1	79		12062	0,00042	12062	0,00042
	UCON28b	1	77		14371	0,00050	14371	0,00050
	Eulor4	1	31		6453	0,00023	6453	0,00023
	UCON8	1	99		15187	0,00053	15187	0,00053
	UCON18	1	18		3067	0,00011	3067	0,00011
	UCON25	1	43		3674	0,00013	3232	0,00011
	UCON28c	1	65		12833	0,00045	12833	0,00045
	UCON2	1	119		15858	0,00055	15858	0,00055
	UCON15	1	44		6855	0,00024	6855	0,00024
	UCON27	1	103		22142	0,00077	22142	0,00077
	UCON10	1	64		11988	0,00042	11988	0,00042
	UCON28a	1	129		24345	0,00085	24345	0,00085
	UCON1	1	29		3319	0,00012	3319	0,00012
	UCON9	1	48		8502	0,00030	8502	0,00030
	UCON24	1	22		3323	0,00012	3323	0,00012
	UCON17	1	33		6112	0,00021	6112	0,00021
	UCON12	1	45		5793	0,00020	5793	0,00020
	**29**	**29**	**7009**	**0,158**	**1268275**	**0,04423**	**1252436**	**0,04368**
Other	SVA_D	1	1373		1818950	0,06343	1778047	0,06201
	SVA_E	1	235		236198	0,00824	233208	0,00813
	SVA_F	1	995		760014	0,02650	744568	0,02597
	SVA_C	1	281		358428	0,01250	354853	0,01238
	SVA_B	1	466		554267	0,01933	552905	0,01928
	SVA_A	1	258		280084	0,00977	271333	0,00946
	**6**	**6**	**3608**	**0,081**	**4007941**	**0,13977**	**3934914**	**0,13723**
**RNA**	**RNA**	**1**	**718**	**0,016**	**118030**	**0,00412**	**116545**	**0,00406**
**RC**	**Helitron**	**4**	**2231**	**0,050**	**450351**	**0,01571**	**446546**	**0,01557**
**9**	**70**	**966**	**4436272**	**100**	**1398994869**	**48,78902**	**1365619588**	**47,62508**

C

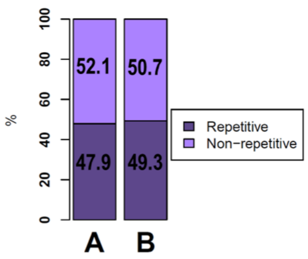

D

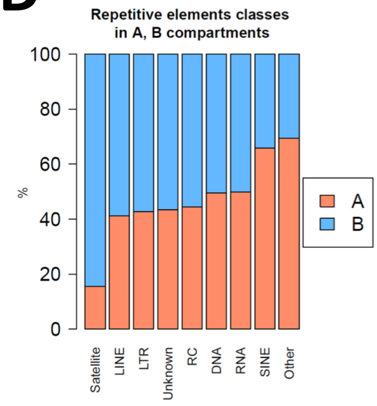

E

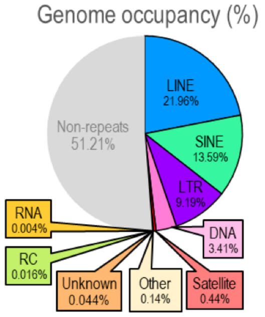

B

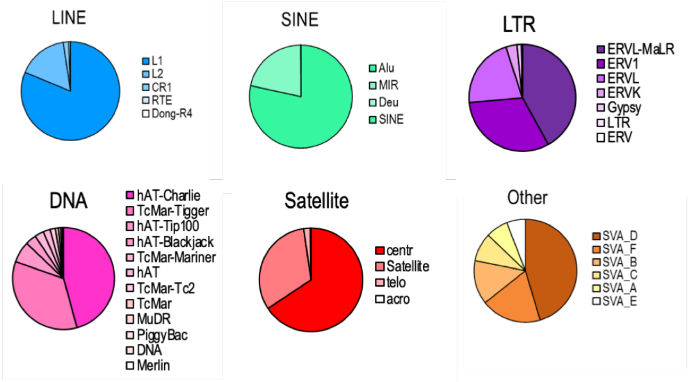

Suppl. Figure S4

A

T47D ChIP-Seq	MACS2 NARROW PEAKS (n)
**H1X**	180399
**H1.4**	339617
**H1.2**	114
**H1.3**	206
**H1.5**	91
**H1.0**	53

B

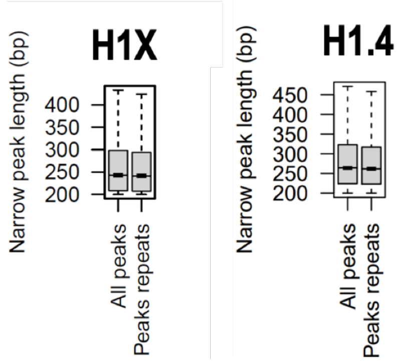

C

REPEAT CLASSIFICATION			H1X narrow peaks				H1.4 narrow peaks			
**Class**	**Family**	**copies (n)**	H1X narrow peaks (n)	H1X narrow peaks (1 per repeat)	% H1X narrow peaks	% family with H1X narrow peak	H1.4 narrow peaks (n)	H1.4 narrow peaks (1 per repeat)	% H1.4 narrow peaks	% family with H1.4 narrow peak
**DNA**	DNA	2701								
	hAT	15177								
	hAT-Blackjack	19382								
	hAT-Charlie	247696	13	13	0.008	0.005	9	9	0.003	0.004
	hAT-Tip100	29864	2	2	0.001	0.007	1	1	0.000	0.003
	PiggyBac	2295								
	TcMar	5305								
	TcMar-Mariner	15995								
	TcMar-Tc2	7966								
	TcMar-Tigger	100712	8	8	0.005	0.008	10	10	0.003	0.010
	Merlin	54								
	MuDR	1928								
	**12**	**449075**								
**LINE**	CR1	59938	2	2	0.001	0.003				
	Dong-R4	551								
	L1	911361	26445	19135	16.376	2.100	43351	29134	13.667	3.197
	L2	456087	35	35	0.022	0.008	8	8	0.003	0.002
	RTE	17416	1	1	0.001	0.006				
	RTE-BovB	651								
	**6**	**1446004**								
**LTR**	ERV	553								
	ERV1	164965	1165	1114	0.721	0.675	525	514	0.166	0.312
	ERVK	9500	1	1	0.001	0.011				
	ERVL	156220	21	21	0.013	0.013	21	21	0.007	0.013
	ERVL-MaLR	335366	1499	1496	0.928	0.446	2487	2487	0.784	0.742
	Gypsy	18370	1	1	0.001	0.005				
	LTR	2158								
	**7**	**687132**								
**Satellite**	Satellite	2074	1	1	0.001	0.048			0.000	0.000
	acro	6			0.000	0.000				
	centr	1168	51	43	0.032	3.682	94	74	0.030	6.336
	telo	212	1	1	0.001	0.472				
	**4**	**3460**								
**SINE**	Alu	1144739	129513	129510	80.200	11.313	269121	269115	84.846	23.509
	Deu	1259	1	1	0.001	0.079				
	MIR	583523	26	26	0.016	0.004	6	6	0.002	0.001
	SINE	951	1	1	0.001	0.105	1	1	0.000	0.105
	**4**	**1730472**								
**Other**	SVA_A	247	184	146	0.114	59.109	130	108	0.041	43.725
	SVA_B	461	388	323	0.240	70.065	246	203	0.078	44.035
	SVA_C	277	275	227	0.170	81.949	162	141	0.051	50.903
	SVA_D	1341	1319	1064	0.817	79.344	701	575	0.221	42.878
	SVA_E	233	140	122	0.087	52.361	60	54	0.019	23.176
	SVA_F	967	394	346	0.244	35.781	254	237	0.080	24.509
	**6**	3526								
**RC**	**Helitron**	2193								
**RNA**	**RNA**	708								
			**161487**	**153640**	**100**		**317187**	**302698**	**100**	

Suppl. Figure S4

D

T47D ChIP-Seq	DROMPA PEAKS (n)
**H1X**	200561
**H1.4**	400490
**H1.2**	14676
**H1.3**	6072
**H1.5**	23732
**H1.0**	20337

E

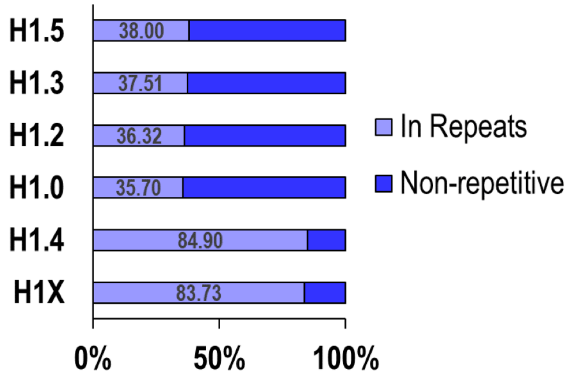

F

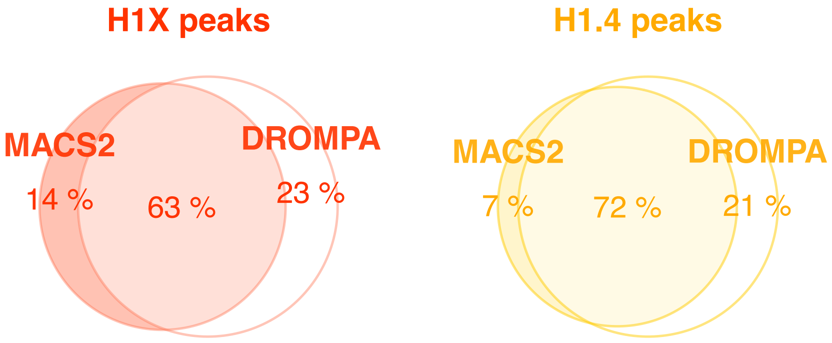

G

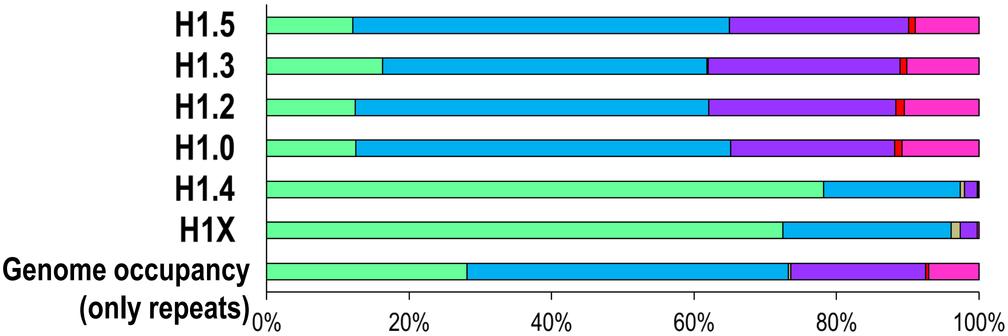

	Genome Occupancy (only Repeats)	H1X	H1.4	H1.0	H1.2	H1.3	H1.5
SINE	28.07	72.48	78.17	12.51	12.41	16.21	12.08
LINE	45.10	23.57	19.17	52.47	49.46	45.42	52.72
SVAs	0.29	1.31	0.63	0.00	0.02	0.19	0.01
LTR	18.91	2.37	1.82	22.98	26.22	26.87	25.13
Satellite	0.43	0.06	0.10	1.04	1.18	0.98	0.93
DNA	7.07	0.22	0.12	10.78	10.44	10.09	8.92

Suppl. Figure S5

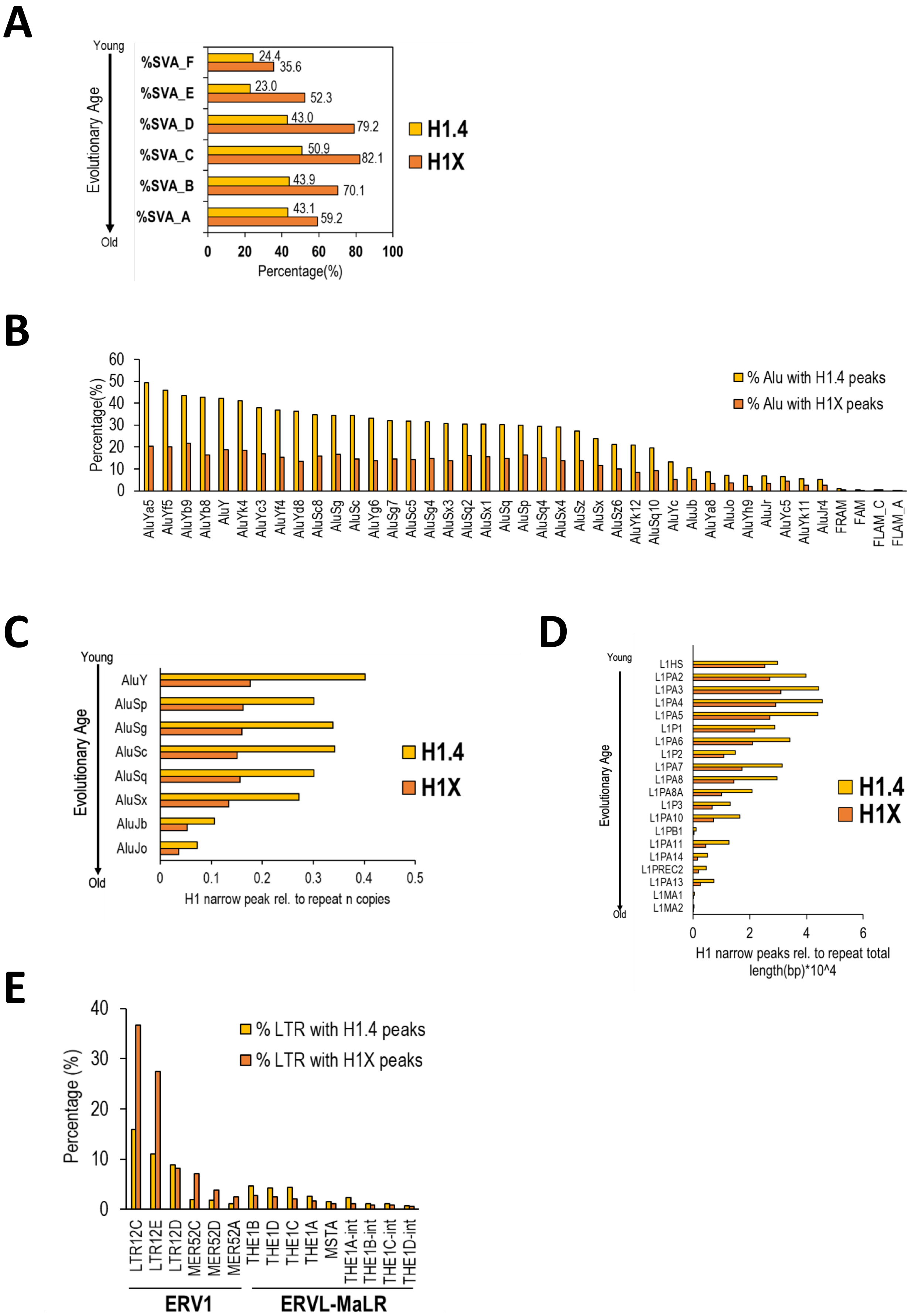

Suppl. Figure S5

F

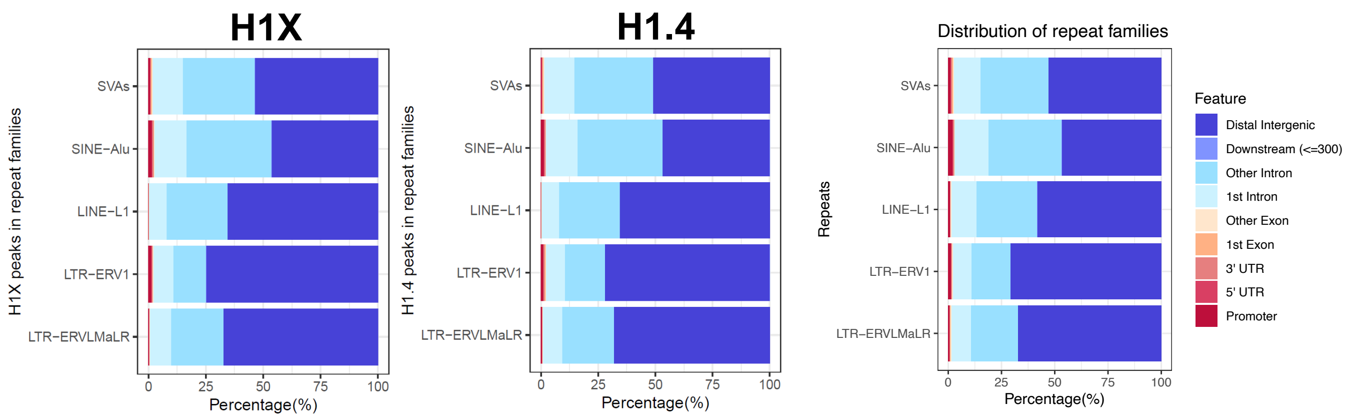

	SVAs			SINE-Alu			LINE-L1			LTR-ERV1			LTR-ERVLMaLR		
	H1X	H1.4	All	H1X	H1.4	All	H1X	H1.4	All	H1X	H1.4	All	H1X	H1.4	All
Distal intergenic	53.52	50.93	52.81	46.15	46.68	46.55	65.39	65.35	58.20	74.76	71.81	70.70	67.18	67.91	67.16
Downstream (<=300)	0.04	0.00	0.11	0.13	0.13	0.14	0.01	0.01	0.05	0.09	0.19	0.07	0.07	0.00	0.06
Other intron	31.33	34.32	31.74	37.16	37.21	34.43	26.62	26.61	28.56	14.25	17.52	18.29	22.75	22.68	21.99
1st intron	13.22	13.33	12.65	13.92	13.65	15.77	7.66	7.67	11.69	8.58	8.19	8.53	9.27	8.52	9.33
Other exon	0.41	0.39	0.34	0.24	0.23	0.23	0.08	0.10	0.24	0.43	0.19	0.50	0.13	0.12	0.38
1st exon	0.37	0.26	0.60	0.08	0.07	0.08	0.04	0.04	0.08	0.09	0.38	0.13	0.07	0.08	0.09
3' UTR	0.26	0.26	0.45	0.69	0.63	0.66	0.06	0.07	0.32	0.26	0.57	0.29	0.13	0.16	0.32
5' UTR	0.07	0.13	0.09	0.07	0.05	0.06	0.00	0.00	0.04	0.26	0.00	0.07	0.07	0.04	0.05
Promoter	0.78	0.39	1.22	1.56	1.35	2.09	0.14	0.15	0.83	1.29	1.14	1.41	0.33	0.48	0.63

G

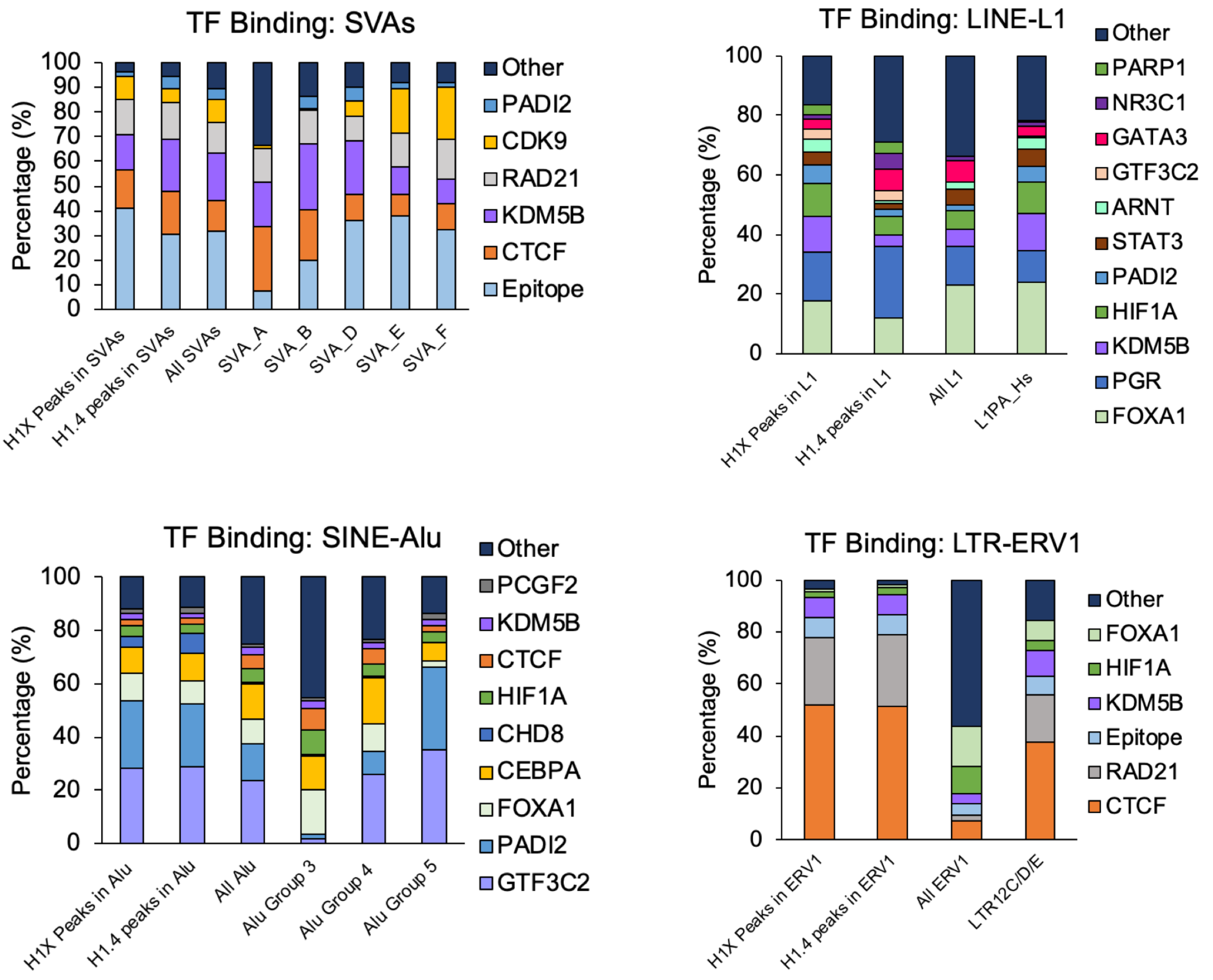

Suppl. Figure S6

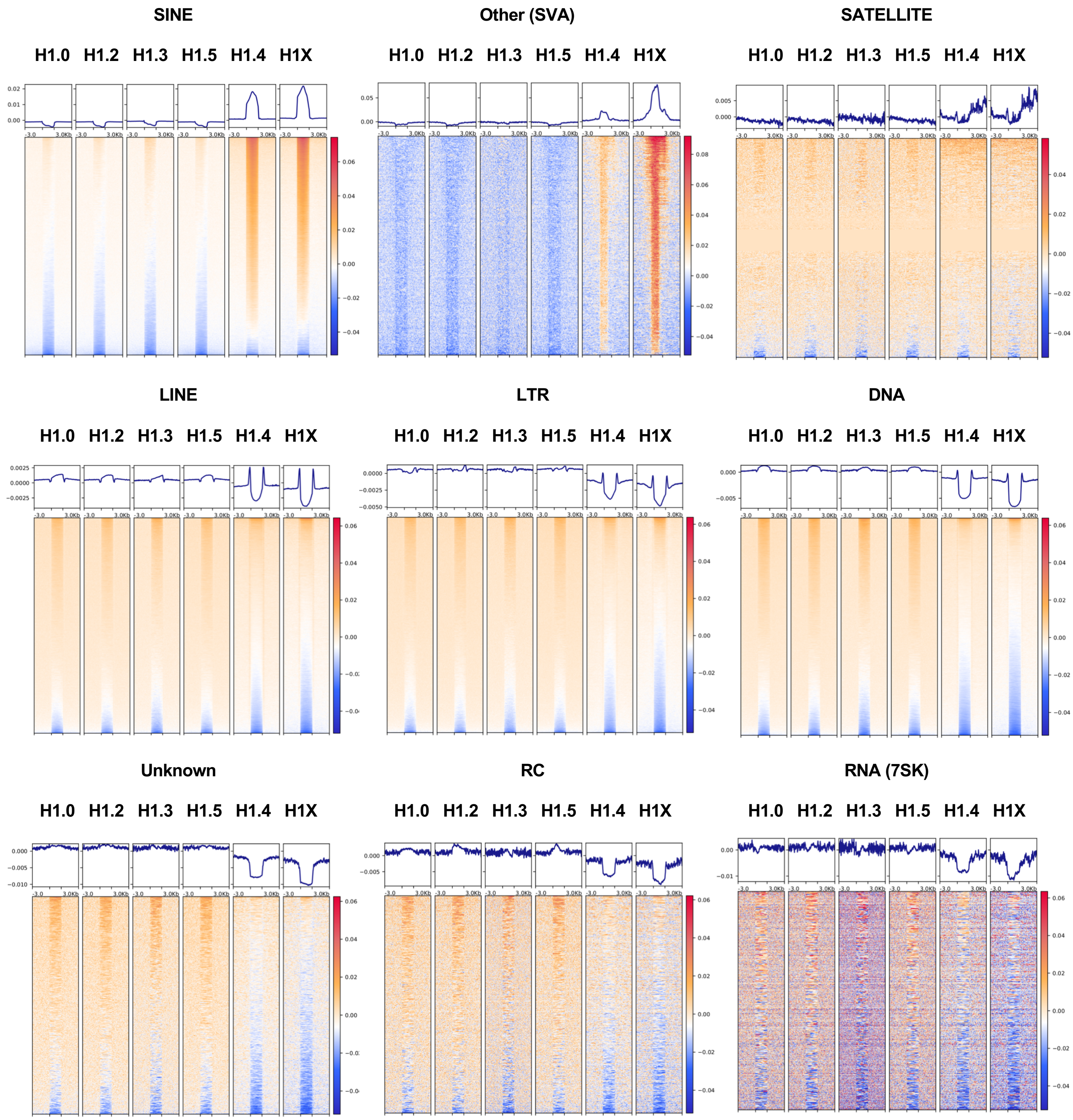

Suppl. Figure S7

A

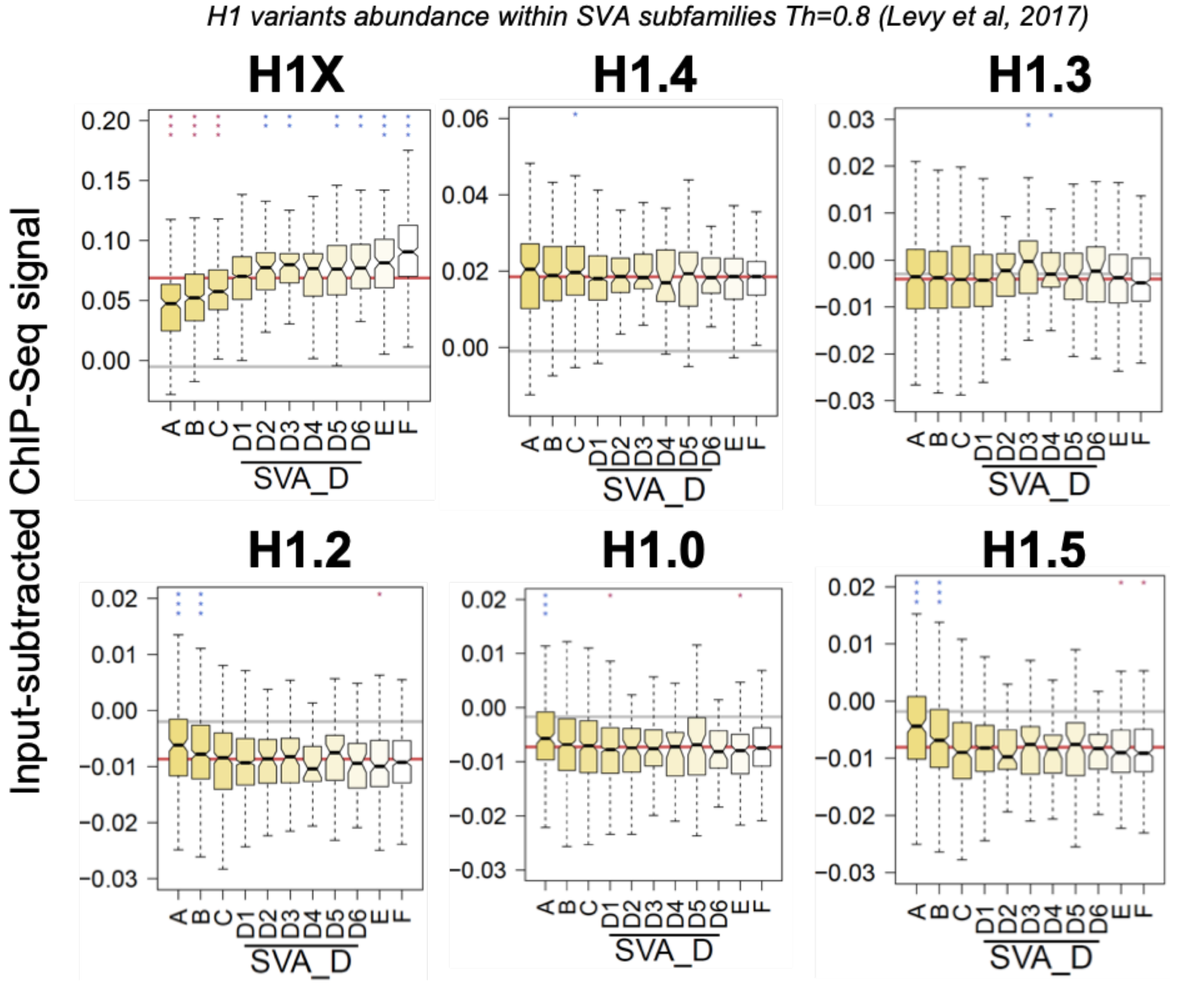

B

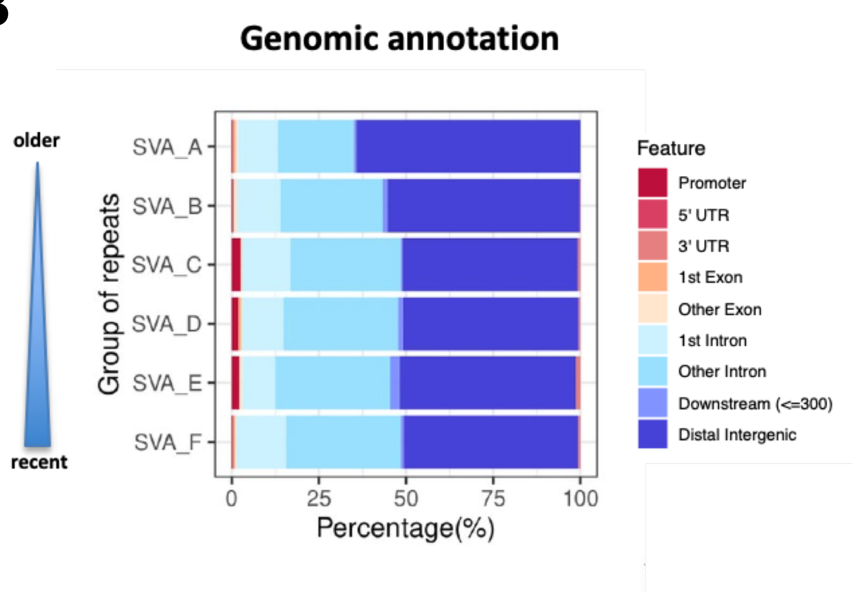

Suppl. Figure S8

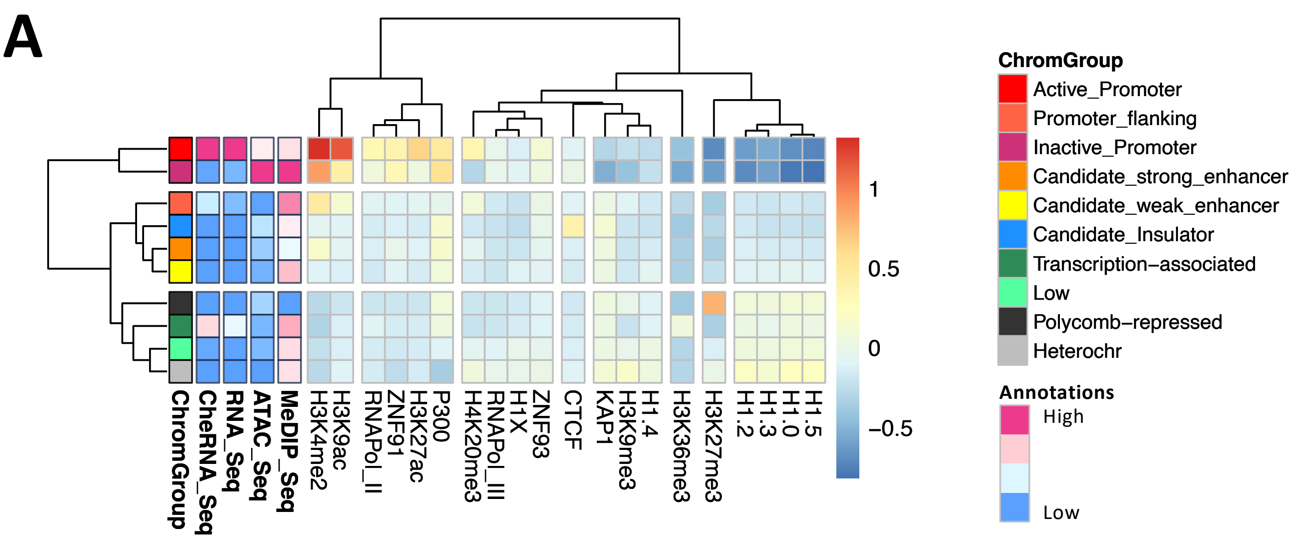

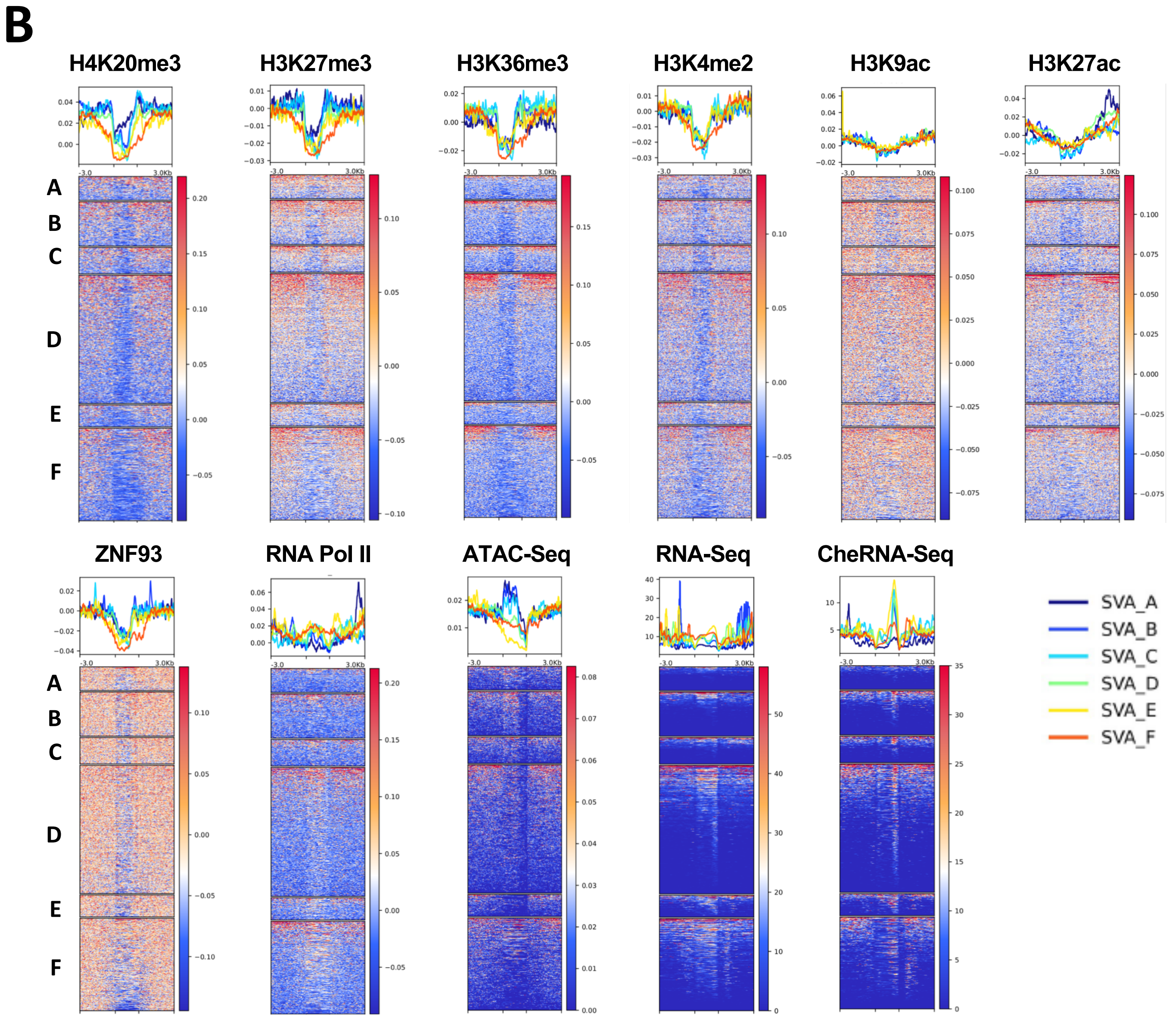

Suppl. Figure S8

C

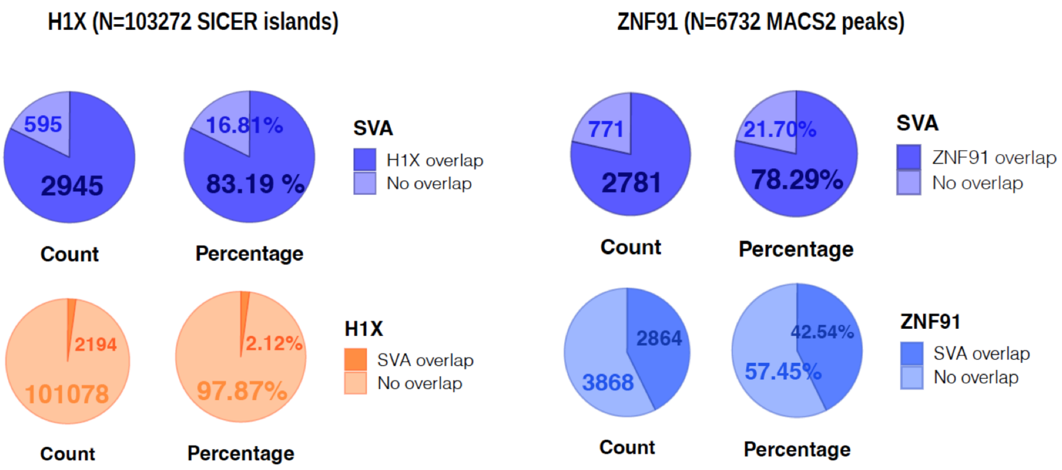

D

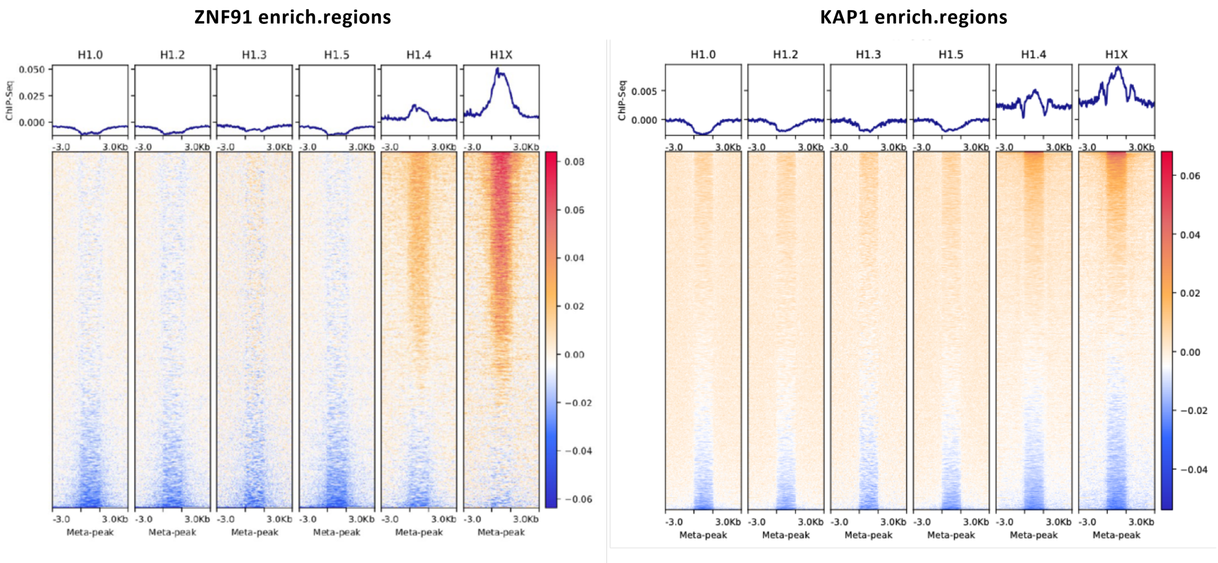

Suppl. Figure S9

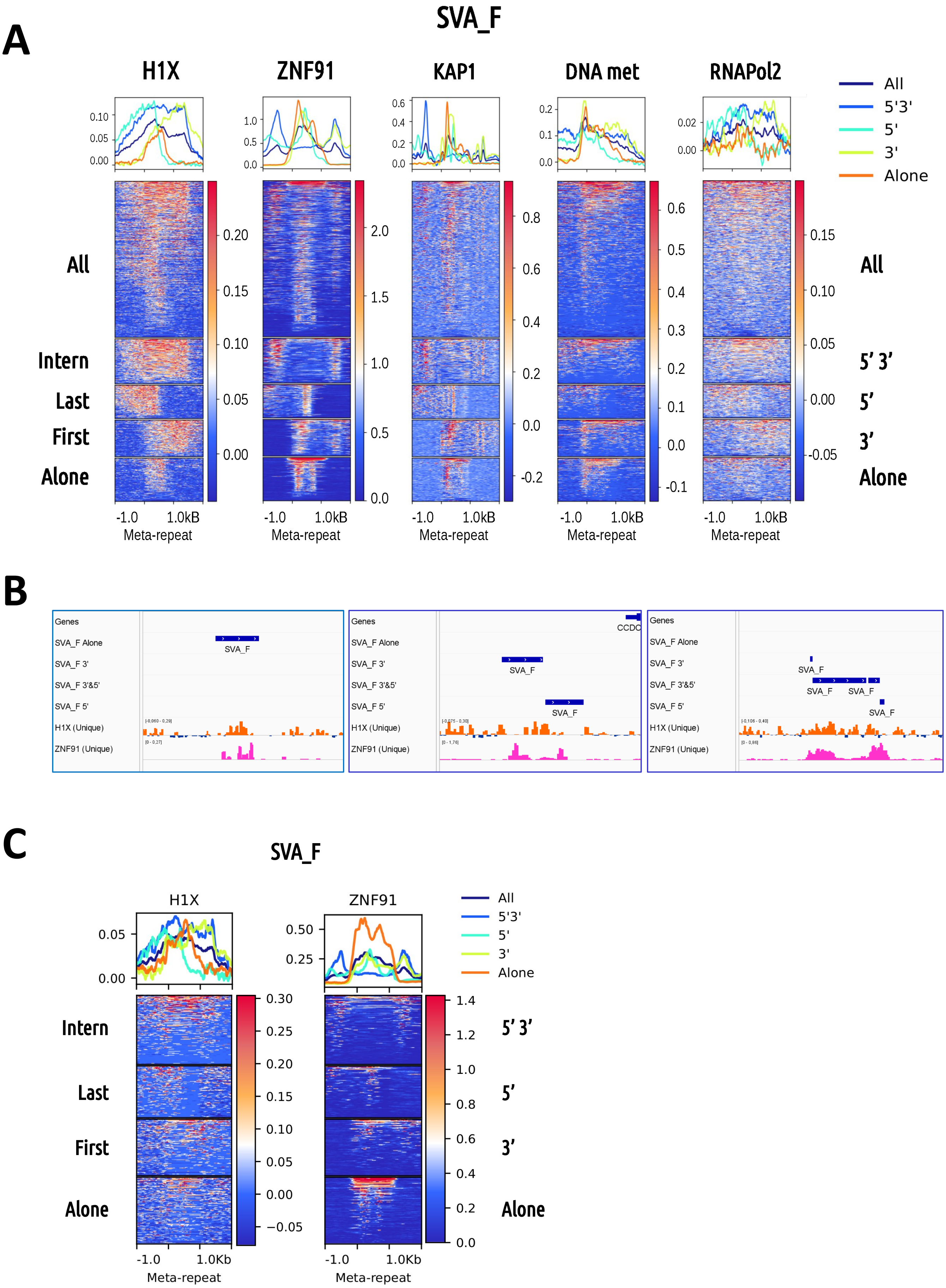

Suppl. Figure S9

D

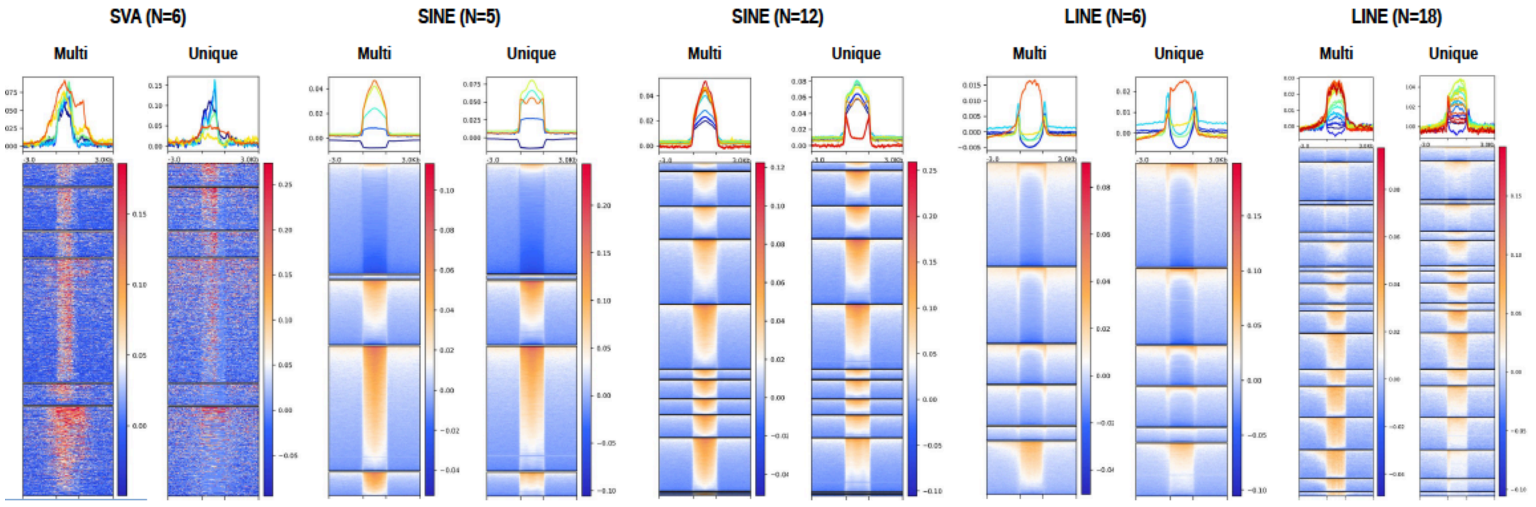

E

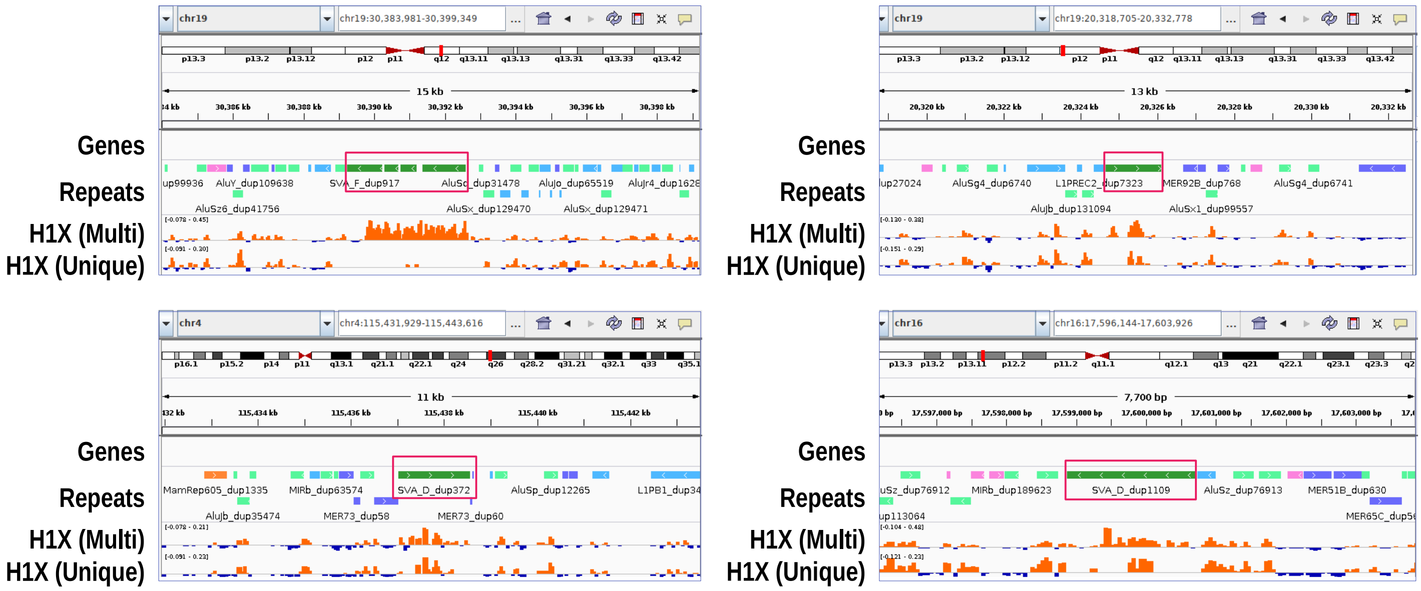

Suppl. Figure S10

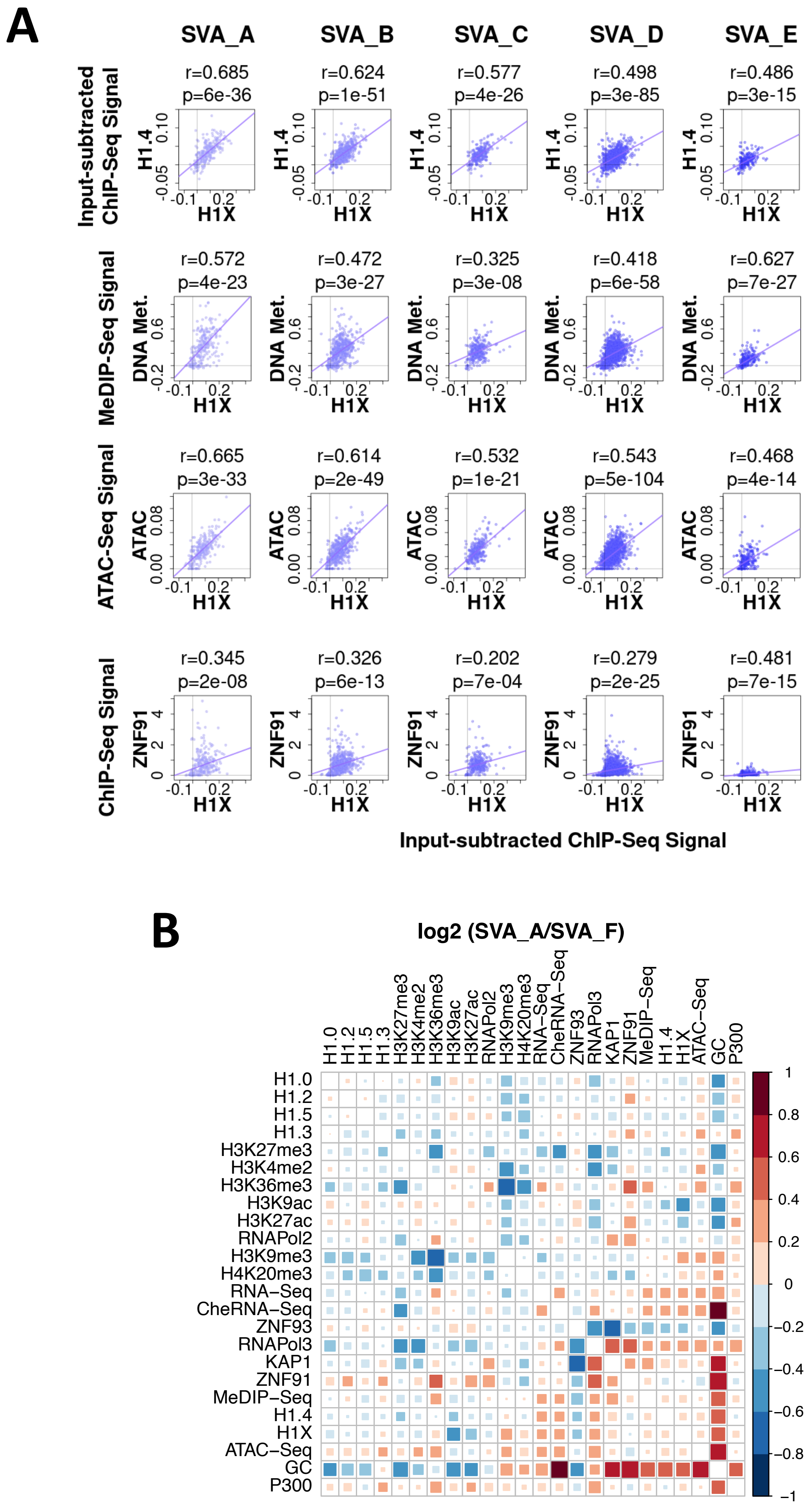

Suppl. Figure S11

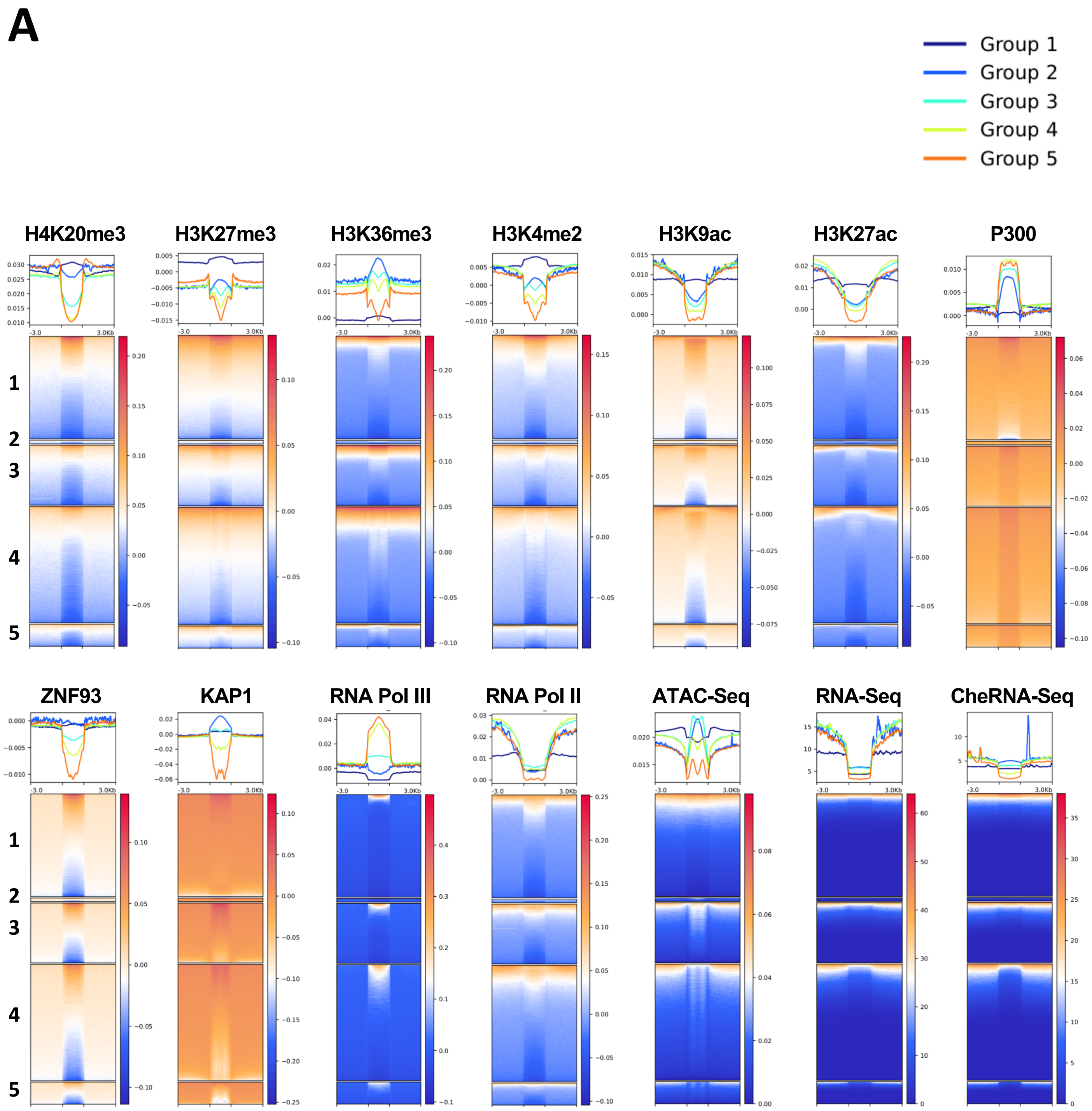

Suppl. Figure S11

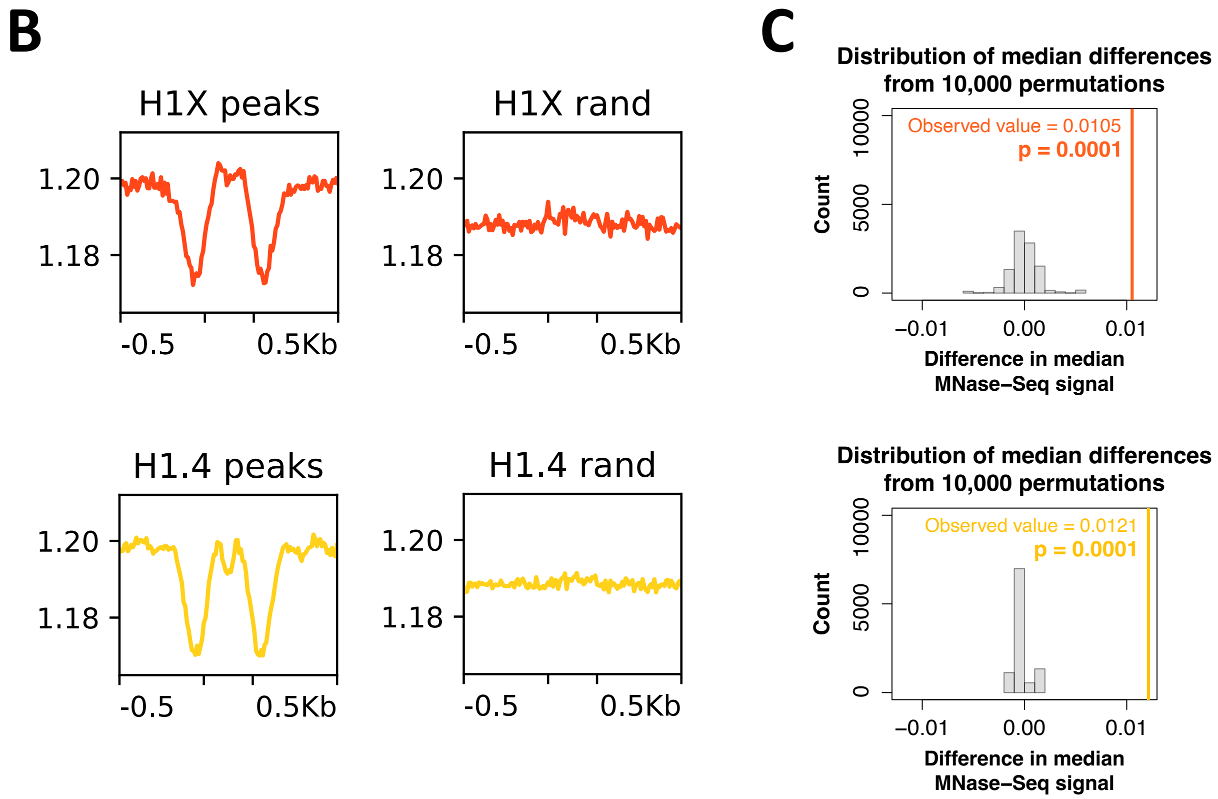

Suppl. Figure S12

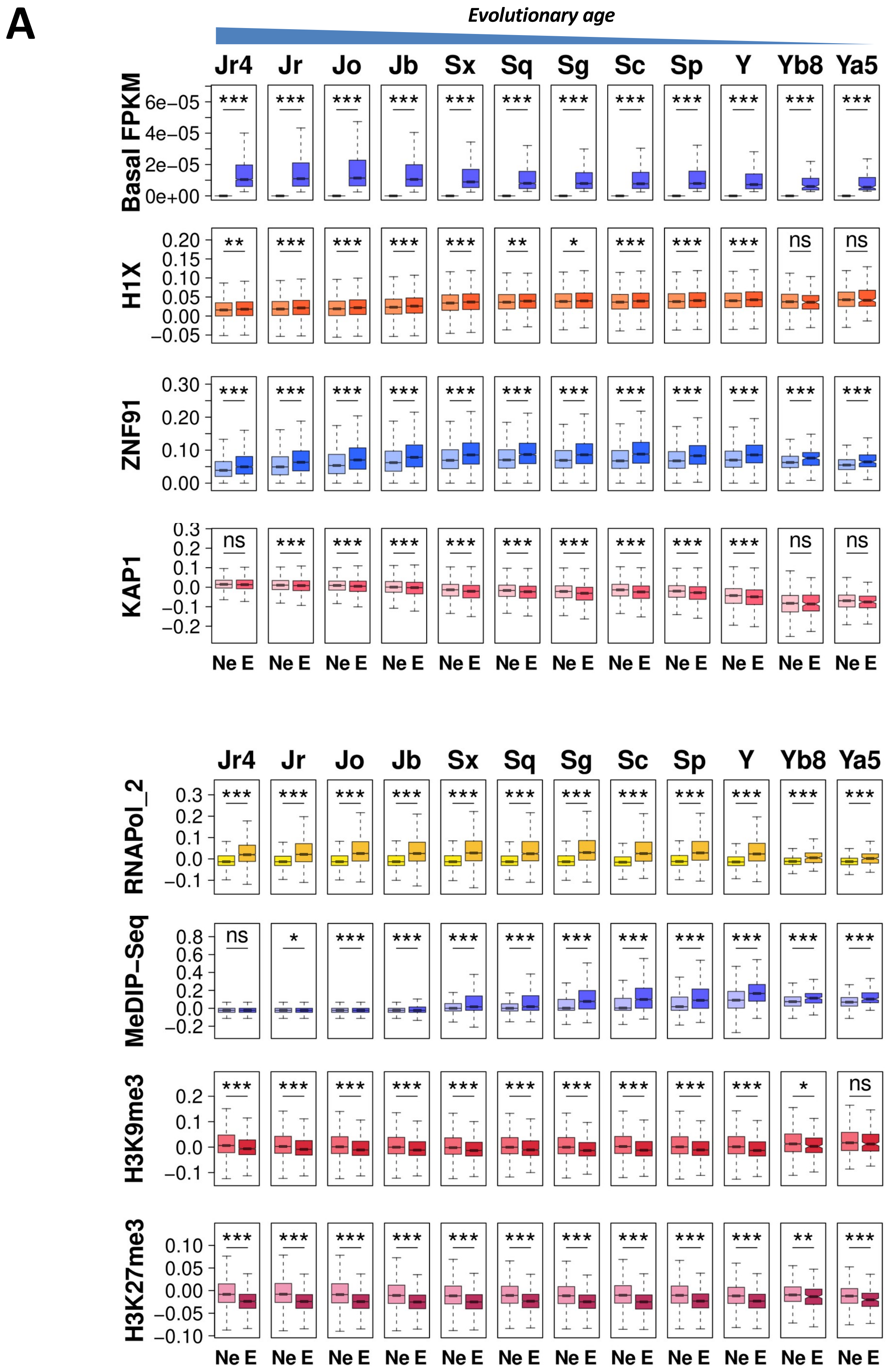

Suppl. Figure S12

B

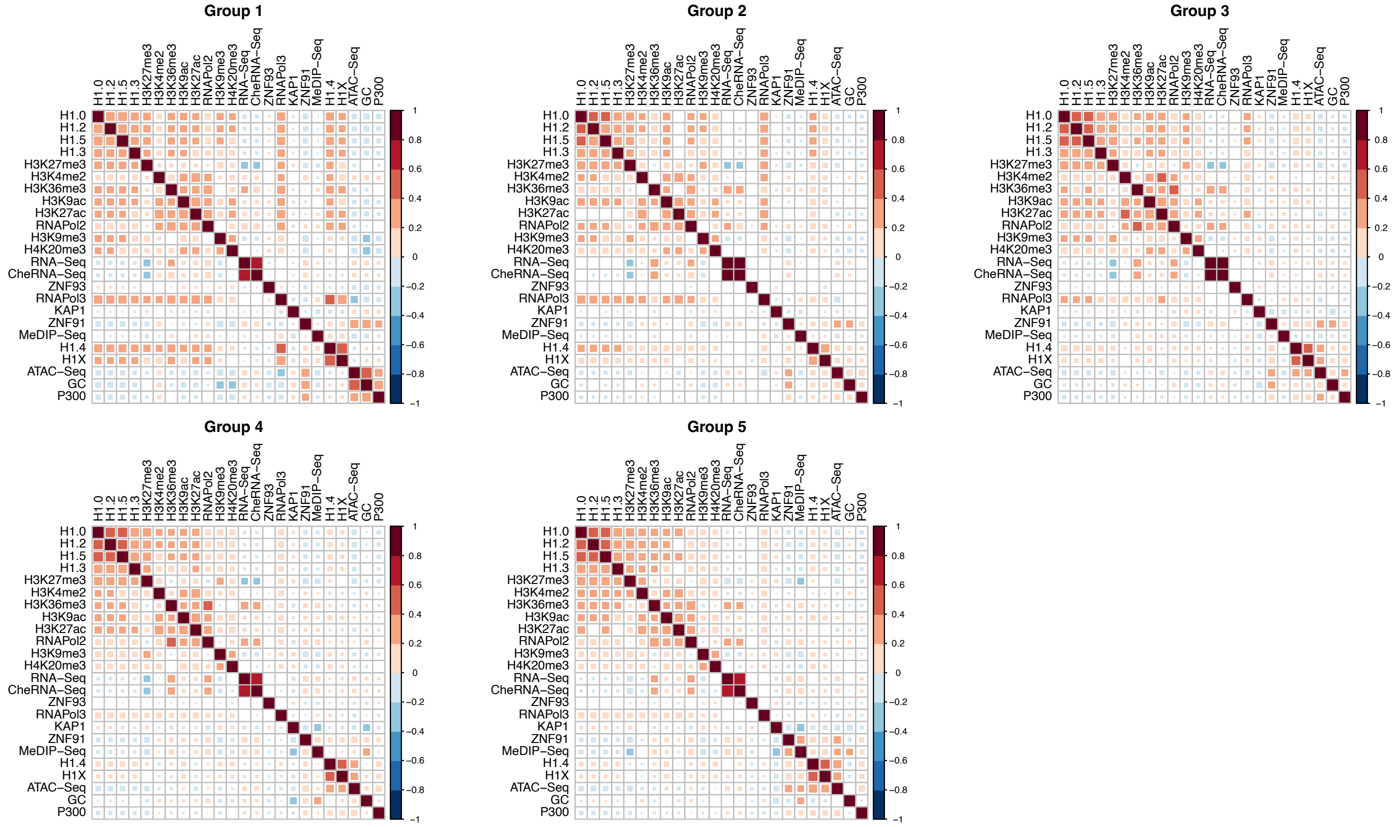

C

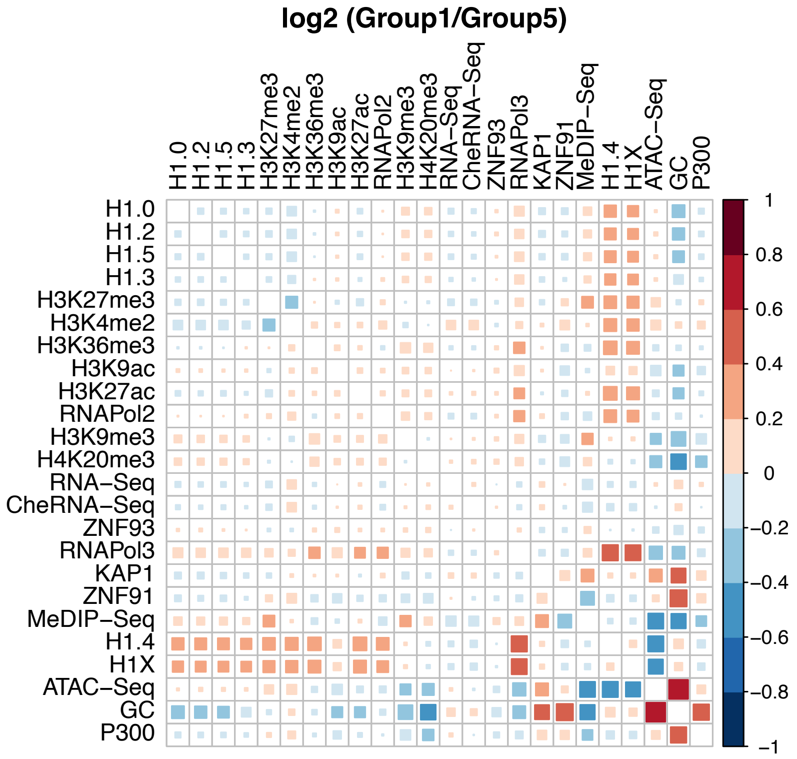

Suppl. Figure S13

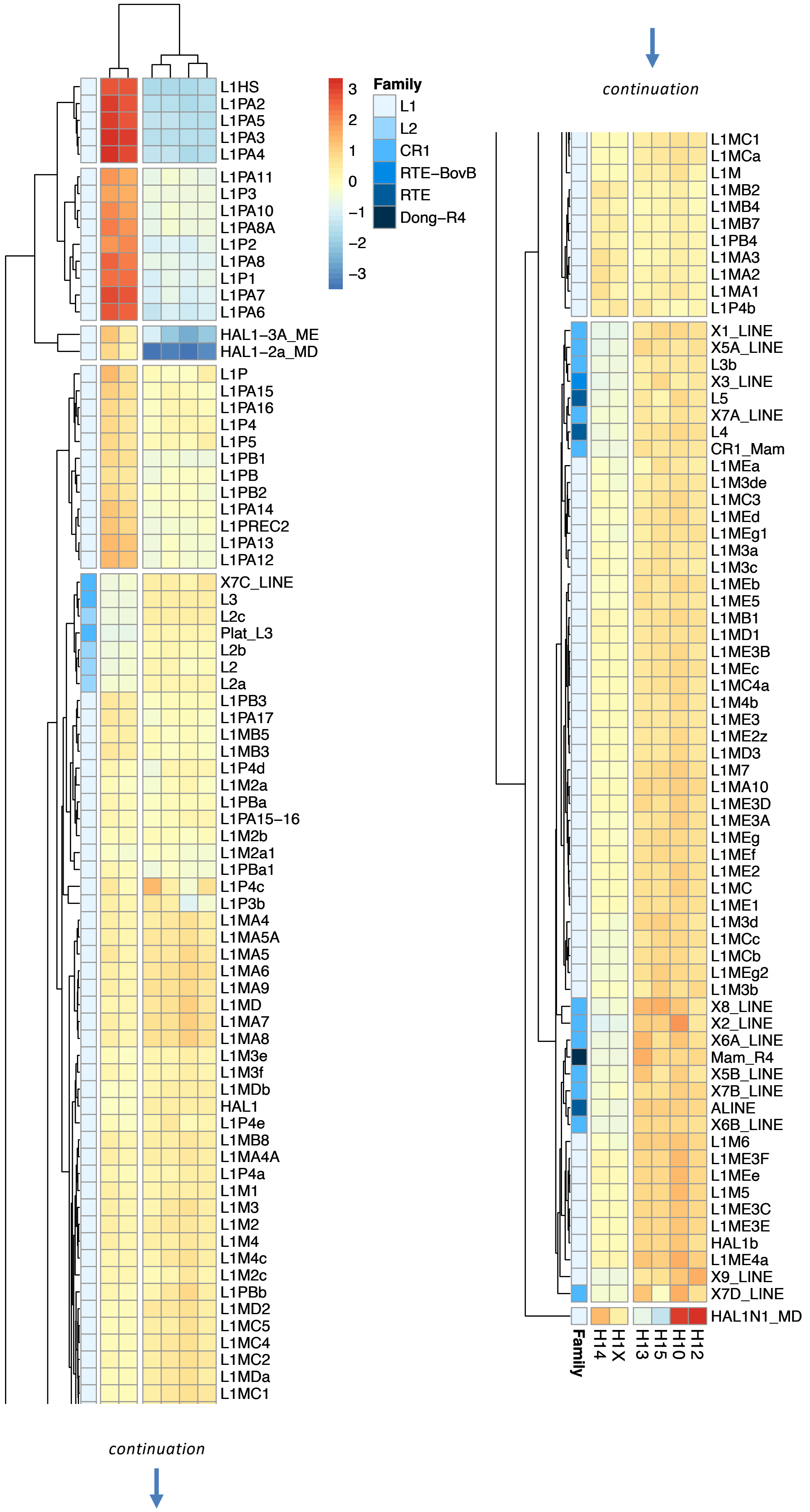

Suppl. Figure S14

A

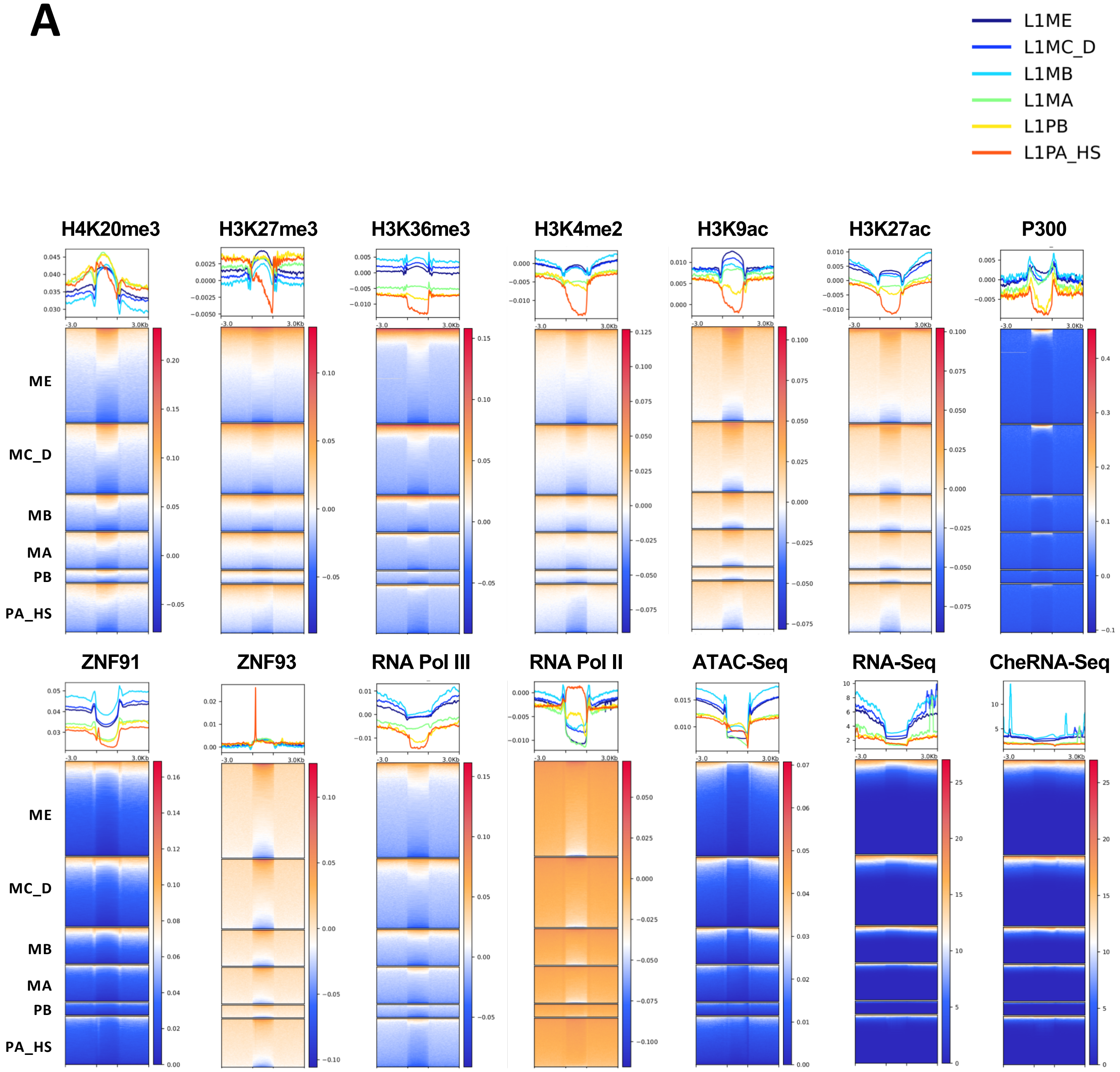

Suppl. Figure S14

B

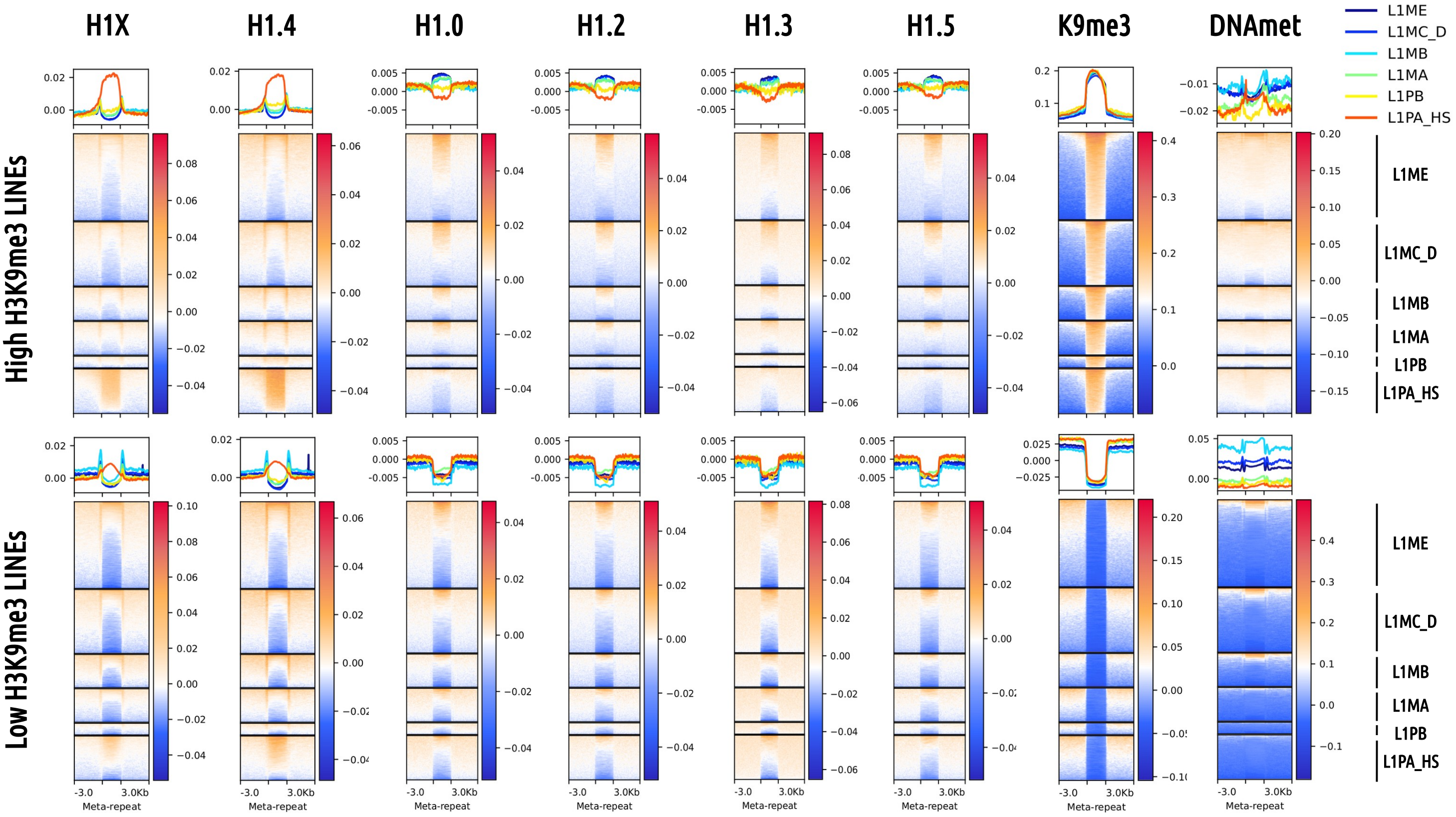

Suppl. Figure S14

C

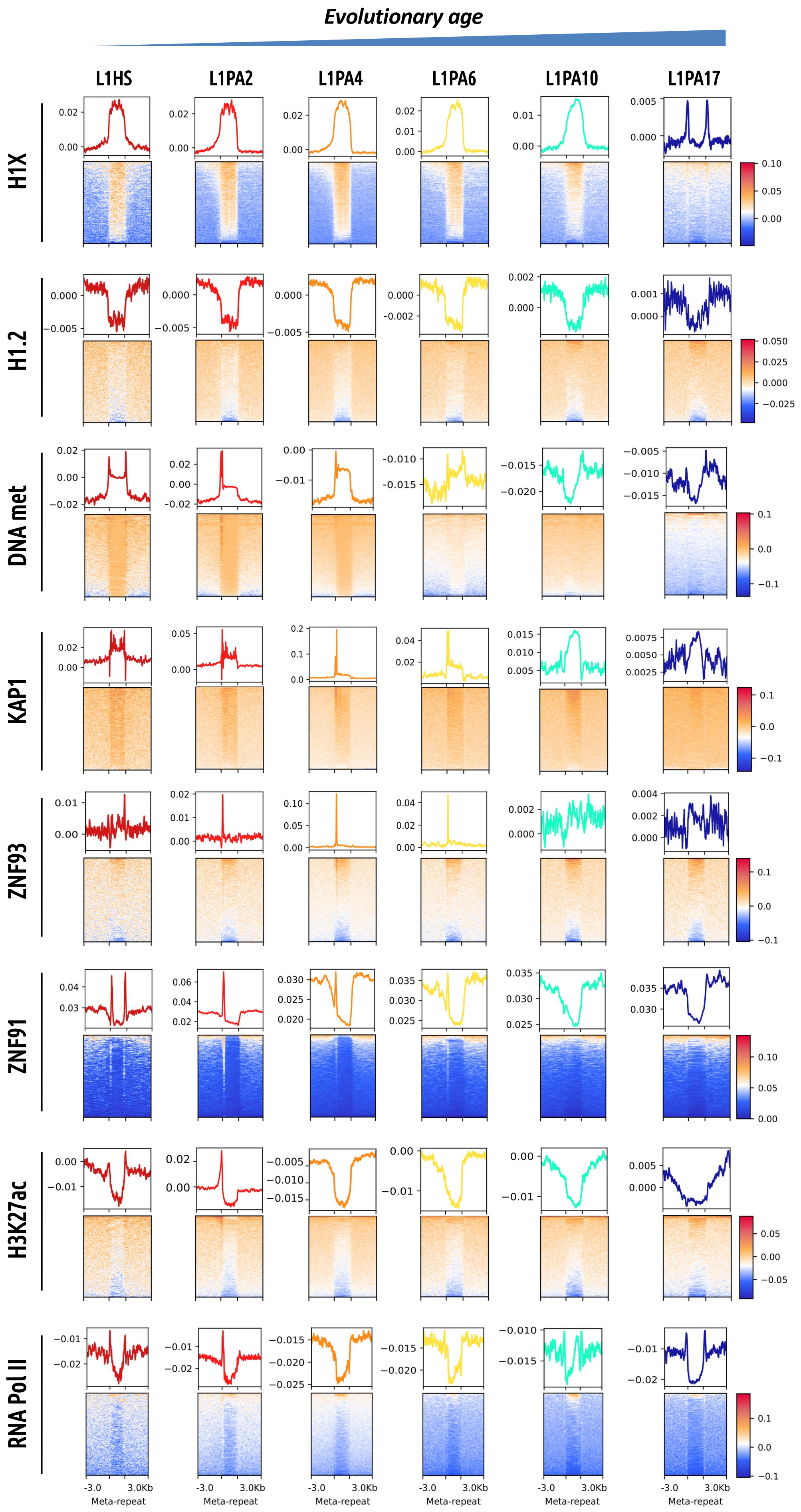

Suppl. Figure S15

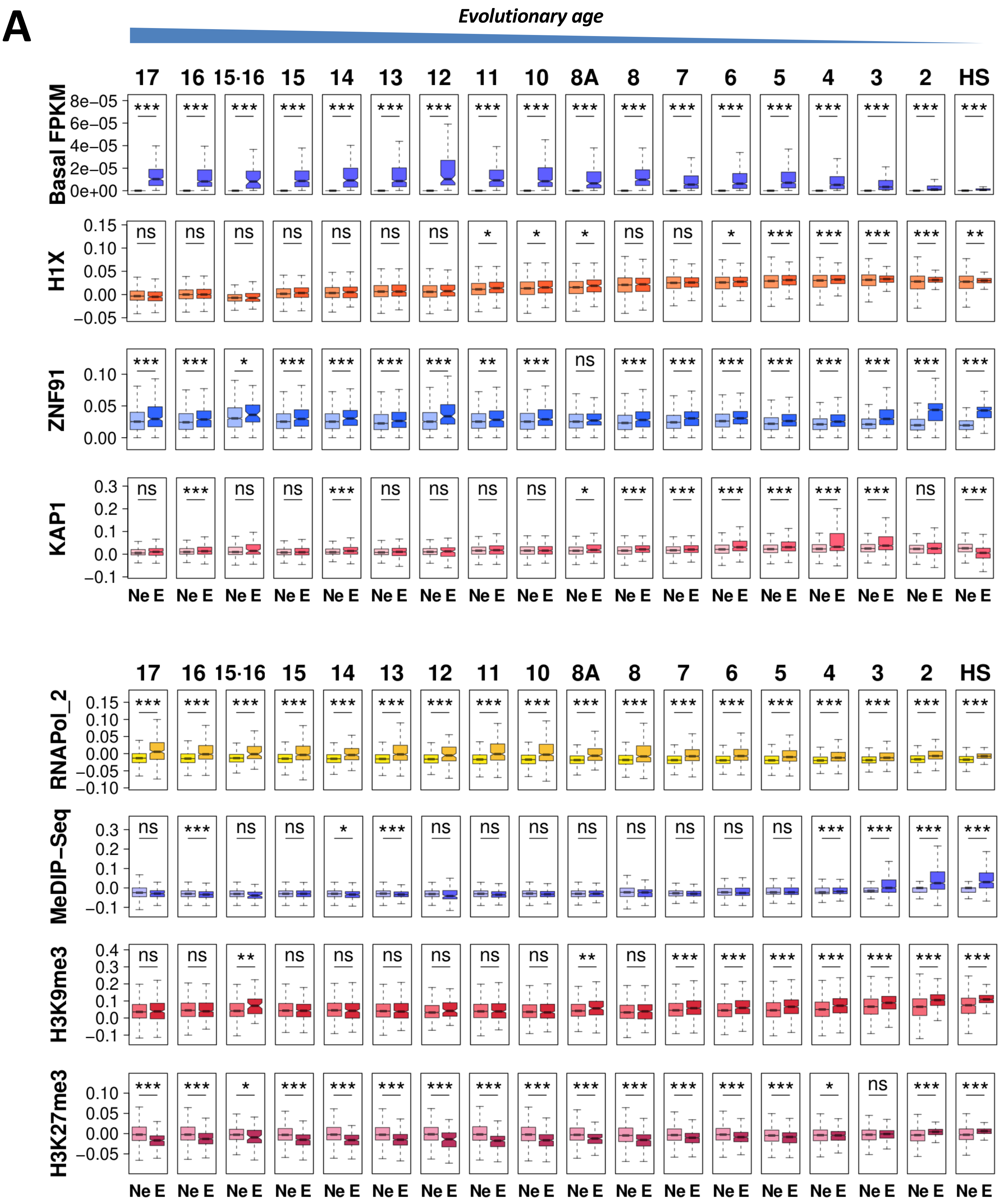

Suppl. Figure S15

B

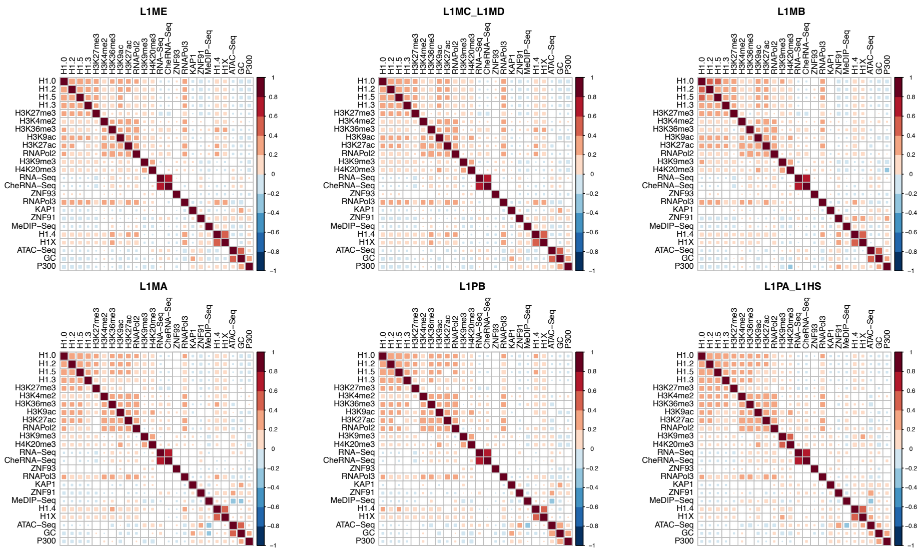

C

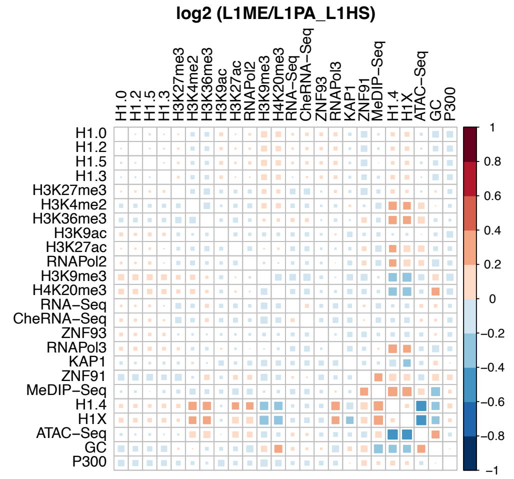

D

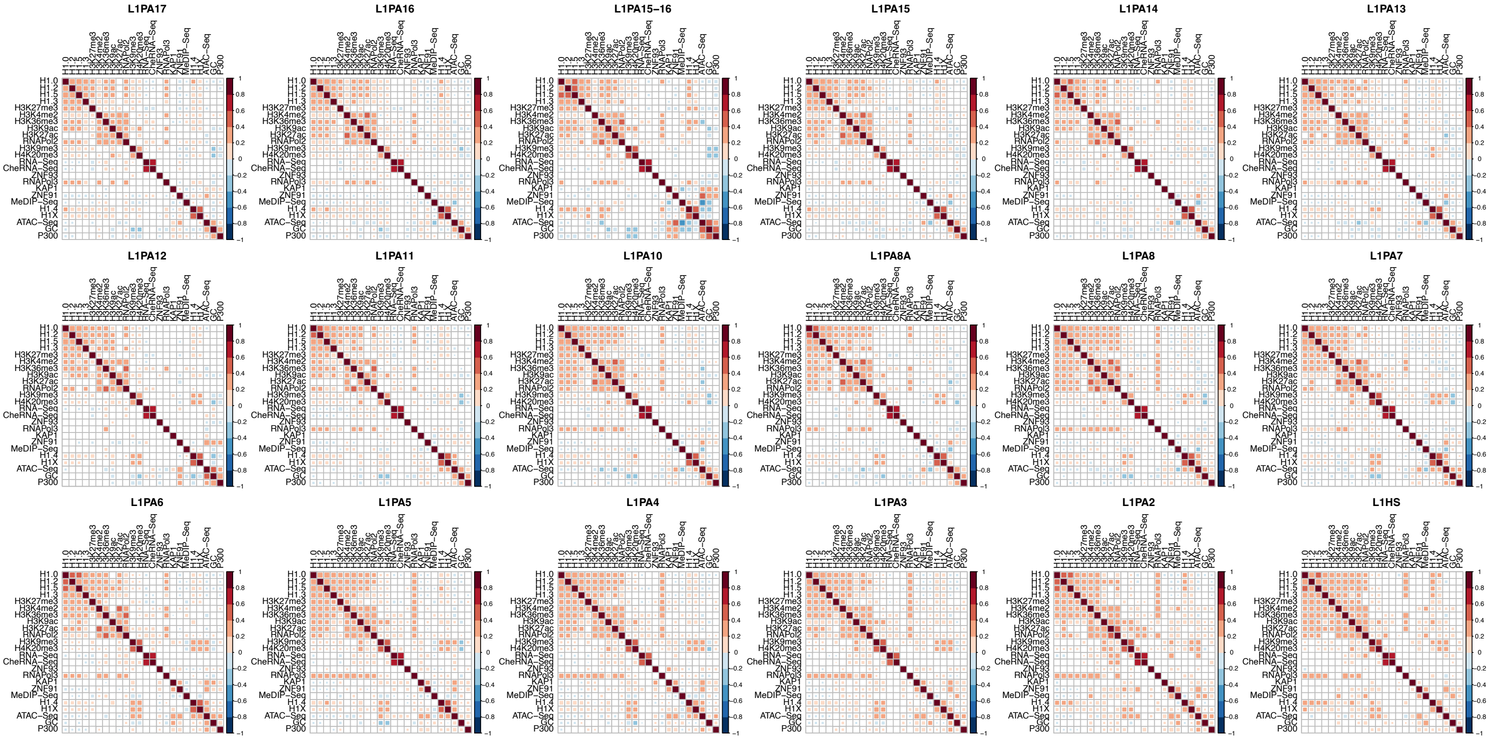

Suppl. Figure S16

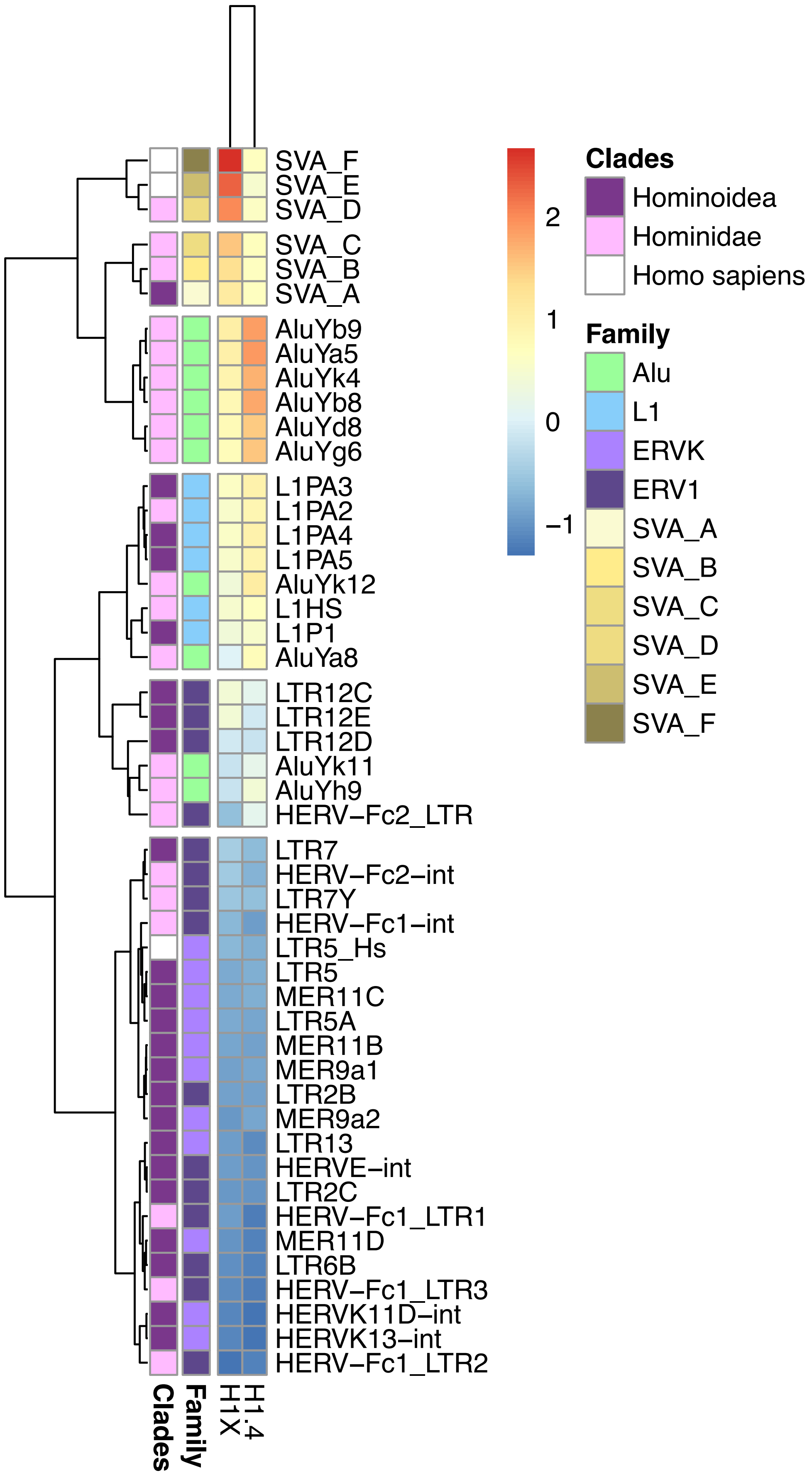

Suppl. Figure S17

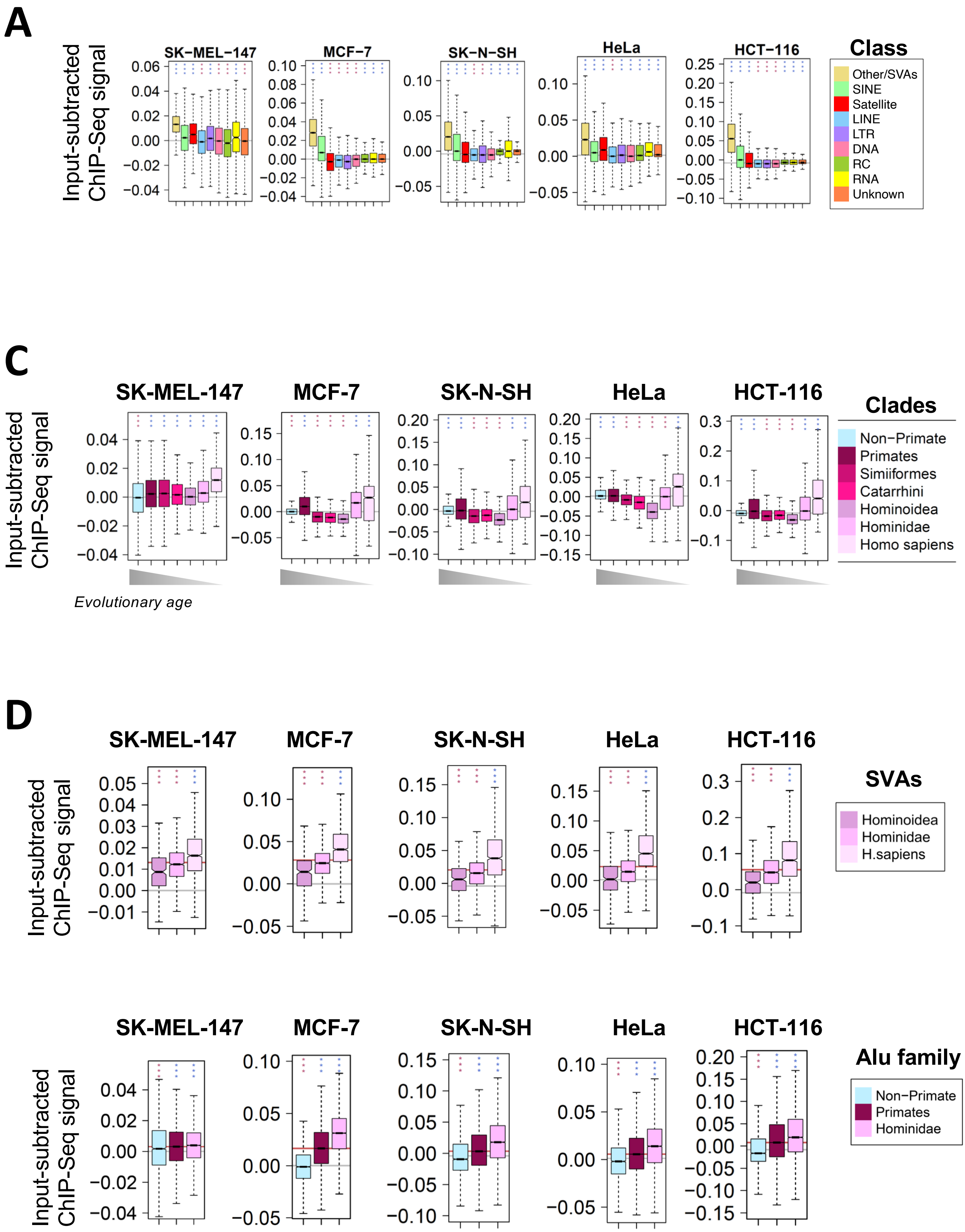

Suppl. Figure S17

B

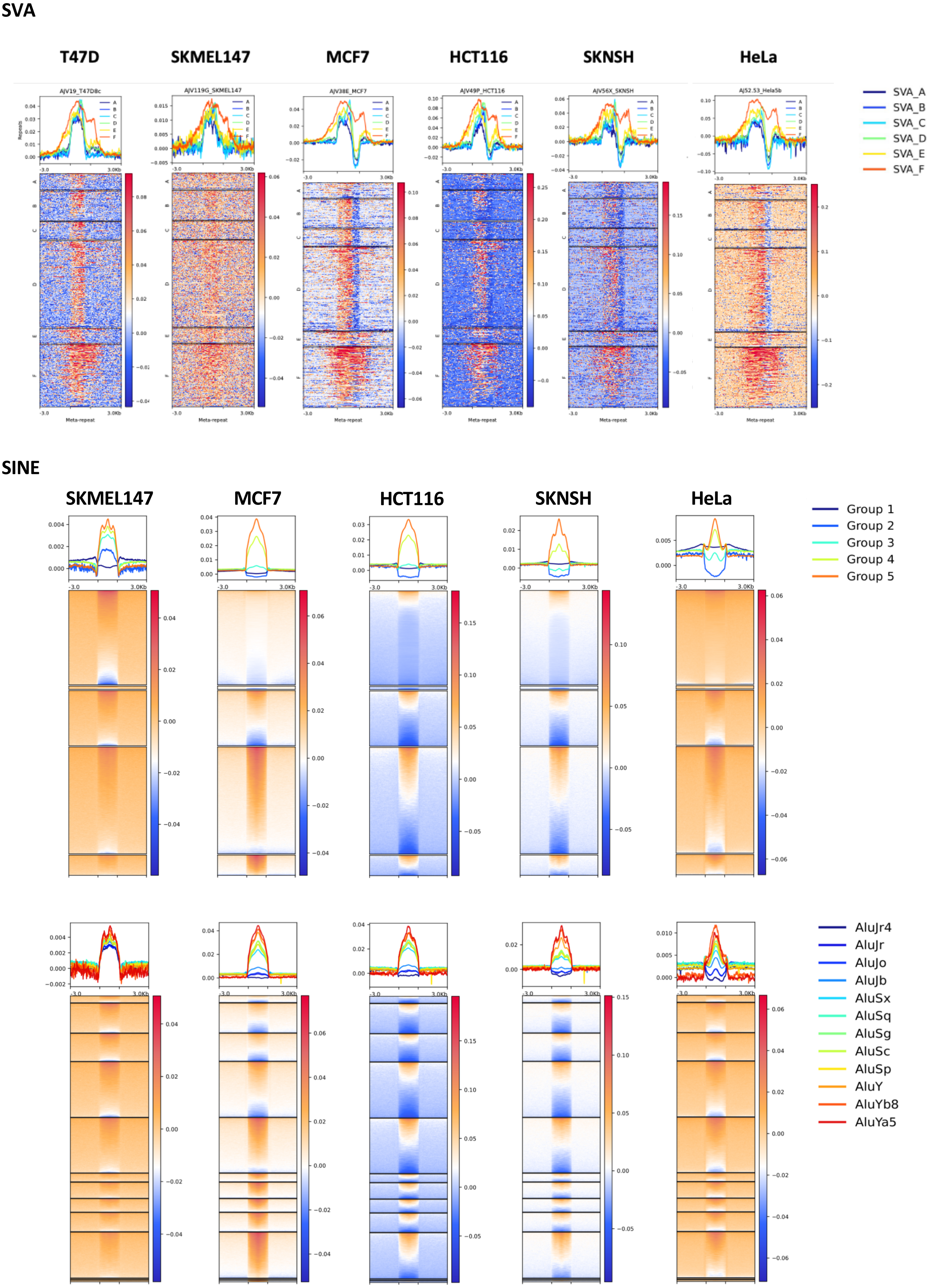

Suppl. Figure S17

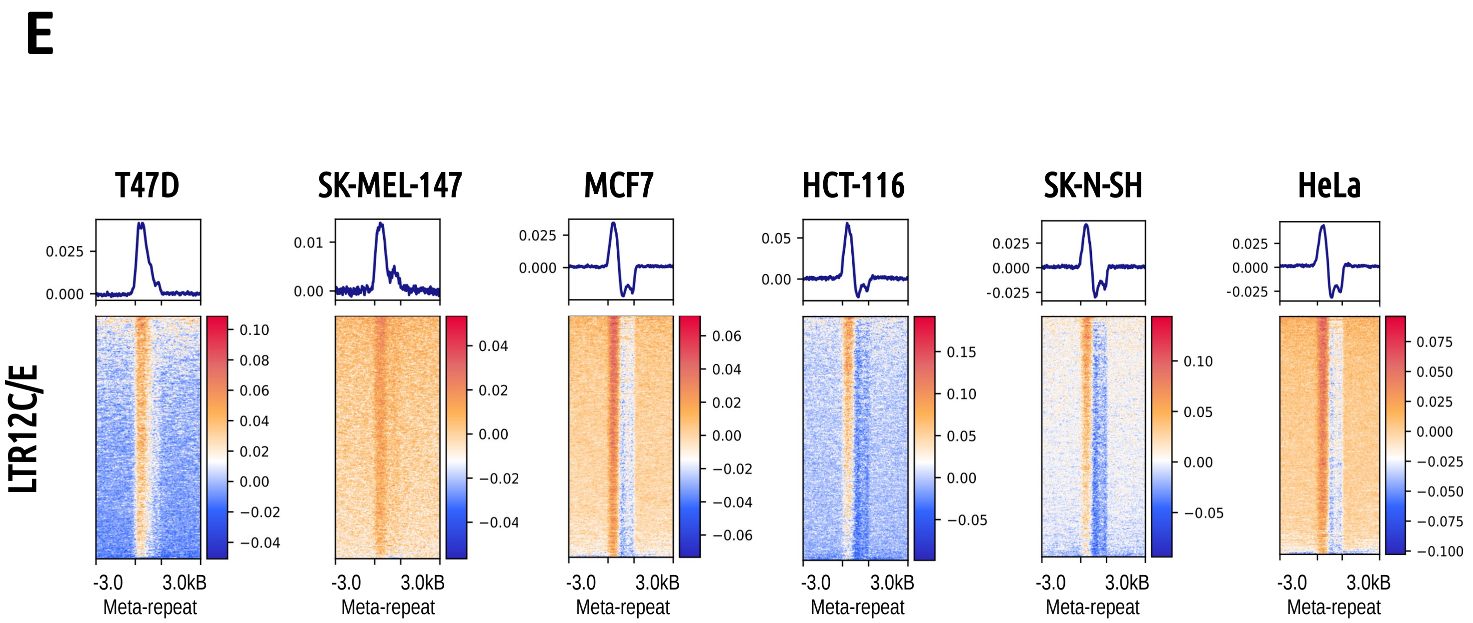

Suppl. Figure S18

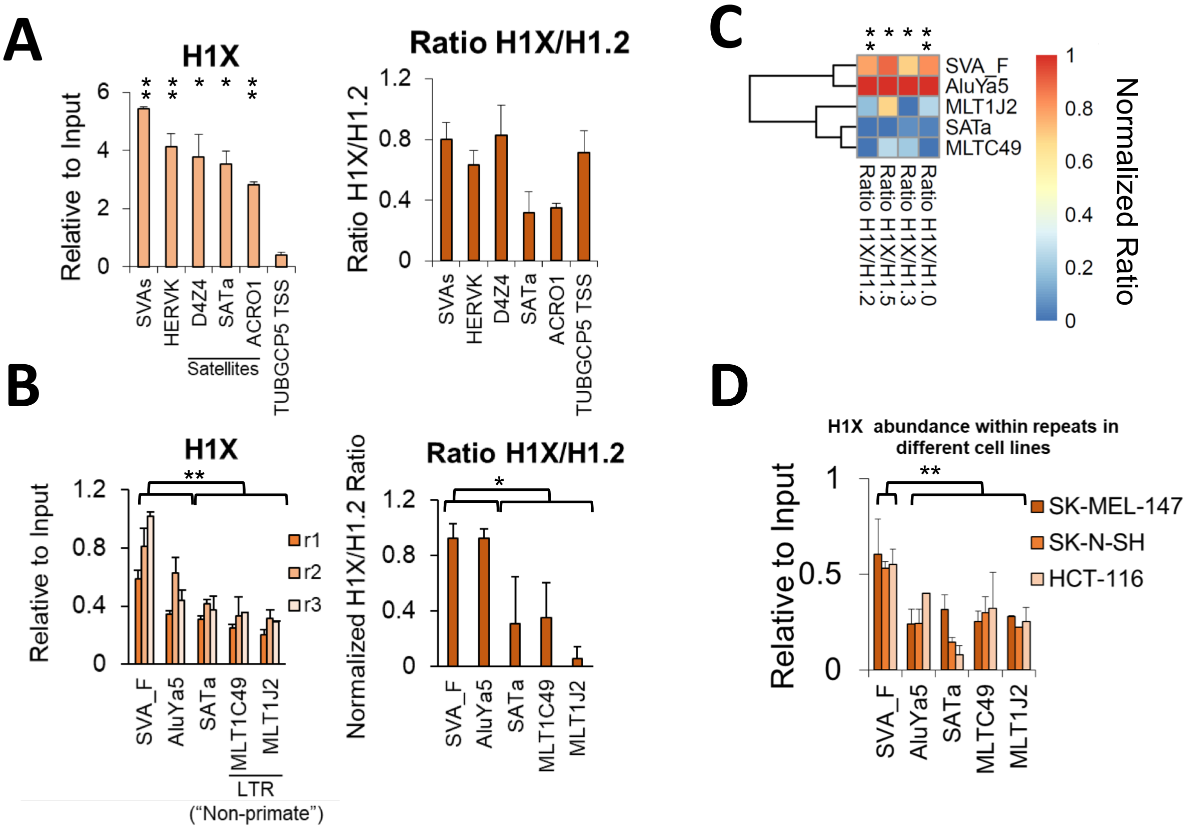

Suppl. Figure S19

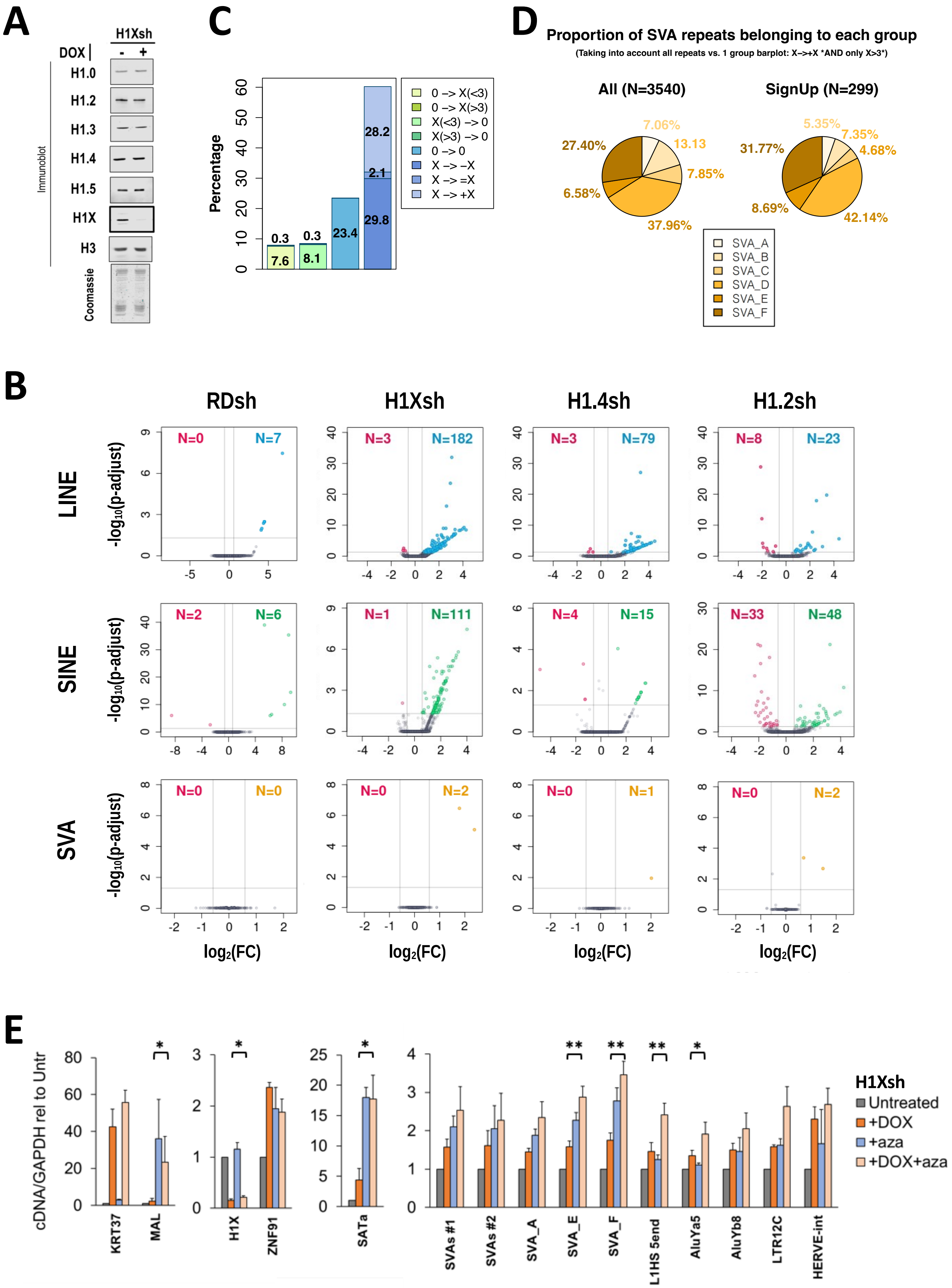

Supplement: gkae014_Supplemental_File [file gkae014_supplemental_file.pdf]
